# Automated solid-phase synthesis of oligosaccharides containing sialic acids

**DOI:** 10.3762/bjoc.11.69

**Published:** 2015-05-04

**Authors:** Chian-Hui Lai, Heung Sik Hahm, Chien-Fu Liang, Peter H Seeberger

**Affiliations:** 1 Department of Biomolecular Systems, Max-Planck-Institute of Colloids and Interfaces, Am Mühlenberg 1, 14476 Potsdam, Germany; 2 Freie Universität Berlin, Institute of Chemistry and Biochemistry, Arnimallee 22, 14195 Berlin, Germany

**Keywords:** α-sialylation, automated synthesis, glycosylation, sialic acid, solid-phase synthesis

## Abstract

A sialic acid glycosyl phosphate building block was designed and synthesized. This building block was used to prepare α-sialylated oligosaccharides by automated solid-phase synthesis selectively.

## Introduction

**Figure 1:**
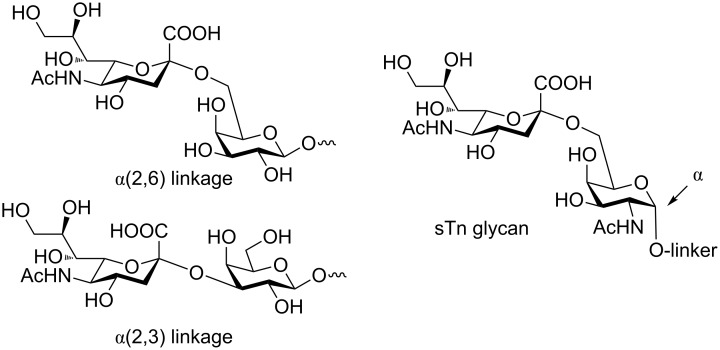
Terminal sialic acids are typically α-(2,3) or α-(2,6) linked to galactose (Gal) such as in the tumour-associated antigen sialyl Tn (sTn).

*N*-Acetylneuraminic acid (sialic acid, Neu5Ac) is an important component of mammalian glycans and key to many recognition events of biomedical relevance including cell–cell recognition, signaling, and the immune response [1]. Sialic acids are present in tumor-associated carbohydrate antigens (TACAs) such as the sialyl-Tn antigen (sTn) [2]. Neu5Ac is often the terminal residue and is usually linked via an α-(2,3) or α-(2,6) linkage to galactose (Gal) (Figure 1) [3].

Automated glycan assembly enables rapid access to structurally defined oligosaccharides [4–5] including glycopeptides [6], glycosaminoglycans [7–9], and chains as long as 30-mers [10]. Key to automated assembly is the identification of reliable monosaccharide building blocks to construct particular linkages. To date, α-(2,3)- and α-(2,6)-sialylated glycans have been accessible by automation only via incorporation of sialic acid–galactose disaccharide building blocks [5,11]. Here, we describe a sialic acid building block that can be utilized for automated glycan assembly.

## Results and Discussion

Sialylating oligosaccharides in high yield and α-selectivity was challenging since the presence of a C-1 carboxyl electron-withdrawing group at the quaternary anomeric center decreases the reactivity. In addition, no participating group on C-3 can be used to direct the stereochemistry at the anomeric carbon (C-2) [2]. Efficient chemical sialylation reactions utilize the cyclic 4*O*,5*N*-oxazolidinone protecting group [12–15], where the *trans*-fused cyclic protecting group in the glycosylation transition state likely stabilizes the positive charge on the intermediate acetonitrile adduct and decreases the generation of a positive charge at the anomeric center by their strong dipole moment [2,16–17].

**Scheme 1:**
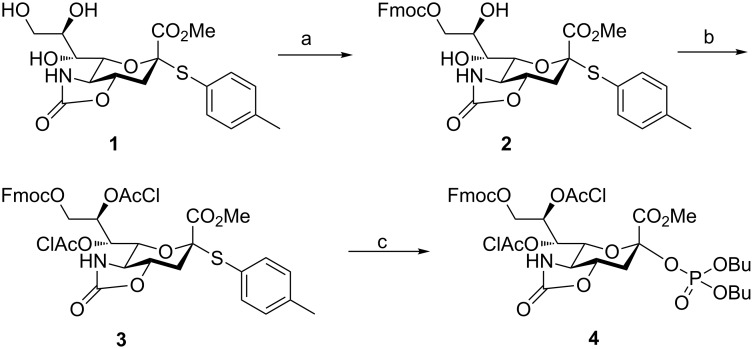
(a) FmocCl, py, CH_2_Cl_2_, rt, 4 h, 77%, (b) 2-chloroacetyl chloride, py, CH_2_Cl_2_, 0 °C to rt, 3 h, 88%, (c) HOPO(OBu)_2_, NIS, TfOH, 4 Å MS, CH_3_CN/CH_2_Cl_2_, −78 °C to 0 °C, 2 h, 80%.

Based on these considerations sialyl phosphate building blocks **4** and **5** [14] were selected for automated glycan assembly using monosaccharides (Scheme 1). The synthesis of building block **4** commenced with the placement of a C-9 Fmoc protecting group on thioglycoside **1** [14] to produce **2**. Installation of *O*-chloroacetyl groups on C-7 and C-8 for better α-stereoselectivity [12] produced **3**. An α-anomeric phosphate leaving group was chosen since it had previously shown high reactivity [14,18] and selectivity [15]. Building block **4** was obtained in 54% yield over three steps from **1**.

“Approved building blocks” for automated glycan assembly have to be accessible in sufficient quantities, stable for storage and activated at a specific temperature to provide the desired linkage in high yield. The optimal glycosylation temperature was determined to ensure fast and efficient reactions at the highest possible temperature [19–20]. Rather than slowly warming a reaction mixture as is done in solution phase, on the automated synthesizer, the building block will be delivered at the optimal temperature and reacted for a predetermined time. For sialic acid building block **4**, the activation temperature was determined to be −20 °C (Table S1 in Supporting Information File 1). The synthesis of trisaccharides **14** illustrates how optimization of the activation temperature resulted in increased yields (Table S4 in Supporting Information File 1).

**Scheme 2:**
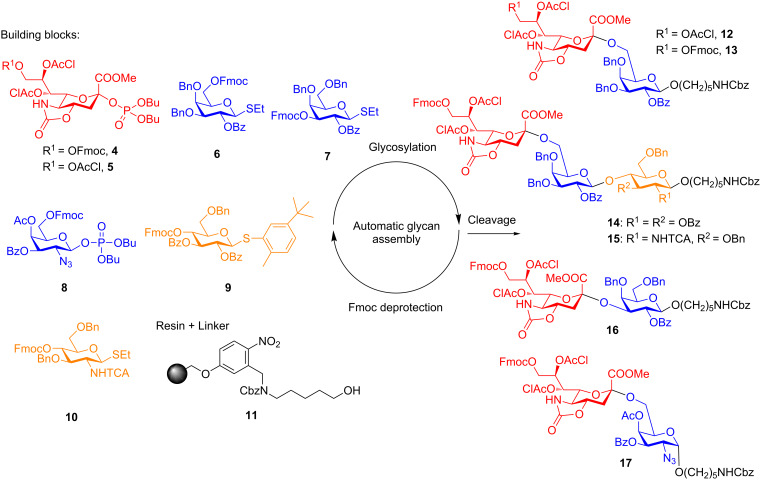
Automated synthesis of oligosaccharides with α(2,3)-, α(2,6)-sialic acid linkages. Glycosylations: a) 2 × 5 equiv TMSOTf, ACN/DCM (1:1), −50 °C (5 min), −30 °C (10 min), −20 °C (80 min), −10 °C (10 min), 0 °C (10 min) for **4** and **5**. b) 2 × 5 equiv TfOH, NIS, DCM, −40 °C (5 min), −20 °C (30 min) for **6** and **7**. c) 2 × 5 equiv TMSOTf, DCM/dioxane (3:2), 20 °C (90 min), for **8**. d) 2 × 5 equiv TfOH, NIS, DCM, −30 °C (5 min), −10 °C (25 min) for **9** and **10**. Fmoc Deprotection: e) 3 × 20% NEt_3_ in DMF, 5 min. Photocleavage: f) UV irradiation using a continuous flow reactor, DCM, rt. Synthesis of **12** or **13**: (1) **6**, b, (2) e, (3) **4** or **5**, a (4) f, 30% for four steps to yield **12**, 40% for four steps to yield **13**; synthesis of **14**: (1) **9**, d, (2) e, (3) **6**, b, (4) e, (5) **4**, a, (6) f, 22% for six steps; synthesis of **15**: (1) **10**, d, (2) e, (3) **6**, b, (4) e, (5) **4**, a, (6) f, 7% for six steps; synthesis of **16**: (1) **7**, b, (2) e, (3) **4**, a, (4) f, 19% for four steps; synthesis of **17**: (1) **8**, c, (2) e, (3) 3 × Ac_2_O, py, 25 °C for 60 min, (4) **4**, a, (5) f, 10% for five steps.

Six di- and trisaccharides (**12**–**17**, Scheme 2) served as targets to develop an automated method for chemical sialylation. Monosaccharide building blocks **4**, **5** [14], **6**, **7** [21], **8**, **9** [21], and **10** [5] were employed for these syntheses. Merrifield polystyrene resin equipped with a photocleavable linker, **11**, was placed in the reaction chamber of the automated synthesizer and the coupling cycles were initiated following programmed maneuvers. Each cycle starts with a TMSOTf acidic wash at −20 °C to ensure that no base from previous deprotection reactions remains and quenches the subsequent coupling. This problem had been observed earlier (data not shown) and can be overcome by this extra washing step. In addition, TMSOTf eliminates any moisture that may have resided on the resin or in the reaction vessel.

Glycosylations were carried out using the optimized temperatures for each building block using twice five equivalents of building block and activator. Removal of the Fmoc protecting group with triethylamine uncovered the hydroxy group to serve as the nucleophile in the next coupling. Participating protecting groups at the C2 position of building blocks **6**, **7**, **9** and **10** ensured selective formation of β-glycosidic linkages during the glycosylations. These building blocks resulted in complete conversion as determined by Fmoc quantification [5] and HPLC analysis.

Sialyl phosphate building blocks **4** and **5** resulted in good α-selectivity for the installation of α-(2,6)-linkages in disaccharides **12** and **13**, both sialyl phosphate building blocks **4** and **5** showed exclusive α-selectivity. However, building block **4** was more reactive than **5** as the synthesis of disaccharide **13** resulted almost in full α-sialylation as observed by HPLC analysis of the crude product following photocleavage from the resin that showed only one peak while **12** was not the only product. Disaccharide **12** was obtained in 30% and **13** in 40% overall yield for four steps based on resin loading. The absolute anomeric configurations of glycans that contain sialylic acid were determined by recording the long-range coupling constants of C1 with axial H3 (^3^*J* C-1,H-3_ax_) using 1D coupled HMQC experiments. Coupling constant higher than 5 Hz correspond to α-configurations [12].

Two trisaccharides (**14** and **15**) that are α-(2,6)-sialylated were obtained in 22% and 7% yield after HPLC purification based on resin loading for six steps. The sialylation proceeded with α-stereoselectivity in both cases. The synthesis of **14** was higher yielding than **15**. The major structural difference of **14** and **15** is the first sugar attached on the resin. The *N*-protecting TCA group of glucosaminoside has more electron-withdrawing character in the synthesis of **15** than the benzoate ester groups of the glucoside in the synthesis of **14** which resulted in a less favorable sialylation for **15**.

To demonstrate that α-(2,3)-sialylations are possible, model disaccharide **16** was synthesized in 19% yield. The secondary C3 hydroxy group in galactose is less reactive and consequently, even after optimization, the chemical sialylation of the C3 position of galactose did not result in a satisfactory yield and demonstrates a current limitation of the automated glycan assembly approach. Recently, placement of an isothiocyanate moiety on the C5 position was reported to be an effective method to construct alpha linkages [22] and may prove useful for solid-phase synthesis in the future as well.

The tumor associated sTn carbohydrate antigen (Neu5Ac-α(2,6)GalNAc-α(1,1)linker) disaccharide **17**, that resembles the sTn antigen glycan framework (Neu5Ac-α(2,6)GalNAc-α(1,1)Ser/Thr) was synthesized. In order to install the *cis*-glycoside formed by the union of the galactosamine and the linker, galactosamine building block **8** relies on remote participating protecting group effects of esters at C3 and C4 [23–24]. The selectivity of the *cis*-glycosylation improved with higher reaction temperatures due the strongly deactivating effect of three electron withdrawing ester and carbonate protecting groups [23,25]. The addition of dioxane to CH_2_Cl_2_ resulted in preferred formation of the α-anomer, an effect that is well known from solution phase syntheses [26] (Table S6, Figure S1 in Supporting Information File 1). When five equivalents of building block **8** were used at 20 °C for 90 min with a solvent ratio of CH_2_Cl_2_ and dioxane of 3:2, mainly the desired α-anomer was obtained (2:1). A double coupling of building block **8** to install the α-galactosamine linker was followed by a capping step. Incorporation of building block **4**, cleavage from the resin and purification by HPLC yielded disaccharide **17** in 10% yield.

## Conclusion

In summary, we demonstrated that a 5*N*,4*O-*carbonyl-7,8-di-*O*-chloroacetyl-9-*O*-Fmoc-protected sialic acid phosphate building block **4** can be used to install α(2,6)-sialic acid linkages efficiently, while it did not give satisfactory results for α(2,3)-sialylations. The latter linkage has to be incorporated either by using a preformed sialic acid–Gal disaccharide building block [11] or by enzymatic sialylation [27] following the cleavage and deprotection of an oligosaccharide.

## Supporting Information

File 1 Experimental part.
