# Supplementary material for: Automated solid-phase synthesis of oligosaccharides containing sialic acids
Source: Beilstein J Org Chem. 2015 May 4;11:617–21. doi: 10.3762/bjoc.11.69 (PMC4464161; doi:10.3762/bjoc.11.69)
Supplement: File 1 — Experimental part. [file Beilstein_J_Org_Chem-11-617-s001.pdf]

Supporting Information

for

Automated solid-phase synthesis of oligosaccharides containing sialic acids

Chian-Hui Lai[1], Heung Sik Hahm [1,2], Chien-Fu Liang[1] and Peter H. Seeberger[1, 2*§]

Address: [1]Department of Biomolecular Systems, Max-Planck-Institute of Colloids and Interfaces, Am Mühlenberg 1, 14476 Potsdam, Germany and [2]Freie Universität Berlin, Institute of Chemistry and Biochemistry, Arnimallee 22, 14195 Berlin, Germany

Email: Peter H. Seeberger - peter.seeberger@mpikg.mpg.de

[§]Fax: +49 30 838-59302; Tel: +49 30 838-59301

*Corresponding author

Experimental part

Table of Contents

Building block synthesis S3

Synthesis of sialic acid phosphate building block **4**. S3

Synthesis Gal thioglycoside building block **6**. S12

Synthesis of GalN phosphate building block **8**. S19

Optimization of sialic acid building block activation temperature S32

Automated synthesis of sialylated oligosaccharides S33

General materials and methods........ S33

Preparation of reagent solutions S33

Modules for automated synthesis S34

Post-synthesizer manipulations S35

Automated synthesis of **12** S35

Automated synthesis of **13** S40

Automated synthesis of **14** S44

Automated synthesis of **15** S48

Automated synthesis of **16** S52

Discussion of first glycosylation: *cis*-glycosidic linkage for **17**........ S57

Automated synthesis of **17** S58

List of Figures

Figure S1. The NMR spetrum of crude **S9** in different conditions for first glycosylation….S57

Figure S2. The comparison ^{1}H-NMR spectrum of **17** and side its side product (β-GalN to linker)……..S59

List of Tables

Table S1. The optimization of sialic acid activation temperature was determined by mass-spectrometry…….S32

Table S2. Automated synthesis program for disaccharide **12**…….S35

Table S3. Automated synthesis program for tri-saccharide **14**……..S44

Table S4. Different sialylation temperatures for synthesis of **14.** ……..S44

Table S5. Automated synthesis program for disaccharide **16**. ……..S53

Table S6. Different solvents and temperature conditions for the first α-GalN to linker glycosylation in synthesis of precursor of **17**…….S57

Table S7. Automated synthesis program protocol for disaccharide **17**...……...S58

General materials and methods

All chemicals used were reagent grade and used as supplied except where noted. Anhydrous solvents used were taken from a dry solvent system (jcmeyer-solvent systems). Analytical thin layer chromatography (TLC) was performed on Merck silica gel 60 F254 plates (0.25 mm). Compounds were visualized by UV irradiation or dipping the plate in an anisaldehyde

sugar stain solution. Flash column chromatography was carried out using forced flow of the indicated solvent on Fluka Kieselgel 60 Å (230-400 mesh). Purification by normal/reverse phase HPLC was performed using Agilent 1200 series. Optical rotations were measured using Perkin-Elmer 241 and Unipol L1000 polarimeters. IR spectra were recorded on a Perkin-Elmer 1600 FTIR spectrometer. ^{1}H, ^{13}C spectra were recorded on a Varian 400-MR (400 MHz) and/or Varian 600-MR (600 MHz) spectrometer in $CDCl_3$ (δ, 7.24), CD_3OD (δ, 3.31), D_2O (δ, 4.80). NMR chemical shifts (δ) are reported in ppm and coupling constants (*J*) are reported in Hz. High resolution mass spectra were obtained with a 6210 ESI-TOF mass spectrometer (Agilent).

Building block synthesis

Synthesis of sialic acid phosphate building block 4.

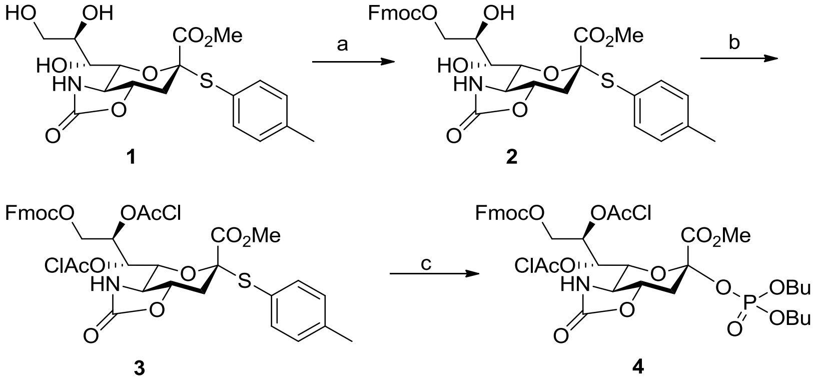

Scheme 1. (a) FmocCl, py, CH_2Cl_2, rt, 4 h, 77%, (b) 2-chloroacetyl chloride, py, CH_2Cl_2, 0 ^oC to rt, 3 h, 88%, (c) $HOPO(OBu)_2$, NIS, TfOH, 4A MS, CH_3CN/ CH_2Cl_2, -78 ^oC to 0 ^oC, 2 h, 80%

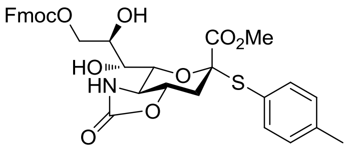

2

Methyl (4-methylphenyl 5-amino-5-*N*,4-*O*-carbonyl-9-*O*-fluorenylmethoxycarbonyl -3,5-dideoxy-2-thio-D-*glycero*-α-D-*galacto*-non-2-ulopyranoside)onate (2)

To a solution of compound **1**[1, 2] (1.08 g, 2.61 mmol) in CH_2Cl_2 (26.1 mL, 0.1 M), FmocCl (0.81 g, 3.13 mmol) and pyridine (2.11 mL, 26.1 mmol) were added at room temperature under Ar. After stirring for 4 h, the reaction mixture was neutralized with 1 M HCl, and then extracted with brine and dried over $MgSO_4$. After the solution was concentrated, the residue

was purified by flash silica gel column chromatography (Hex/EtOAc/MeOH, form 1/1/0 to 1/1/0.06) to give product **2** as white powder (1.28 g, 77%). R_f = 0.61 (Hex/ EtOAc/ MeOH = 1/1/0.1); $[\alpha]_D^{20}$ = + 20.3 (c = 1.0, CH_2Cl_2); ^{1}H NMR (400 MHz, $CDCl_3$) δ 2.19 (t, *J* = 12.3 Hz, 1H, H-3_{ax}), 2.31 (s, 3H, Ph-CH_3× 3), 3.14 (dd, *J* = 12.3, 3.6 Hz, 1H, H-3_{eq}), 3.46 – 3.61 (m, 2H, H-5, H-6), 3.62 (s, 3H, OCH_3), 3.77 (dd, *J* = 9.7, 3.3 Hz, 1H, H-7), 3.83-4.01 (m, 2H, H-4, H-8), 4.25 (t, *J* = 7.3 Hz, 1H, C*H*-Fmoc), 4.36 (dd, *J* = 11.6, 5.7 Hz, 1H, H-9), 4.55 – 4.40 (m, 3H, H-9, CH_2-Fmoc×2), 5.92 (b, 1H, N*H*), 7.13 (d, *J* = 7.9 Hz, 2H), 7.35 – 7.26 (m, 4H), 7.39 (t, *J* = 7.4 Hz, 2H), 7.61 (d, *J* = 7.5 Hz, 2H), 7.74 (d, *J* = 7.5 Hz, 2H); ^{13}C NMR (101 MHz, $CDCl_3$) δ 21.3 (Ph-CH_3), 36.9 (C-3), 46.7 (*C*H-Fmoc), 53.3 (OCH_3), 57.4 (C-5), 69.0, 70.0, 70.1, 70.6, 78.3 (C-7), 78.8 (C-4), 87.6 (C-2), 120.1, 124.5, 125.1, 127.2, 127.9, 129.8, 136.5, 141.3, 143.3, 155.7 (C=O), 159.8 (C=O), 168.8 (C=O); IR (thin film) 3487, 3065, 2953, 1746, 1491, 1478, 1450, 1390, 1265, 1234, 1193, 1168, 1151, 1133, 1106, 1080, 1036, 1004, 969, 939, 910, 812, 787, 759, 737 cm^{-1}; HRMS (ESI) calcd. for $C_{33}H_{33}O_{10}SNNa$ $(M+Na)^+$ 658.1723 , found 658.1764.

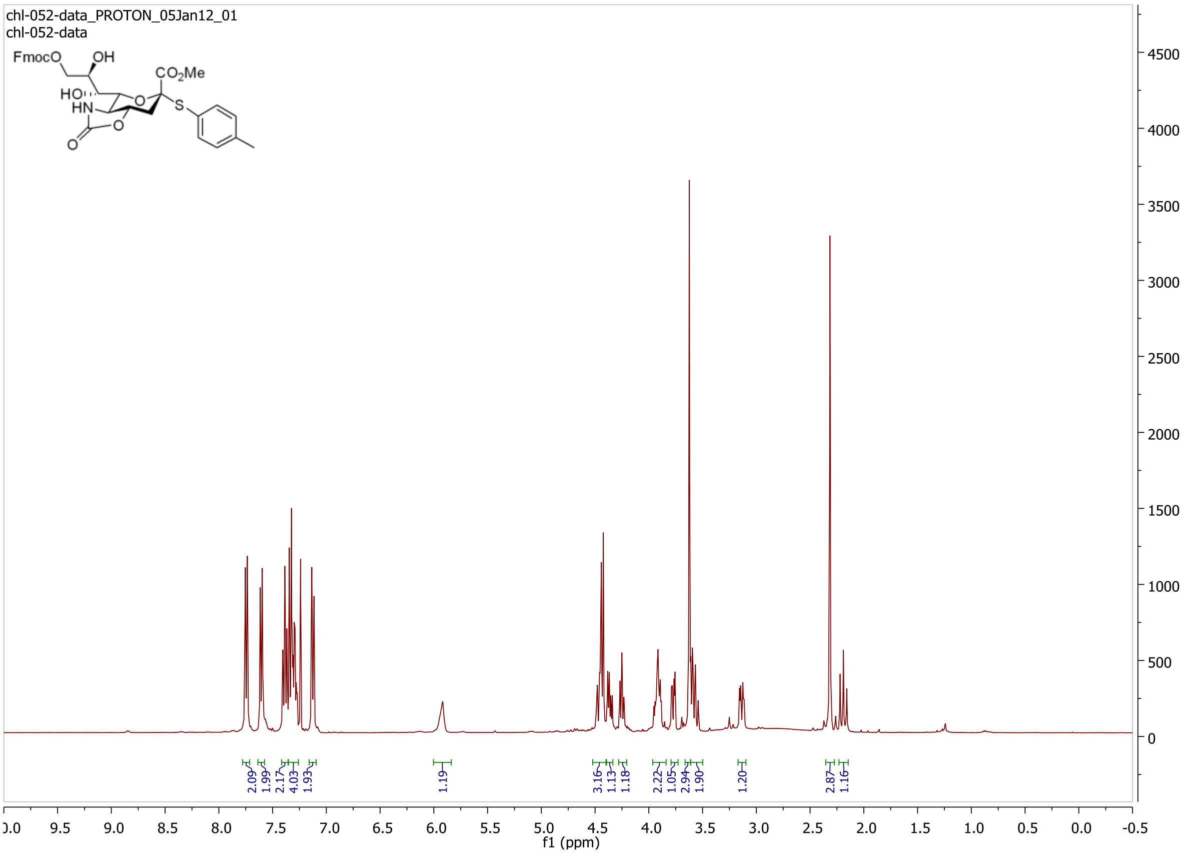

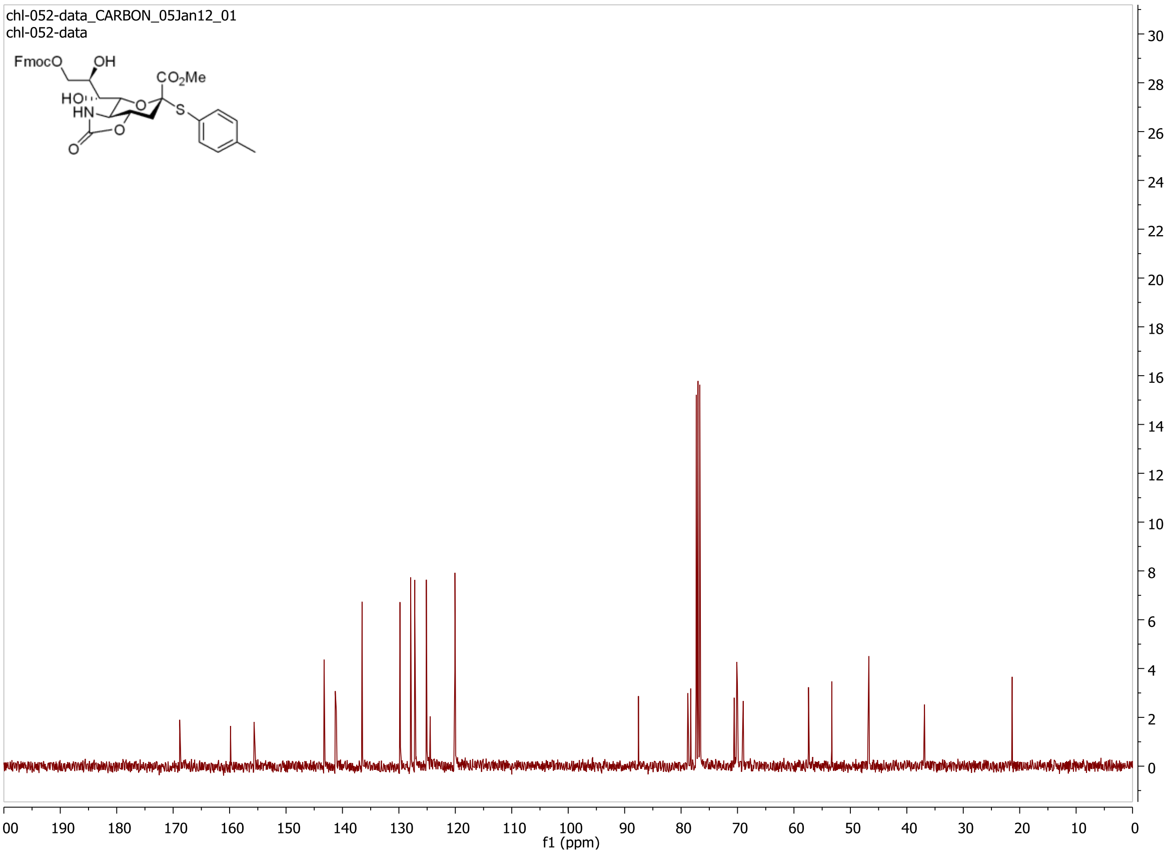
chl-052-data_CARBON_05Jan12_01
chl-052-data
FmocO
OH
CO2Me
HO
HN
O
S
f1 (ppm)

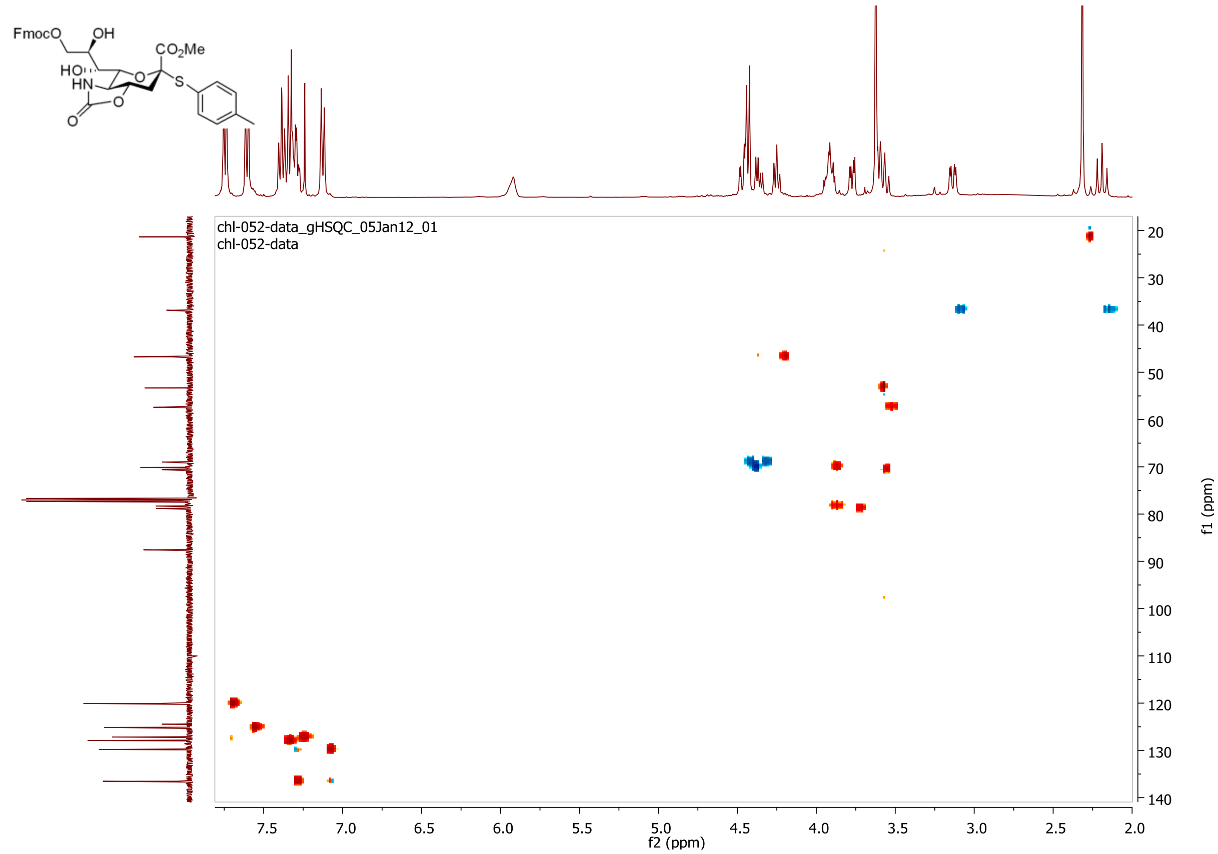
FmocO
OH
CO2Me
HO
HN
O
S
chl-052-data_gHSQC_05Jan12_01
chl-052-data
f2 (ppm)
f1 (ppm)

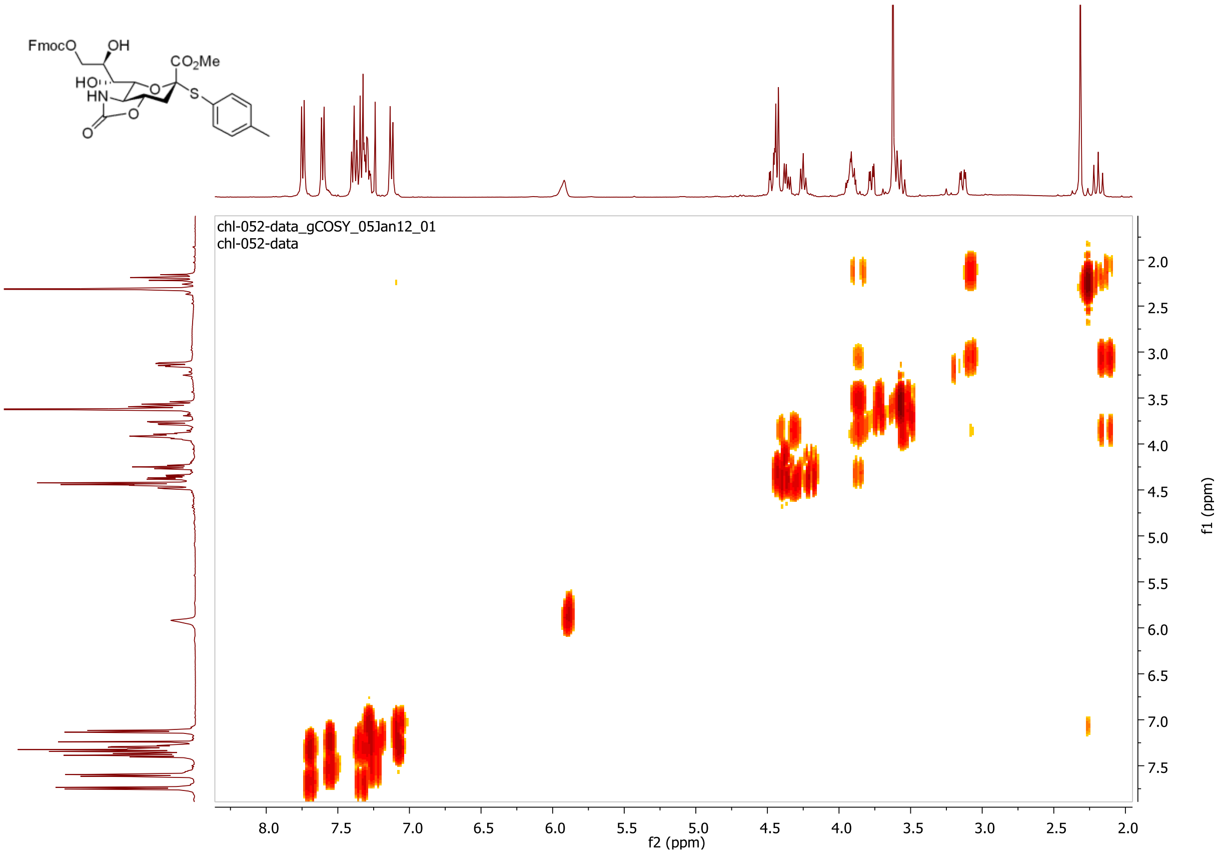

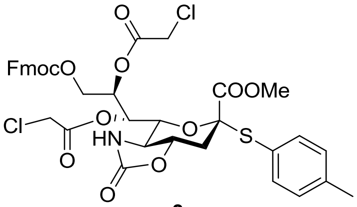

3

Methyl (4-methylphenyl 5-amino-5-*N*,4-*O*-carbonyl-7,8-di-*O*-chloroacetyl-9-*O*-fluorenyl methoxycarbonyl-3,5-dideoxy-2-thio-D-*glycero*-α-D-*galacto*-non-2-ulopyranoside)onate (3)

To a solution of compound **2** (2.20 g, 3.46 mmol) in CH_2Cl_2 (35 mL, 0.1 M), pyridine (1.40 mL, 17.30 mmol) was added at room temperature under Ar and the resulting solution was cooled to 0 °C. To the stirred reaction mixture, chloroacetyl chloride (0.83 mL, 10.38 mmol) in CH_2Cl_2 (20 mL) was added dropwise through an addition funnel. The color of the reaction mixture changed from pale yellow to orange. After being stirred at room temperature for 3 hours, the reaction mixture was poured into 1M aqueous cold HCl solution (40.0 mL). The aqueous layer was extracted with CH_2Cl_2 three times. The combined extracts were washed with brine and dried over $MgSO_4$. The solution was concentrated under reduced pressure and then subjected to silica gel column chromatography (Hex/EtOAc, form 8/1 to 5/3) to give product **3** as a white powder (2.40 g, 88%). R_f = 0.35 (Hex/EtOAc = 2/1) ; $[\alpha]_D^{20}$ = +7.9 (c = 1.0, CH_2Cl_2); ^{1}H NMR (400 MHz, $CDCl_3$) δ 2.13 (t, J = 12.5 Hz, 1H, H-3_{ax}), 2.34 (s, 3H,

Ph-CH_3× 3), 3.00 (t, J = 10.4 Hz, 1H, H-5), 3.10 (dd, J = 12.5, 3.6 Hz, 1H, H-3_{eq}), 3.58 (s, 3H, OCH_3), 3.89 (ddd, J = 12.5, 10.4, 3.6 Hz, 1H, H-4), 4.06 (d, J = 15.4 Hz, 1H, CH_2Cl×1), 4.11 – 4.01 (m, 1H, H-6), 4.14 (d, J = 14.7 Hz, 1H, CH_2Cl×1), 4.16 (d, J = 15.4 Hz, 1H, CH_2Cl×1), 4.19 (d, J = 14.7 Hz, 1H, CH_2Cl×1), 4.25 (t, J = 7.4 Hz, 1H, C*H*-Fmoc), 4.37 (dd, J = 10.4, 7.4 Hz, 1H, CH_2-Fmoc×1), 4.42 (dd, J = 10.4, 7.4 Hz, 1H, CH_2-Fmoc×1), 4.48 (dd, J = 12.9, 3.0 Hz, 1H, H-9), 4.55 (dd, J = 12.9, 2.3 Hz, 1H, H-9), 5.20 (dd, J = 9.5, 1.3 Hz, 1H, H-7), 5.26 (s, 1H, N*H*), 5.42 (dt, J = 9.5, 2.3 Hz, 1H, H-8), 7.14 (d, J = 7.9 Hz, 2H), 7.35 – 7.28 (m, 4H), 7.45 – 7.38 (m, 2H), 7.62 – 7.53 (m, 2H), 7.76 (d, J = 7.5 Hz, 2H); ^{13}C NMR (101 MHz, $CDCl_3$) δ 21.3 (Ph-*C*H_3), 37.5 (C-3), 40.4 (*C*H_2Cl), 40.8 (*C*H_2Cl), 46.6 (*C*H-Fmoc), 53.1 (O*C*H_3), 57.6 (C-5), 64.5 (C-9), 69.4 (C-8), 70.3 (C-7), 70.5 (*C*H_2-Fmoc), 74.9 (C-6), 77.2 (C-4), 88.4 (C-2), 120.1, 124.5, 125.1, 125.1, 127.2, 128.0, 129.9, 136.2, 140.8, 141.3, 143.0, 143.2, 154.9 (C=O), 158.7 (C=O), 166.0 (C=O), 167.8 (C=O), 168.1 (C=O); IR (thin film) 3400, 2954, 1742, 1491, 1478, 1450, 1400, 1259, 1232, 1153, 1102, 1011, 960, 911, 873, 812, 786, 760, 736 cm^{-1}; HRMS (ESI) calcd. for $C_{37}H_{35}O_{12}SNCl_2Na$ $(M+Na)^+$ 810.1155, found 810.1222 .

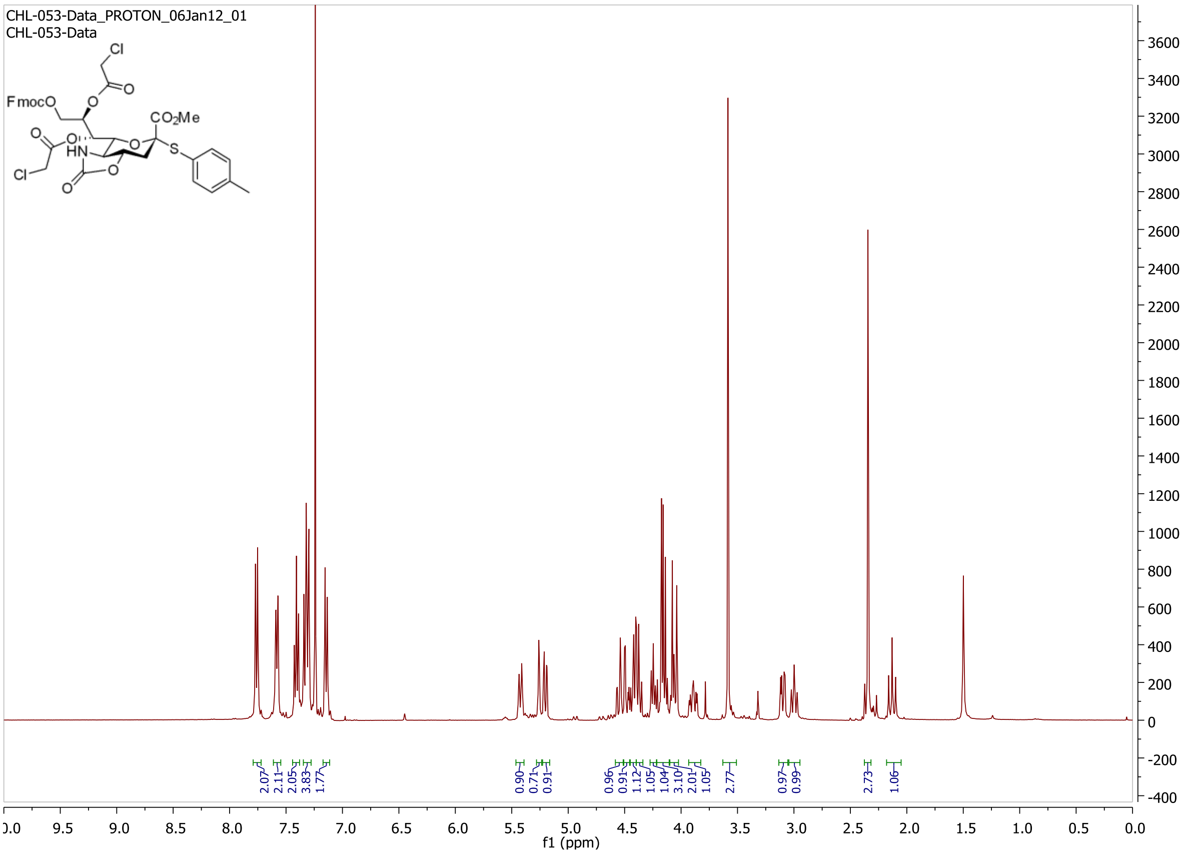

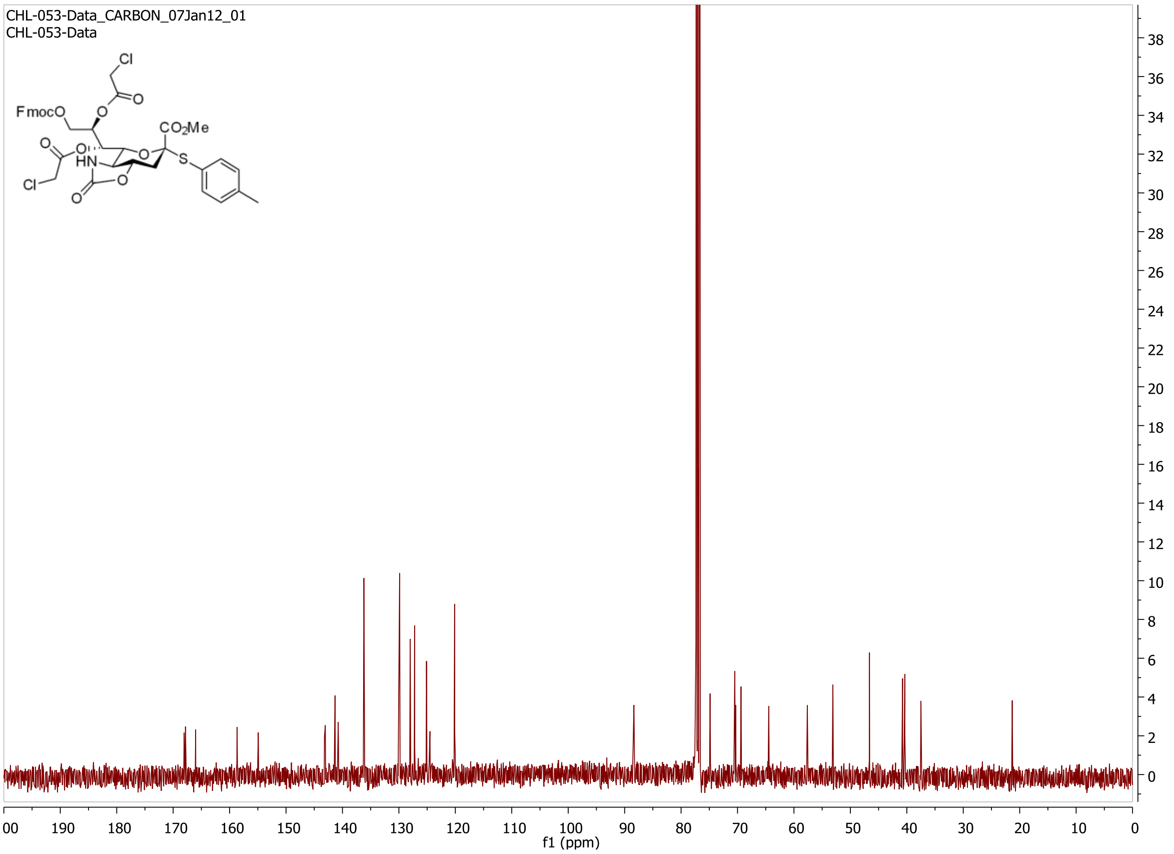
CHL-053-Data_CARBON_07Jan12_01
CHL-053-Data
FmocO
CO2Me
HN
Cl
S
f1 (ppm)

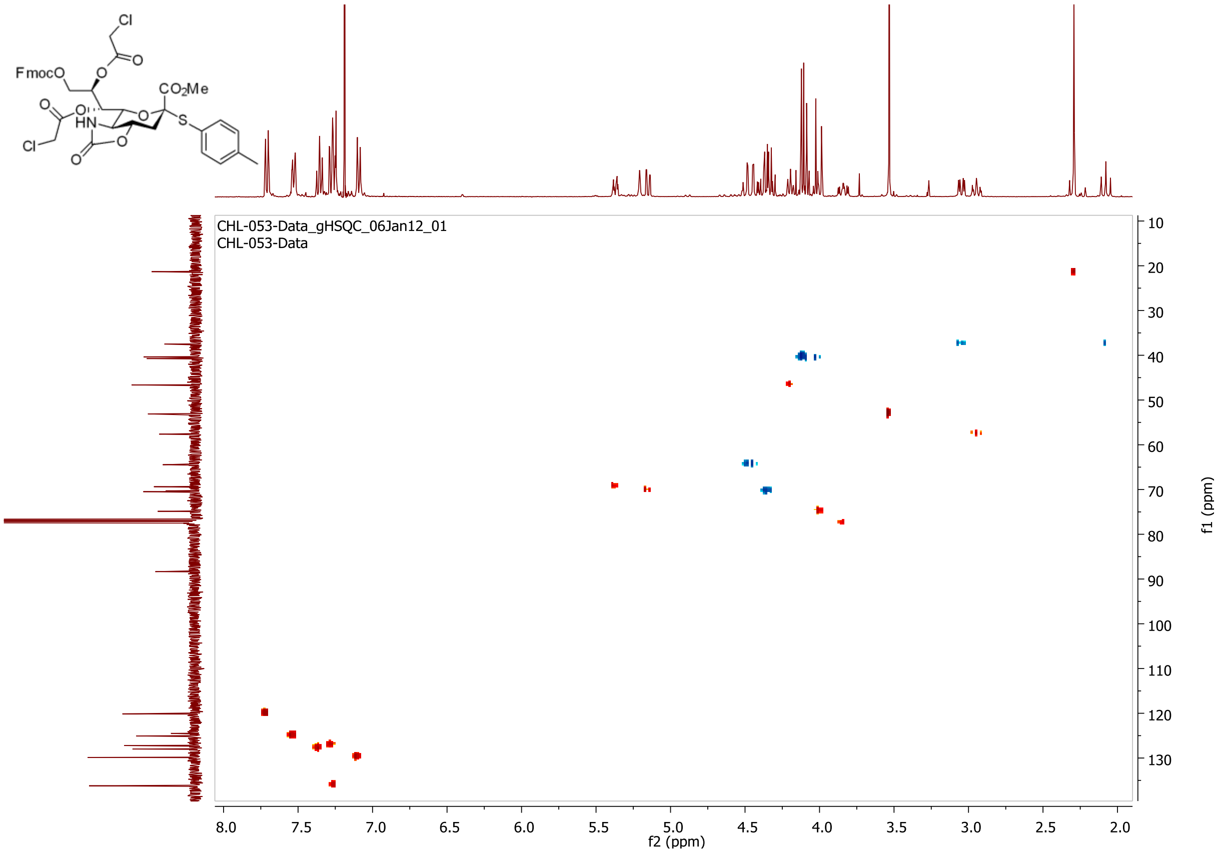
CHL-053-Data_gHSQC_06Jan12_01
CHL-053-Data
FmocO
CO2Me
HN
Cl
S
f2 (ppm)
f1 (ppm)

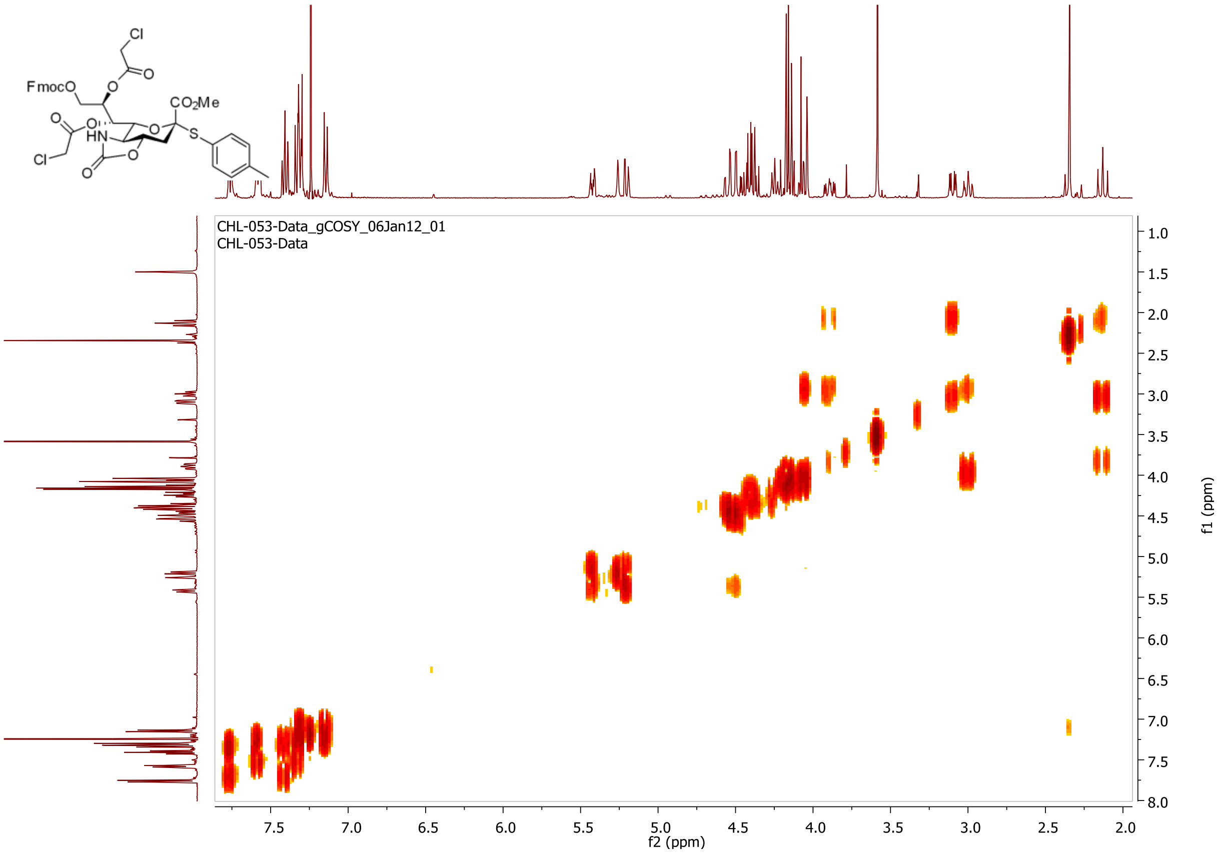

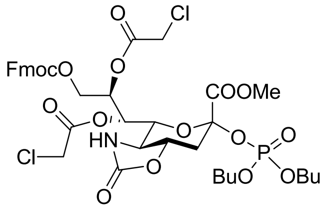

4

Methyl (dibutylphosphate 5-amino-5-*N*,4-*O*-carbonyl-7,8-di-*O*-chloroacetyl-9-*O*-fluorenylmethoxycarbonyl-3,5-dideoxy-D-*glycero*-α-D-*galacto*-non-2-ulopyranoside)onate (4)

Thiosialoside **3** (1.00 g, 1.27 mmol) was dissolved in CH_2Cl_2/MeCN = 1/1 (13 mL, 0.1 M) under Ar. The solution was transferred to a round-bottomed flask containing dry 4 Å MS (0.500 g/mmol) at room temperature under Ar and then $HOPO(OBu)_2$ (0.38 mL, 1.90 mmol) was added. The reaction mixture was stirred for 30 minutes and then cooled to -78 ^{o}C. NIS (0.357 mg, 1.59 mmol) was added followed by TfOH (45 μL, 0.51 mmol). After stirring at -20 ^{o}C for an hour and then warming to room temperature, the reaction mixture was filtrated through Celite. The reaction mixture was quenched and extracted with saturated aqueous $Na_2S_2O_3$ and $NaHCO_3$ solution. The combined extracts were washed with brine, dried over $MgSO_4$ and then concentrated. The residue was purified by silica gel column chromatography (Hex/EtOAc, form 4/1 to 1/1) to give α sialyl phosphate product **4** as white powder (890 mg,

80%). R_f = 0.44 (Hex/ EtOAc = 1/1) ; $[\alpha]_D^{20}$ = - 8.7 (c = 1.0, CH_2Cl_2); ^{1}H NMR (400 MHz, $CDCl_3$) δ 1.05 – 0.69 (m, 6H, C*H*$_3$-Bu × 6), 1.48 – 1.25 (m, 4H, C*H*$_2$-Bu × 4), 1.80 – 1.53 (m, 4H, C*H*$_2$-Bu × 4), 2.64 (t, *J* = 12.1 Hz, 1H, H-3_{ax}), 2.90 (dd, *J* = 12.1, 3.8 Hz, 1H, H-3_{eq}), 3.27 (td, *J* = 10.4, 1.4 Hz, 1H, H-5), 3.82 (s, 3H, OC*H*$_3$), 4.12 – 3.97 (m, 5H, C*H*$_2$-Bu × 4, H-4), 4.15 (d, *J* = 15.3 Hz, 1H, C*H*$_2$Cl×1), 4.20 (s, 2H, C*H*$_2$Cl×2), 4.27 – 4.15 (m, 1H, C*H*-Fmoc), 4.27 (d, J = 15.3 Hz, 1H, C*H*$_2$Cl×1), 4.44 – 4.33 (m, 3H, H-9, C*H*$_2$-Fmoc ×2), 4.53 – 4.46 (m, 2H, H-6, H-9), 5.23 (dd, J = 9.7, 1.6 Hz, 1H, H-7), 5.34 (s, 1H, N*H*), 5.46 (ddd, *J* = 9.7, 3.0, 2.3 Hz, 1H, H-8), 7.32 (td, *J* = 7.5, 0.8 Hz, 2H), 7.41 (t, *J* = 7.5 Hz, 2H), 7.58 (d, *J* = 7.5 Hz, 2H), 7.76 (d, *J* = 7.5 Hz, 2H); ^{13}C NMR (101 MHz, $CDCl_3$) δ 13.6 (*C*H$_3$-Bu ×2), 18.6 (*C*H$_2$-Bu ×2), 32.0 (d, *J* = 3.3 Hz, *C*H$_2$-Bu ×1), 32.1 (d, *J* = 3.7 Hz, *C*H$_2$-Bu ×1), 37.2 (d, *J* = 5.1 Hz, C-3), 40.4 (*C*H$_2$Cl), 40.7 (*C*H$_2$Cl), 46.6 (*C*H-Fmoc), 53.6 (O*C*H$_3$), 56.9 (C-5), 64.5 (C-9), 68.0 (d, *J* = 6.4 Hz, *C*H$_2$-Bu ×1), 68.1 (d, *J* = 6.5 Hz, *C*H$_2$-Bu ×1), 68.9 (C-8), 69.8 (C-7), 70.5 (*C*H$_2$-Fmoc), 75.1 (C-6), 75.9 (C-4), 98.7 (d, *J* = 7.4 Hz, C-2), 120.1, 125.1, 125.1, 127.2, 127.2, 128.0, 128.0, 141.3, 143,0, 143.1, 154.9 (C=O), 158.9 (C=O), 166.1 (C=O), 167.5 (C=O), 168.1 (C=O); IR (thin film) 3398, 2960, 2934, 2874, 1748, 1610, 1580, 1536, 1477, 1450, 1404, 1380, 1257, 1232, 1139, 1091, 1013, 956, 903, 877, 826, 785, 759, 739, 701 cm^{-1}; HRMS (ESI) calcd. for $C_{38}H_{46}O_{16}NCl_2PNa$ $(M+Na)^+$ 873.1931, found 896.1809 m/z.

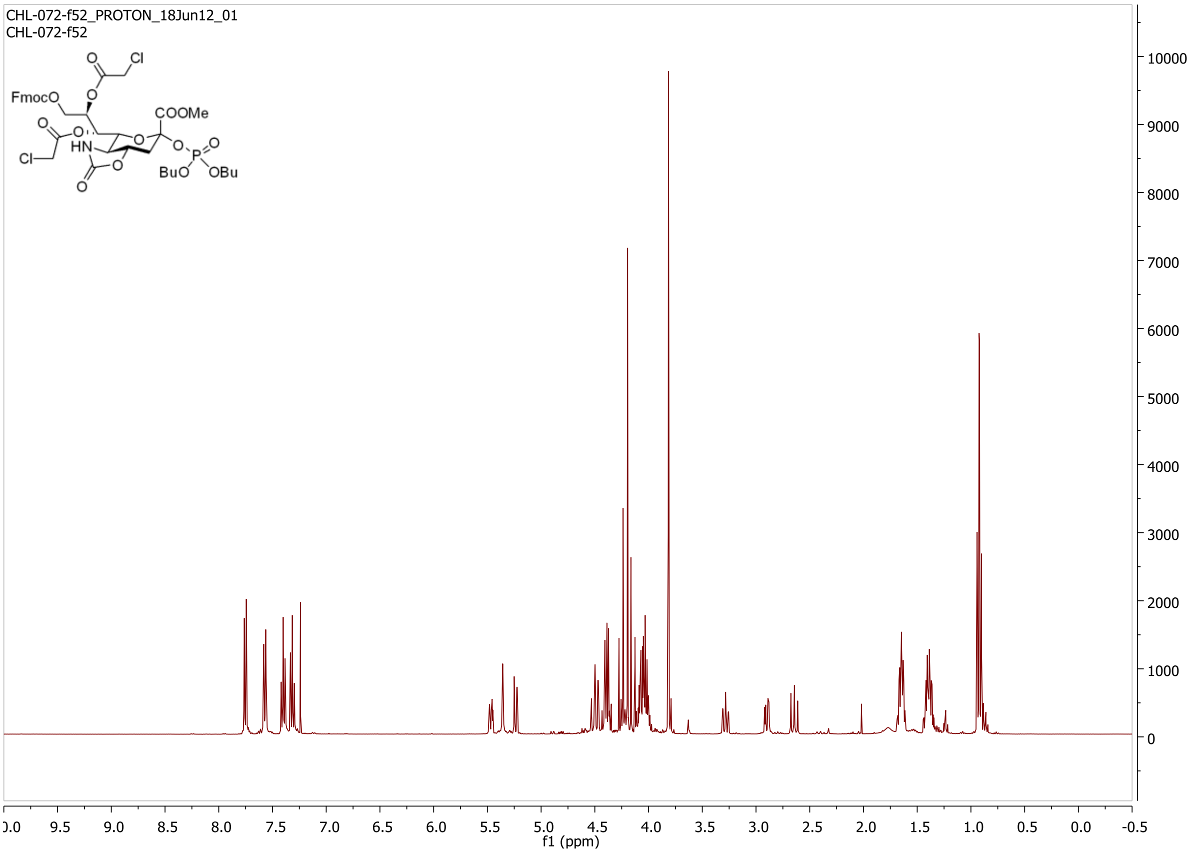

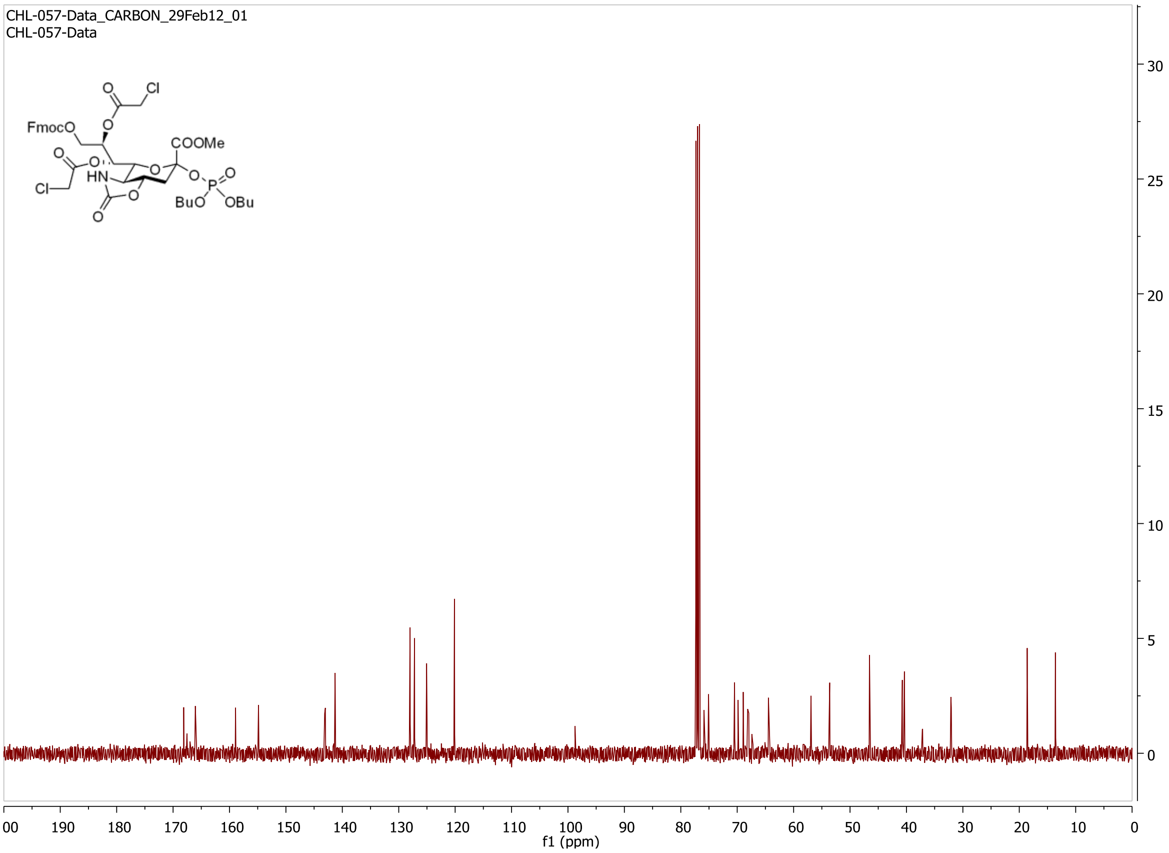
CHL-057-Data_CARBON_29Feb12_01
CHL-057-Data
FmocO
COOMe
HN
BuO
OBu
Cl
f1 (ppm)

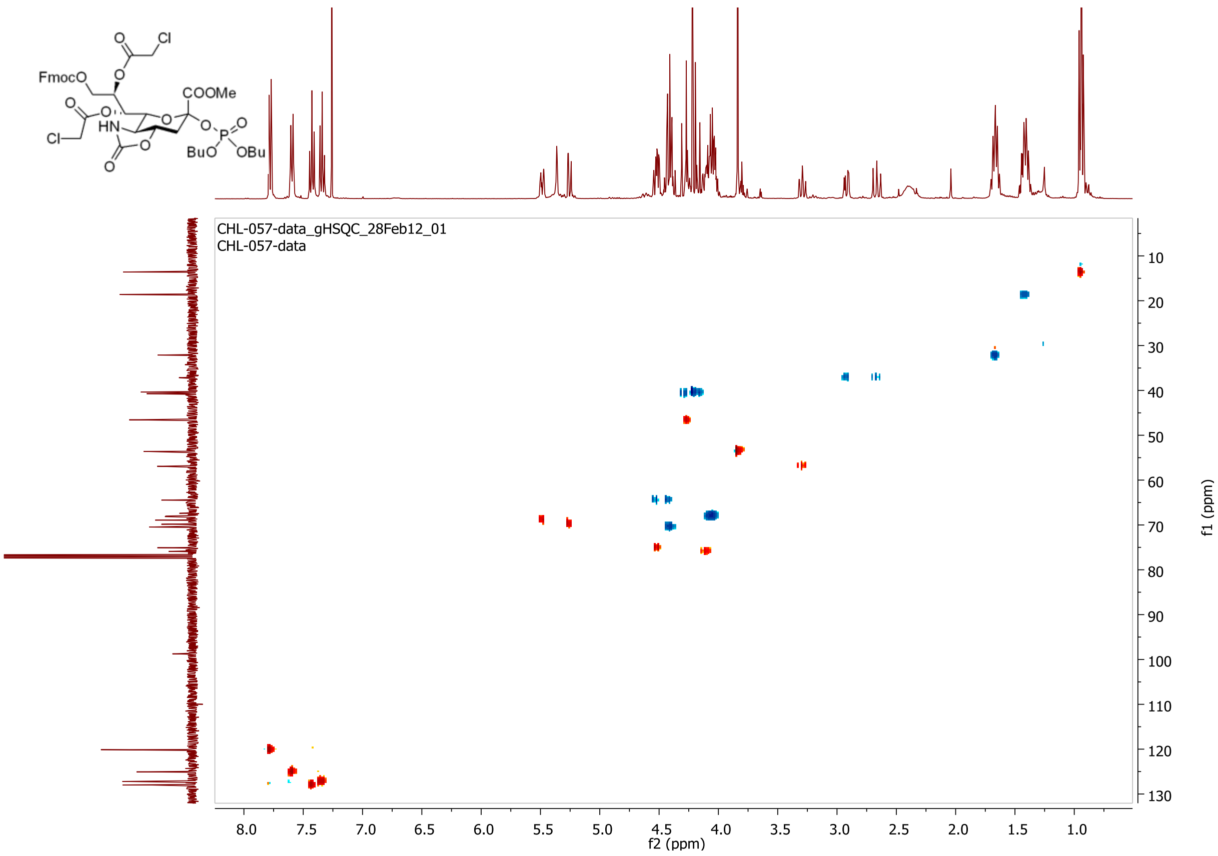
FmocO
COOMe
HN
BuO
OBu
Cl
CHL-057-data_gHSQC_28Feb12_01
CHL-057-data
f2 (ppm)
f1 (ppm)

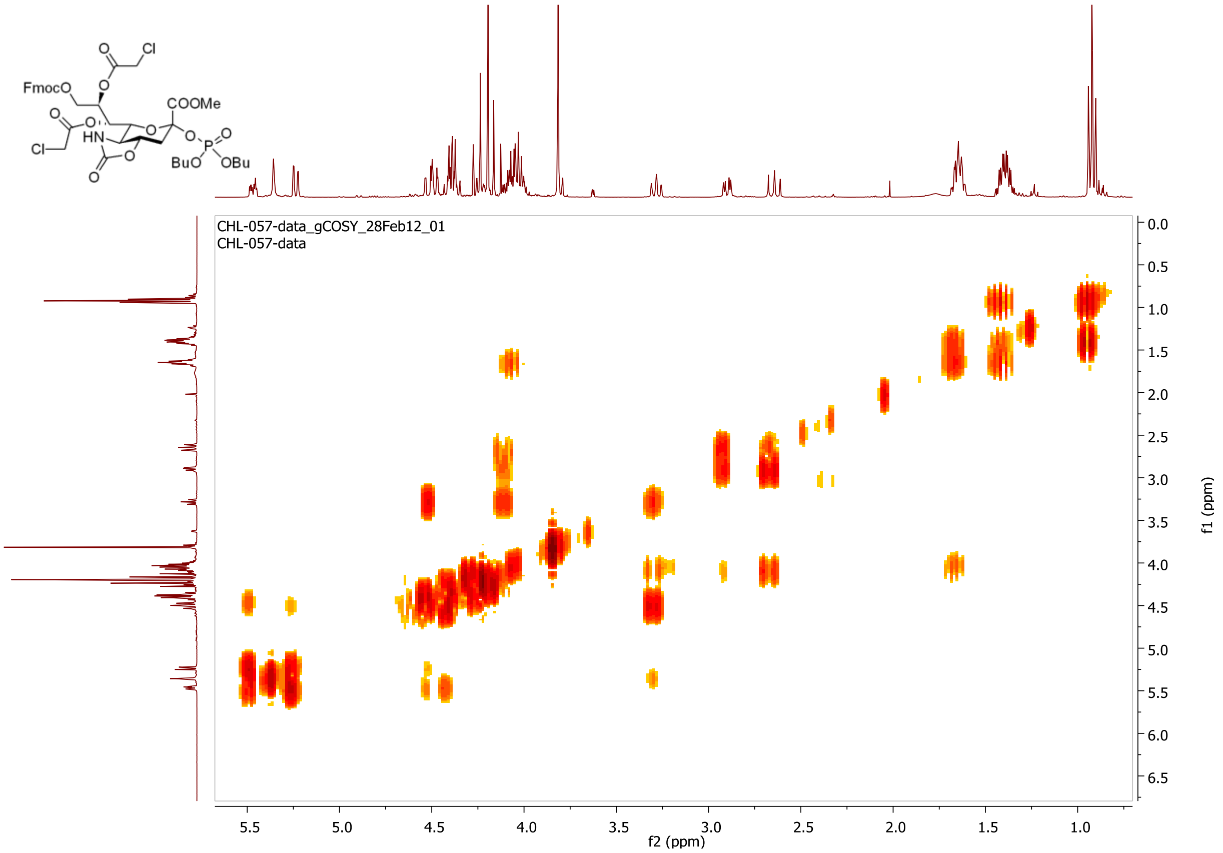

Synthesis Gal thioglycoside building block 6.

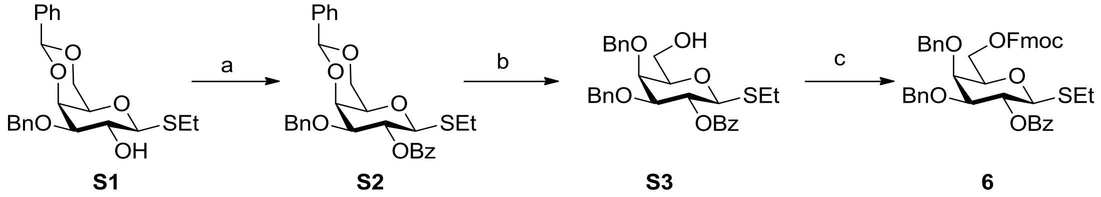

Scheme S1. (a) Bz-Cl, NEt_3, DMAP, CH_2Cl_2, 0 ^{o}C to rt, 18 h, 96%, (b) $BH_3{\cdot}THF$, TMSOTf, THF, 0 ^{o}C, 3 h, 96%, (c) Fmoc-Cl, py, 0 ^{o}C to rt, 18 h, 93%.

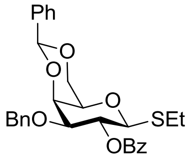

S2

Ethyl 2-*O*-benzoyl-3-*O*-benzyl-4,6-*O*-benzylidene-1-thio-β-D-galactopyranoside (S2)

To a solution of **S1** (4.0 g, 9.94 mmol) in anhydrous CH_2Cl_2 (33 mL, 0.3 M) was added benzoic anhydride (4.5 g, 19.9 mmol), triethylamine (NEt_3, 4.16 mL, 29.8 mmol) and a catalytic amount of DMAP (0.243 g, 1.99 mmol) at 0 °C, the mixture was stirred overnight at

room temperature under Ar. The reaction mixture was quenched with saturated aqueous $NaHCO_3$, and diluted with CH_2Cl_2. The organic layer was dried over $MgSO_4$ and the solvent was evaporated *in vacuo*. The crude was purified by column chromatography on silica gel (Hex/EtOAc = 9:1 to 7:3) to afford **S2** (4.85 g, 9.57 mmol, 96%). R_f = 0.18 (Hex/EtOAc/DCM, 9:1:1); $[\alpha]_D^{20}$ = + 28.0 (c = 2.75, $CHCl_3$); ^{1}H NMR (400 MHz, $CDCl_3$) δ 8.08 – 8.02 (m, 2H), 7.62 – 7.53 (m, 3H), 7.50 – 7.43 (m, 2H), 7.43 – 7.34 (m, 3H), 7.26 – 7.16 (m, 5H), 5.74 (t, *J* = 9.7 Hz, 1H, H-2), 5.52 (s, 1H, C*H*Ph), 4.66 (q, *J* = 12.8 Hz, 2H, CH_2Ph), 4.55 (d, *J* = 9.9 Hz, 1H, H-1), 4.36 (dd, *J* = 12.3, 1.5 Hz, 1H, H-6), 4.28 (dd, *J* = 3.4, 0.8 Hz, 1H, H-4), 4.02 (dd, *J* = 12.4, 1.7 Hz, 1H, H-6), 3.76 (dd, *J* = 9.6, 3.4 Hz, 1H, H-3), 3.47 (d, *J* = 1.1 Hz, 1H, H-5), 2.93 (dq, *J* = 12.3, 7.5 Hz, 1H, C*H*HCH_3), 2.78 (dq, *J* = 12.3, 7.5 Hz, 1H, C*H*HCH_3), 1.28 (t, *J* = 7.5 Hz, 3H). ^{13}C NMR (101 MHz, $CDCl_3$) δ 165.4 (Bz), 138.0, 137.9, 133.1, 130.3, 130.0, 129.2, 128.4, 128.4, 128.3, 127.8, 127.8, 126.6 (Ar), 101.5 (C*H*Ph), 83.0 (C-1), 78.3 (C-3), 73.6 (C-4), 71.1 (CH_2Ph), 70.3 (C-5), 69.5 (C-6), 68.9 (C-2), 22.9, 15.0; IR (thin film): υ = 2871, 1719, 1261 cm^{-1}; HRMS (ESI) calcd. for $C_{29}H_{30}O_6SNa$ $(M+Na)^+$ 529.1661, found 529.1656 m/z.

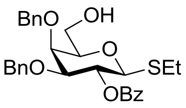

S3

Ethyl 2-*O*-benzoyl-3,4-di-*O*-benzyl-1-thio-β-D-galactopyranoside (S3)

Compound **S2** (6.72 g, 13.26 mmol) was co-evaporated with toluene and dissolved under an Ar atmosphere in CH_2Cl_2 (78 mL, 0.17 M). The solution of compound **S2** was then added 1M solution of BH_3 in THF (66 mL, 66 mmol) and TMSOTf (1.98 mL, 6.63 mmol) at 0 °C under Ar. The mixture was stirred for 3 hr at 0 °C. The mixture was then quenched with saturated aqueous $NaHCO_3$, diluted with CH_2Cl_2. The organic layer was dried over $MgSO_4$ and the solvent was evaporated *in vacuo*. The crude was purified by column chromatography on silica gel (Hex/EtOAc = 9:0.5:0.5 to 9:1:0.5) to afford **S3** (6.5 g, 12.8 mmol, 96%). R_f = 0.27 (Hex/EtOAc, 1:1); $[\alpha]_D^{20}$ = +10.4 (c = 2.10, $CHCl_3$); ^{1}H NMR (400 MHz, $CDCl_3$) δ 8.04 (dd, *J* = 8.2, 1.1 Hz, 2H), 7.59 (ddd, *J* = 7.0, 2.5, 1.3 Hz, 1H), 7.46 (dd, *J* = 10.6, 4.7 Hz, 2H), 7.39 – 7.28 (m, 5H), 7.24 – 7.14 (m, 5H), 5.71 (t, *J* = 9.7 Hz, 1H, H-2), 5.01 (d, *J* = 11.9 Hz, 1H, C*H*HPh), 4.68 (d, *J* = 12.2 Hz, 1H, C*H*HPh), 4.66 (d, *J* = 11.9 Hz, 1H, C*H*HPh), 4.57 (d, *J* = 12.2 Hz, 1H, C*H*HPh), 4.51 (d, *J* = 9.9 Hz, 1H, H-1), 3.94 (d, *J* = 2.6 Hz, 1H, H-4), 3.84 (dd, *J* = 10.4, 6.0 Hz, 1H, H-6), 3.71 (dd, *J* = 9.6, 2.7 Hz, 1H, H-3), 3.54 (dd, *J* = 22.7, 5.6 Hz, 1H, H-6), 3.53 (br, 1H, H-5), 2.82 – 2.64 (m, 2H), 1.22 (t, *J* = 7.5 Hz, 3H). ^{13}C NMR (100 MHz, $CHCl_3$) δ 165.5 (C=O), 138.3, 137.7, 133.2, 130.2, 130.0, 128.6, 128.6, 128.5, 128.5, 128.1,

128.0, 127.9 (Ar), 84.0 (C-1), 81.5 (C-3), 79.2 (C-5), 74.3 (CH_2Ph), 72.4 (C-4), 72.3 (CH_2Ph), 70.4 (C-2), 62.3 (C-6), 23. 9, 15. 0.; IR (thin film): υ = 2871, 1723, 1453, 1268 cm^{-1}; HRMS (ESI) calcd. for $C_{29}H_{32}O_6SNa$ $(M+Na)^+$ 531.1817, found 531.1832 m/z.

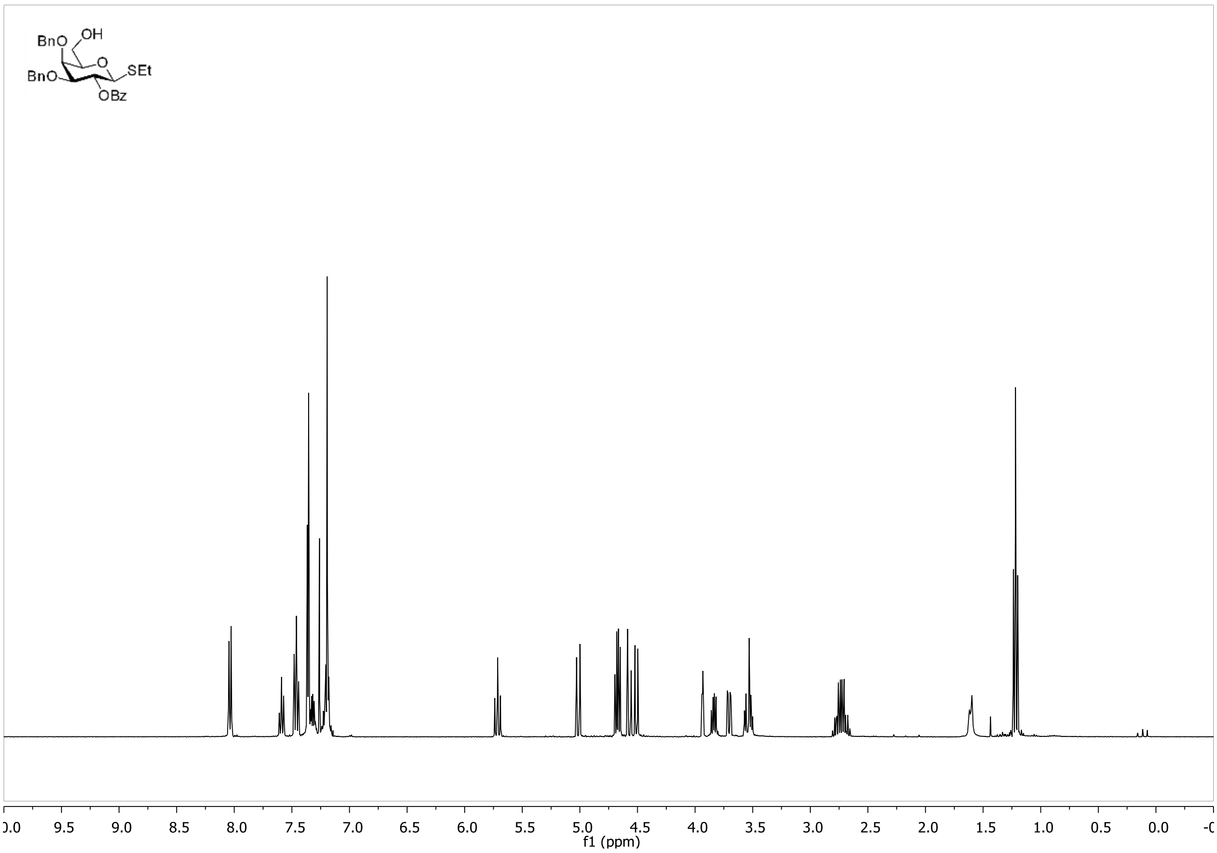

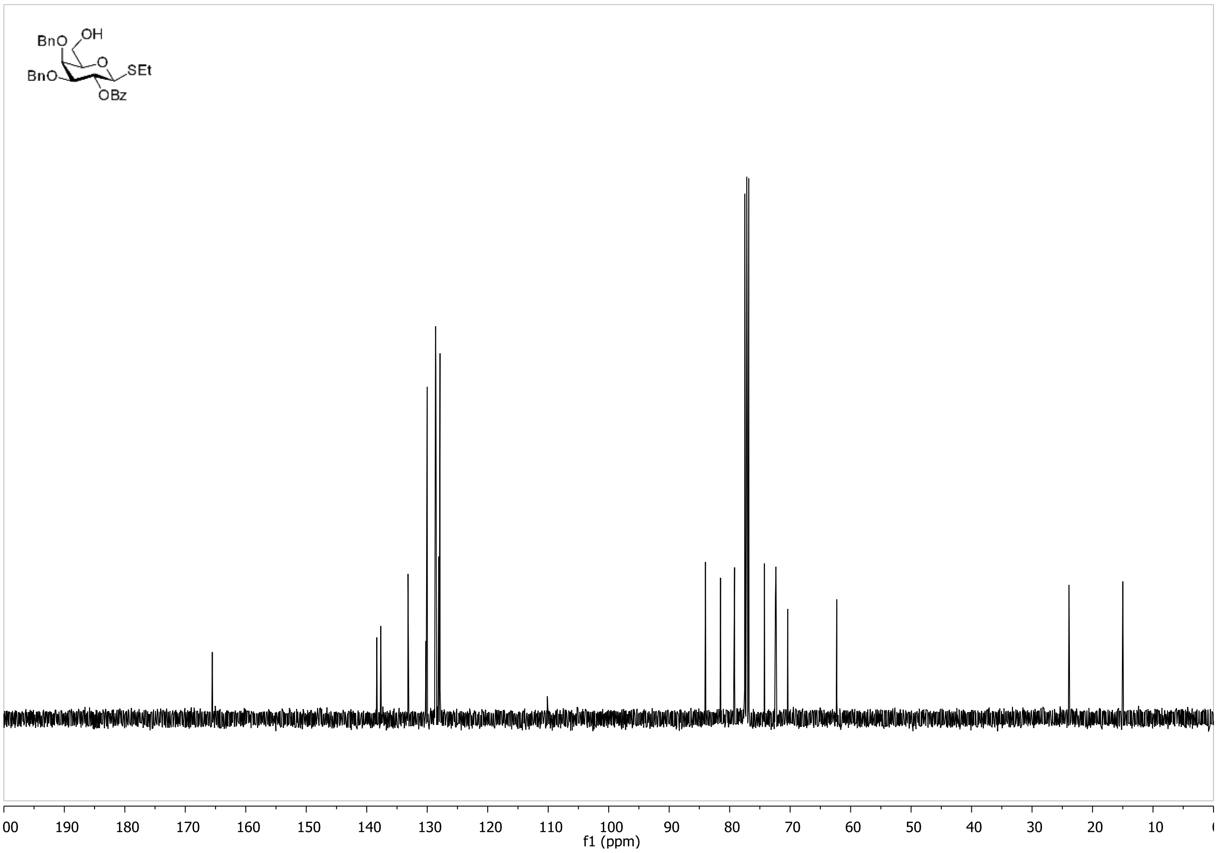

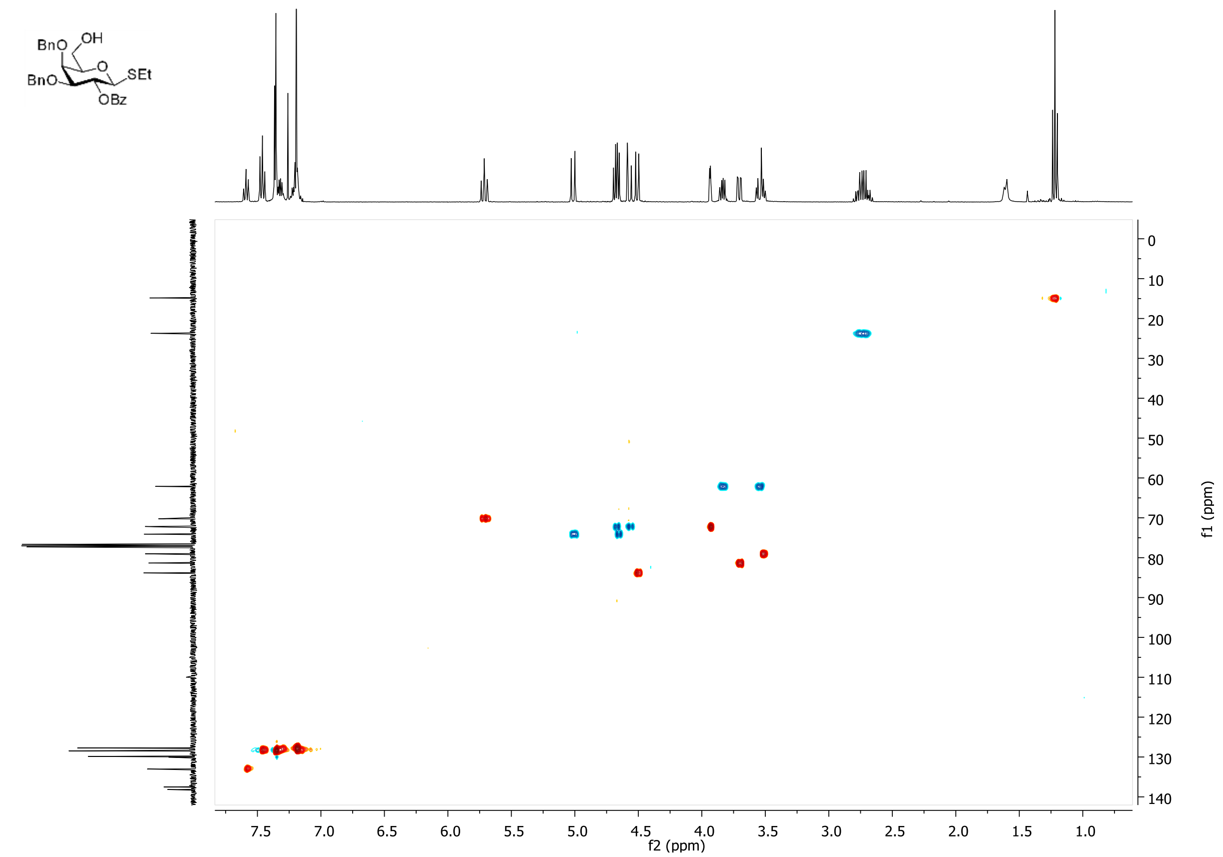
BnO
OH
O
SEt
BnO
OBz
f2 (ppm)
f1 (ppm)

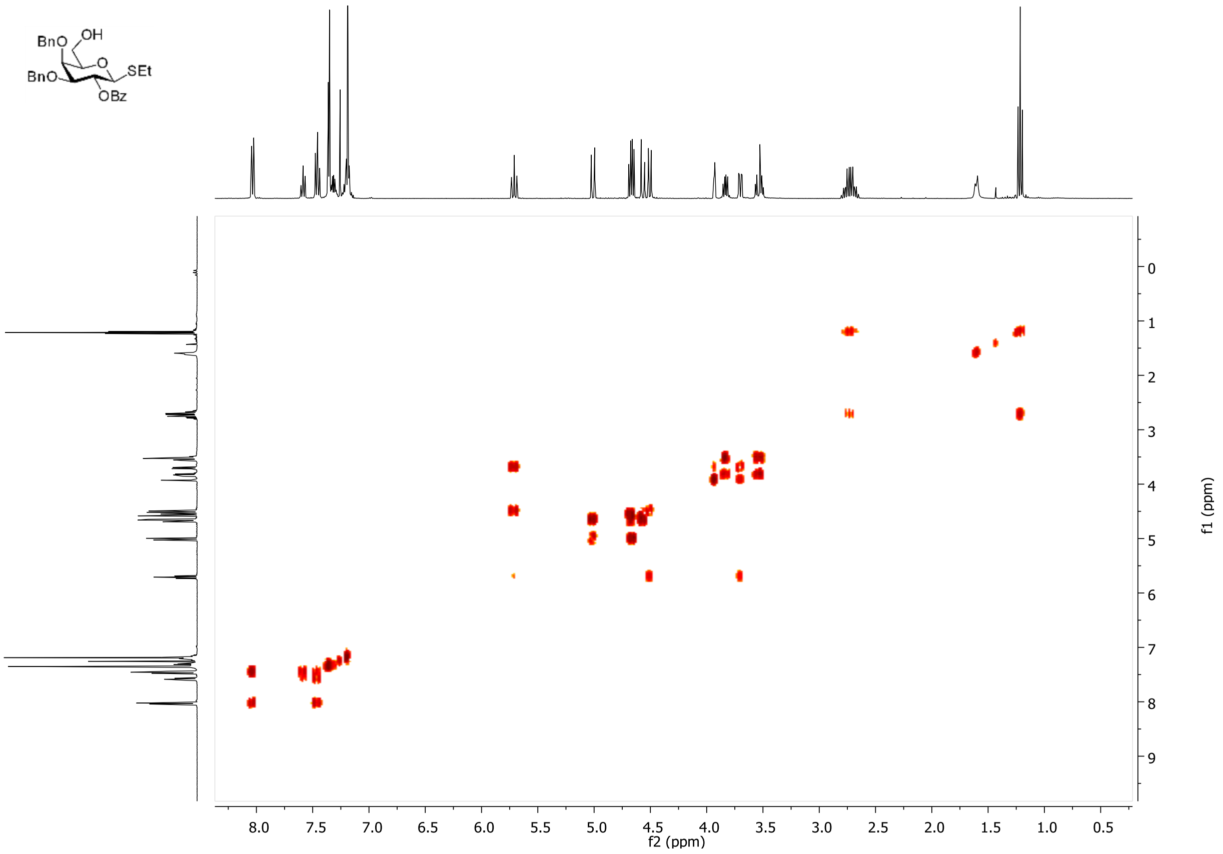
BnO
OH
O
SEt
BnO
OBz
f2 (ppm)
f1 (ppm)

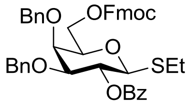

6

Ethyl 2-*O*-benzoyl-3,4-di-*O*-benzyl-6-*O*-fluorenylmethoxycarbonyl-1-thio-β-D-galacto-pyranoside (6)

To a solution of compound **S3** (4.0 g, 7.86 mmol) in anhydrous CH_2Cl_2 (26 mL) was added FmocCl (4.07 g, 15.73 mmol) and pyridine (1.91 mL, 23.6 mmol) at 0 °C, and then was stirred overnight at room temperature. The reaction mixture was quenched with 1M aqueous HCl, and diluted with DCM. The organic layer was dried over $MgSO_4$ and the solvent was evaporated *in vacuo*. The crude was purified by column chromatography on silica gel (Hex/EtOAc/DCM = 8/1/1 to 8/2/1) to afford **6** (5.35 g, 7.32 mmol, 93%). R_f = 0.27 (Hex/EtOAc/DCM, 9:1:0.5); $[\alpha]_D^{20}$ = +22.4 (c = 2.50, $CHCl_3$); ^{1}H NMR ($CDCl_3$) 8.03 (d, *J* = 7.5 Hz, 2H), 7.78 (d, *J* = 7.5 Hz, 2H), 7.59 (t, *J* = 7.7 Hz, 3H), 7.49 – 7.39 (m, 4H), 7.38 – 7.26 (m, 7H), 7.23 – 7.14 (m, 5H), 5.71 (t, *J* = 9.7 Hz, 1H, H-2), 5.04 (d, *J* = 11.7 Hz, 1H, C*H*HPh), 4.68 (d, *J* = 12.2 Hz, 1H, C*H*HPh), 4.67 (d, *J* = 11.7 Hz, 1H, C*H*HPh), 4.56 (d, *J* = 12.2 Hz, 1H, C*H*HPh), 4.52 (d, *J* = 9.9 Hz, 1H, 4.44 – 4.34 (m, 3H, H-6, CH_2-Fmoc), 4.25 (t, *J* = 7.4 Hz, 1H, C*H*-Fmoc), 4.18 (dd, *J* = 11.1, 5.8 Hz, 1H, H-6), 3.97 (d, *J* = 1.9 Hz, 1H, H-4), 3.72 (dd, *J* = 13.2, 4.1 Hz, 2H, H-3, H-5), 2.82 – 2.64 (m, 2H), 1.22 (t, *J* = 7.4 Hz, 3H). ^{13}C NMR (101 MHz, $CHCl_3$) δ 165.5 (C=O, Bz), 154.9 (C=O, Fmoc), 143.4, 143.4, 141.4, 138.1, 137.6, 133.2, 130.1, 130.0, 128.5, 128.5, 128.4, 128.1, 127.9, 127.9, 127.8, 127.3, 125.2, 120.2 (Ar), 84.0 (C-1), 81.2 (C-3), 76.2 (C-5), 74.5 (CH_2Ph), 72.6 (C-4), 72.3 (CH_2Ph), 70.2 (*C*H_2-Fmoc), 70.1 (C-2), 66.8 (C-6), 46.8 (*C*H- Fmoc), 24.1, 15.0; IR (thin film): υ = 2871, 1748, 1727, 1451 cm^{-1}; HRMS (ESI) calcd. for $C_{44}H_{42}O_8SNa$ $(M+Na)^+$ 753.2498, found 753.2477.

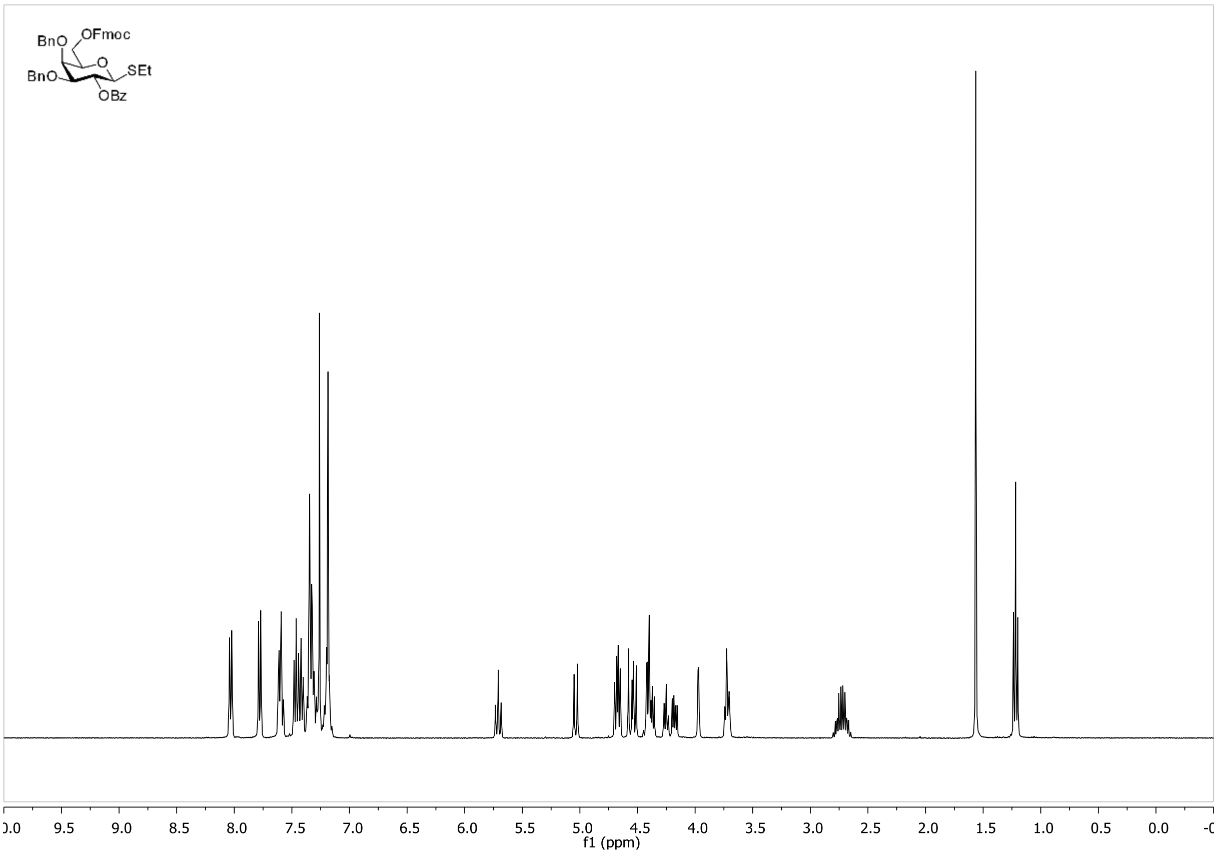
BnO
OFmoc
O
SEt
BnO
OBz
f1 (ppm)

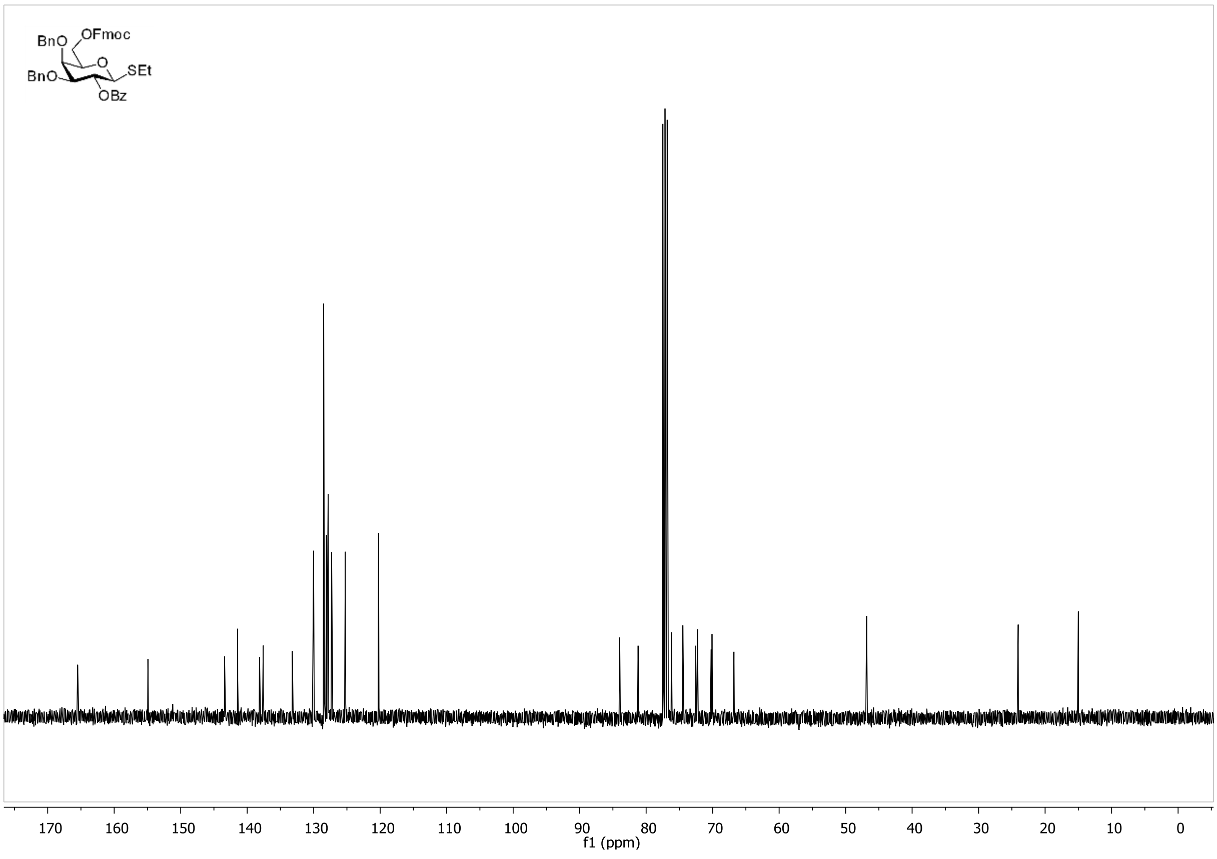
BnO
OFmoc
O
SEt
BnO
OBz
f1 (ppm)

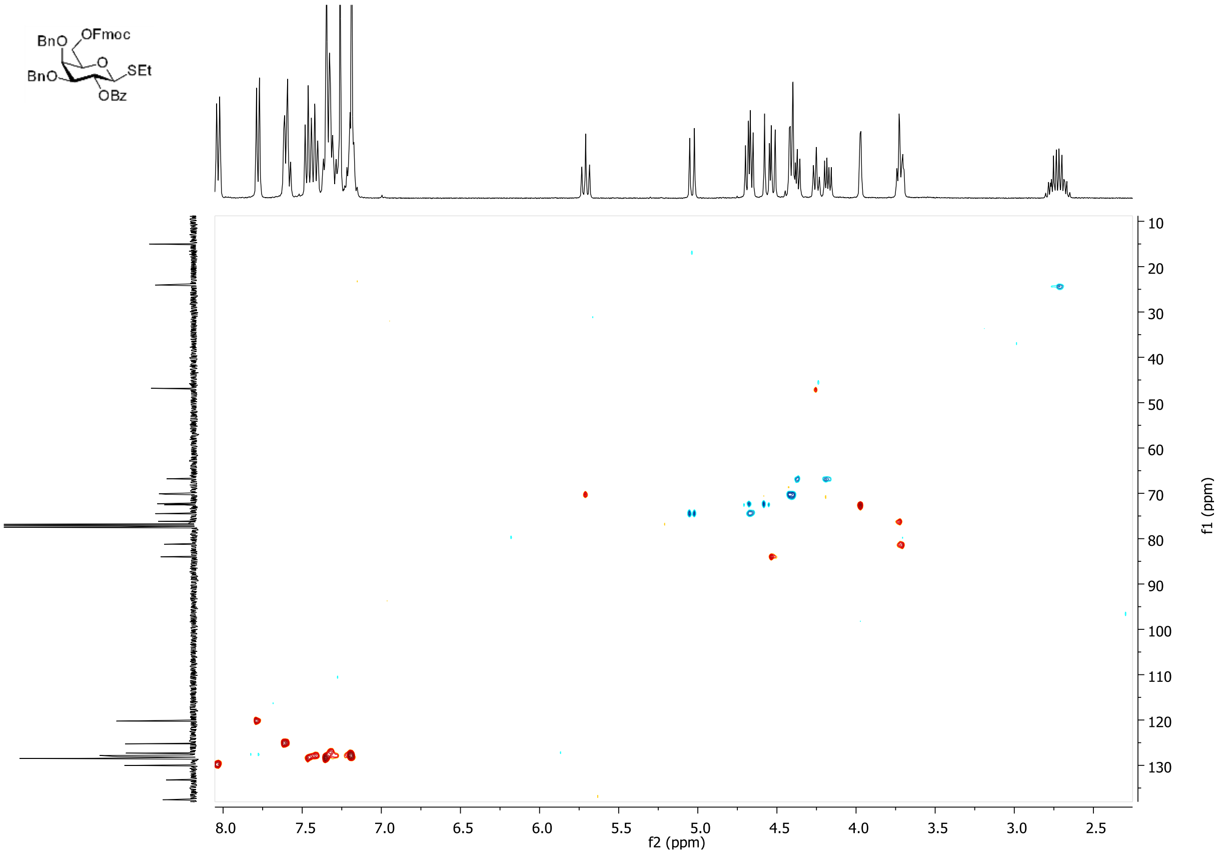
BnO
OFmoc
O
SEt
BnO
OBz
f2 (ppm)
f1 (ppm)

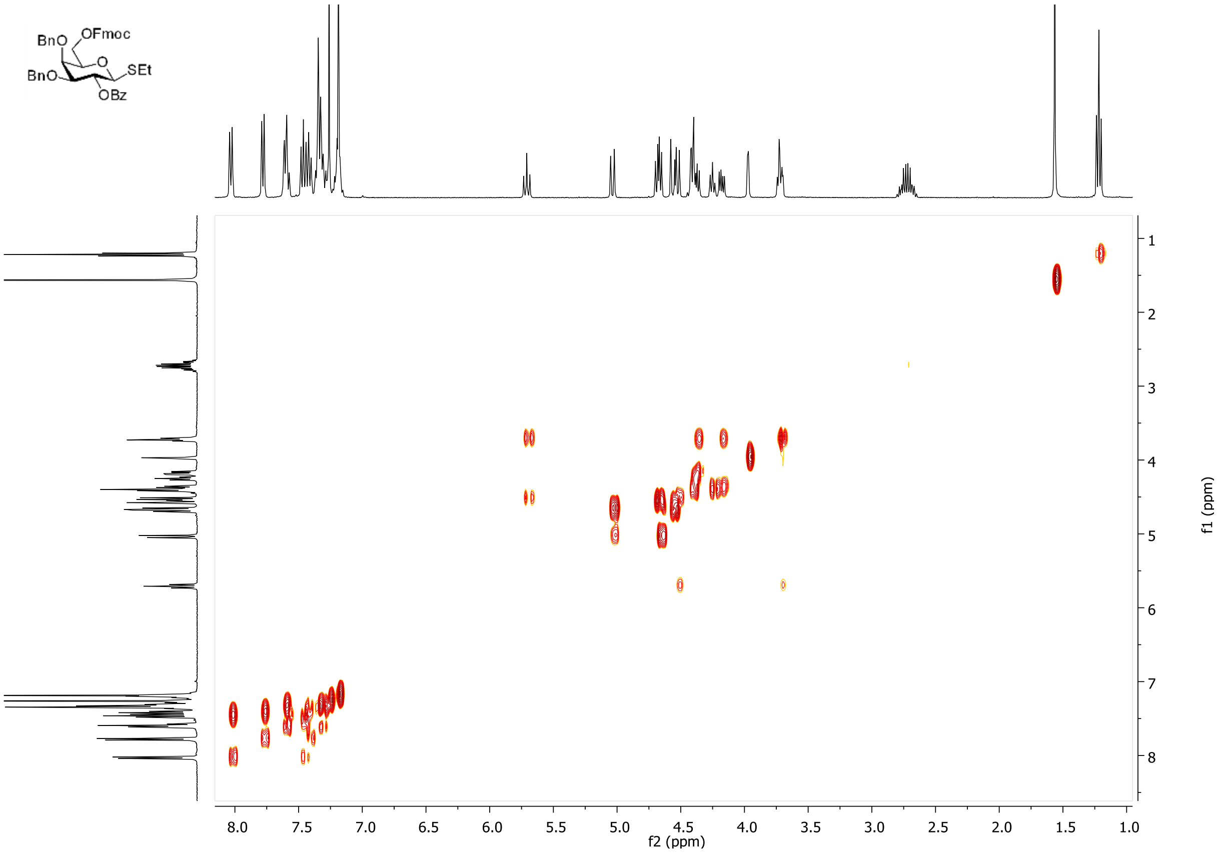
BnO
OFmoc
O
SEt
BnO
OBz
f2 (ppm)
f1 (ppm)

Synthesis of GalN phosphate building block 8.

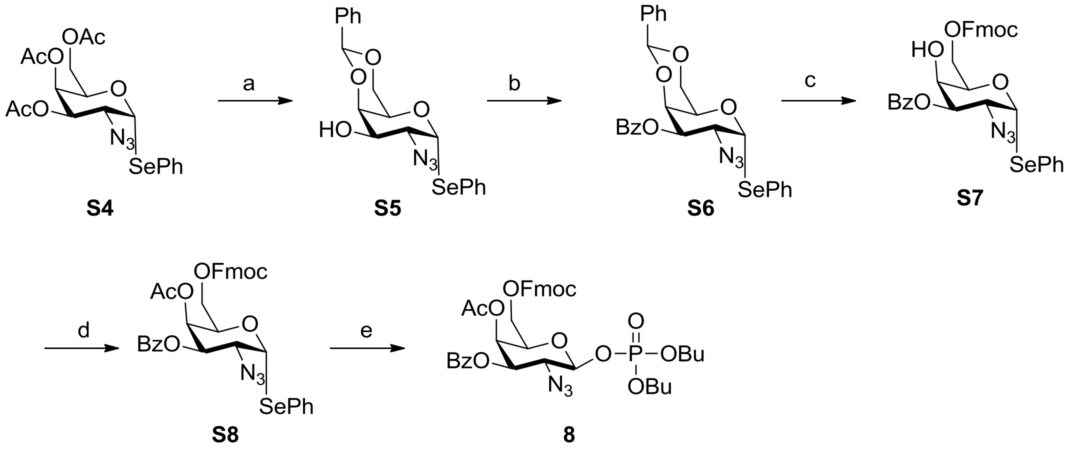

Scheme S1. (a) 1. NaOMe, MeOH, rt, 4 h; 2. PhCH(OMe)$_2$, CAS, CH_3CN, rt, 2.5 h, 88%, (b) BzCl, py, CH_2Cl_2, rt, 18 h, 99%, (c) 1. TFA, CH_2Cl_2/ H_2O, 0 ^{o}C, 2 h; 2. FmocCl, py, CH_2Cl_2, rt, 18 h, 76%, (d) AcCl, py, 0 ^{o}C, 2h, 92%, (e) HOPO(OBu)$_2$, NIS, 4A MS, CH_2Cl_2, -15 ^{o}C, 2 h, 85%

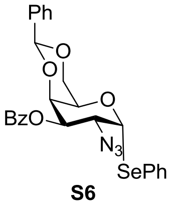

S6

Phenyl 2-deoxy-2-azido-3-*O*-benzoyl-4,6-*O*-benzylidene-1-seleno-α-D-galactopyranoside (S6)

To a solution of **S4**[3] (9.85 g, 20.94 mmol) in anhydrous methanol (160 mL) at room temperature was added a solution of NaOMe in MeOH (2.10 mL, 0.5 M, 1.05 mmol). The reaction was stirred for 4 h and then neutralized with Amberlite 120 (H^+) resin. The resin was filtered off and the solvent was removed in *vacuo* to obtain the deacylated compound as a pale yellow solid. The crude product was used without further purification for the next step.

To the above triol in CH_3CN (102 mL) was added benzaldehyde dimethyl acetal (9.43 mL, 62.80 mmol) and camphorsulfonic acid (CSA, 0.049 g, 0.21 mmol) and stirred at room temperature for 18 h. After complete conversion of the starting material, the reaction was quenched by the addition of triethylamine (1 mL) and the volatiles were removed in *vacuo.* The crude was purified by flash chromatography (Hex/EtOAc, form 10/1 to 4/1) to obtain the compound as a white solid **S5** (8.0 g, 88%). R_f = 0.17 (Hex/EtOAc, 4/1)

To a solution of **S5** (3 g, 6,94 mmol) in anhydrous CH_2Cl_2 (50 mL) was added benzoic anhydride (1.61 mL, 13.88 mmol), pyridine (2.25 mL, 27.80 mmol) and DMAP (0.085 g, 0.69

mmol) at room temperature for 18 h. After completed conversion of the starting material the solution was diluted with CH_2Cl_2 and extracted with 1 M aqueous HCl and saturated aqueous $NaHCO_3$. The organic phase was dried over $MgSO_4$ and the solvent was removed in *vacuo*. The crude product was purified by silica gel flash column chromatography column chromatography (Hex/EtOAc, form 10/1 to 2/1) to afford **S6** (3.68 g, 99%). R_f = 0.32 (Hex/ EtOAc = 6/1); $[\alpha]_D^{20}$ = + 350.1 (c = 1.0, CH_2Cl_2); ^1H NMR (400 MHz, $CDCl_3$) δ 4.08 (dd, J = 12.6, 1.7 Hz, 1H), 4.16 (dd, J = 12.7, 1.6 Hz, 1H), 4.24 (m, 1H), 4.66 (d, J = 3.4 Hz, 1H), 4.67 (dd, J = 10.8, 5.2 Hz, 1H), 5.30 (dd, J = 10.8, 3.4 Hz, 1H), 5.54 (s 1H), 6.14 (d, J = 5.2 Hz, 1H), 7.29 – 7.25 (m, 3H), 7.35 – 7.30 (m, 3H), 7.49 – 7.40 (m, 4H), 7.63 – 7.55 (m, 3H), 8.08 (dd, J = 8.3, 1.3 Hz, 2H); ^{13}C NMR (101 MHz, $CDCl_3$) δ 58.9, 64.9, 69.0, 72.9, 73.1, 85.1 (C-1), 100.6, 126.0, 127.9, 128.2, 128.4, 128.6, 129.0, 129.1, 129.2, 130.0, 133.6, 134.0, 137.4, 165.6; IR (thin film) 3060, 2922, 2855, 2248, 2109, 1719, 1601, 1578, 1492, 1477, 1451, 1438, 1401, 1366, 1338, 1314, 1271, 1251, 1214, 1177, 1161, 1087, 1069, 1057, 1023, 999, 987, 920, 894, 847, 803, 771, 737, 710, 696, 671, 649 cm^{-1}; HRMS (ESI) calcd. for $C_{26}H_{23}N_3O_5SeNa$ $(M+Na)^+$ 560.0701, found 560.0733.

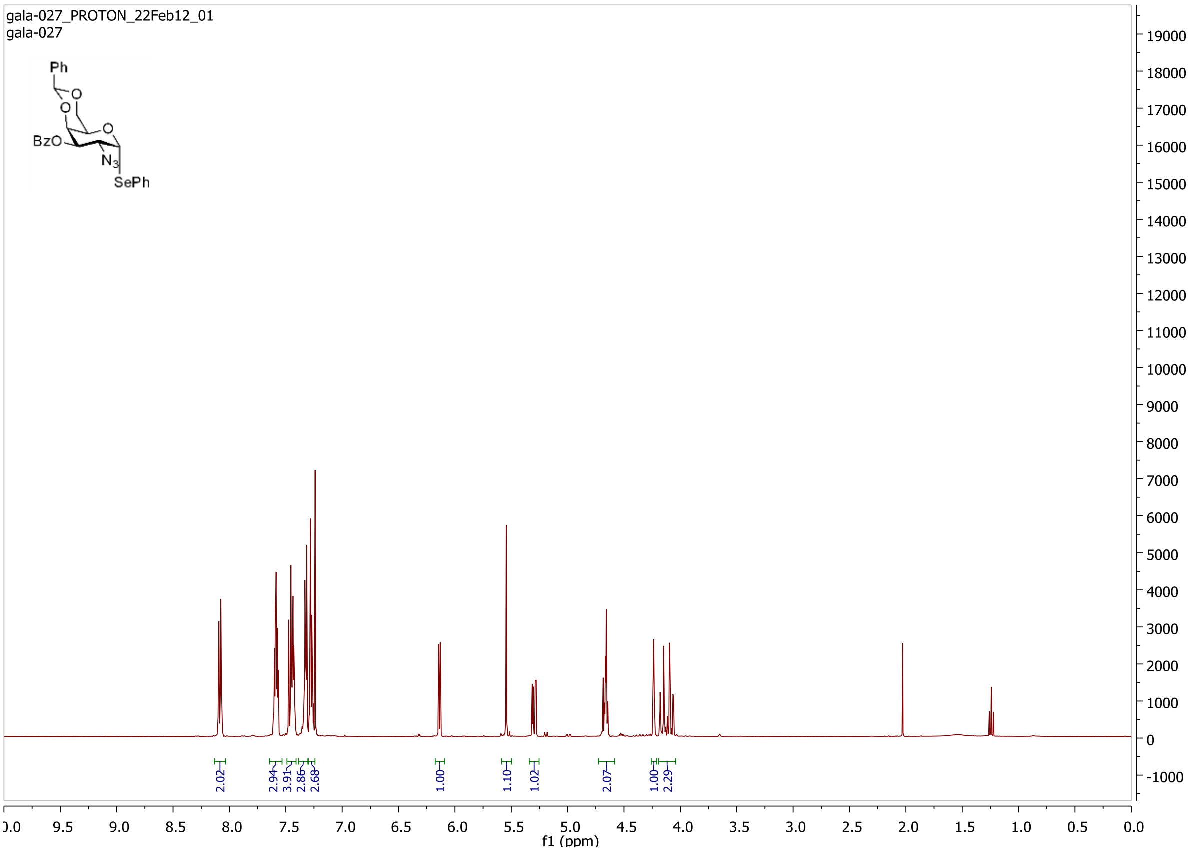

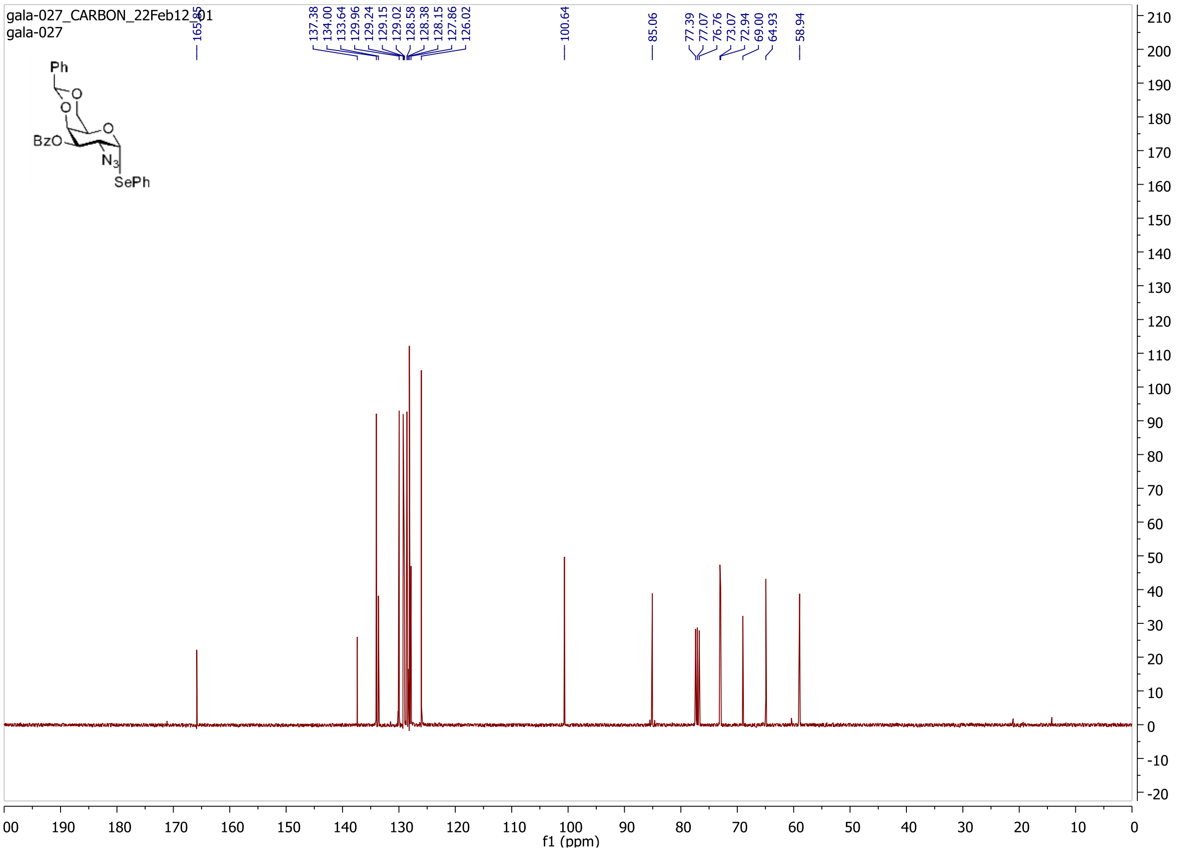
gala-027_CARBON_22Feb12_01
gala-027
165.85
137.38
134.00
133.64
129.96
129.24
129.15
129.02
128.58
128.38
128.15
127.86
126.02
100.64
85.06
77.39
77.07
76.76
73.07
72.94
69.00
64.93
58.94
Ph
O
O
O
BzO
N3
SePh
f1 (ppm)

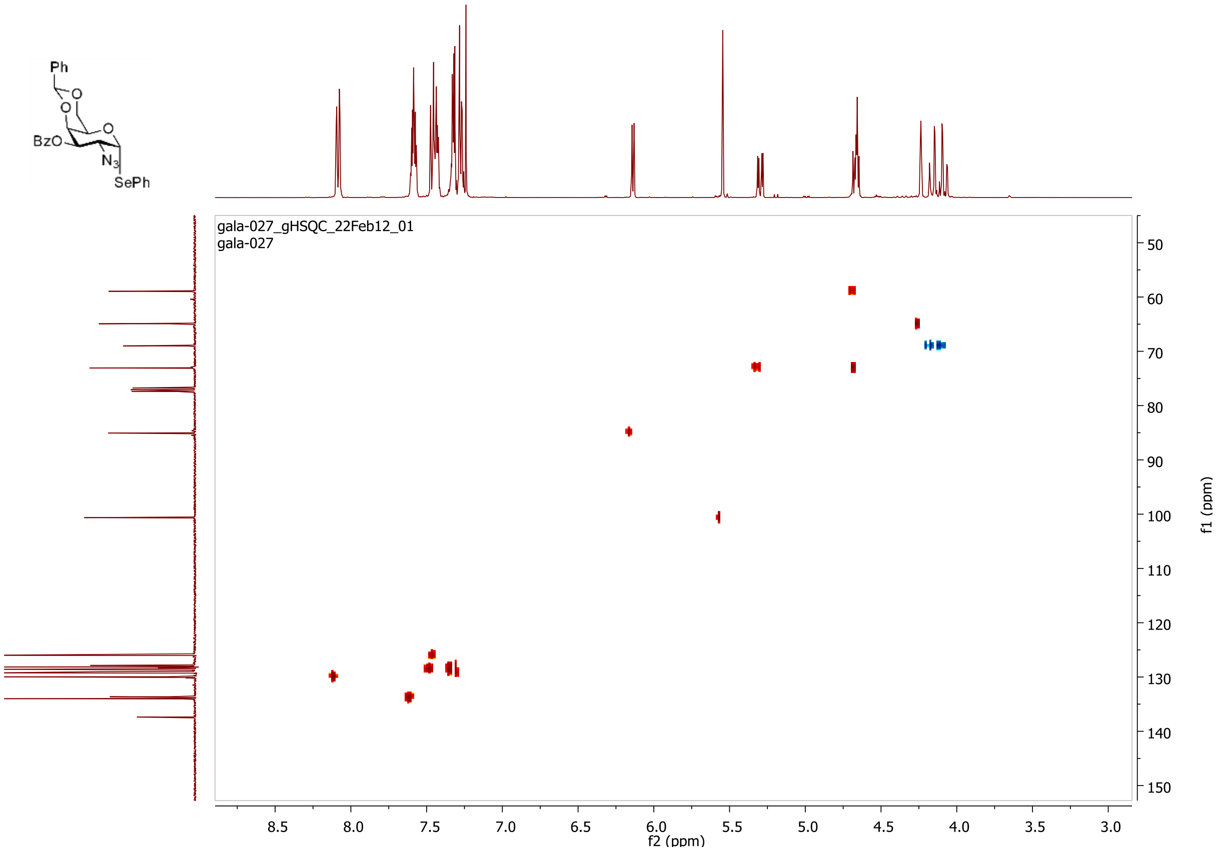
Ph
O
O
O
BzO
N3
SePh
gala-027_gHSQC_22Feb12_01
gala-027
f2 (ppm)
f1 (ppm)

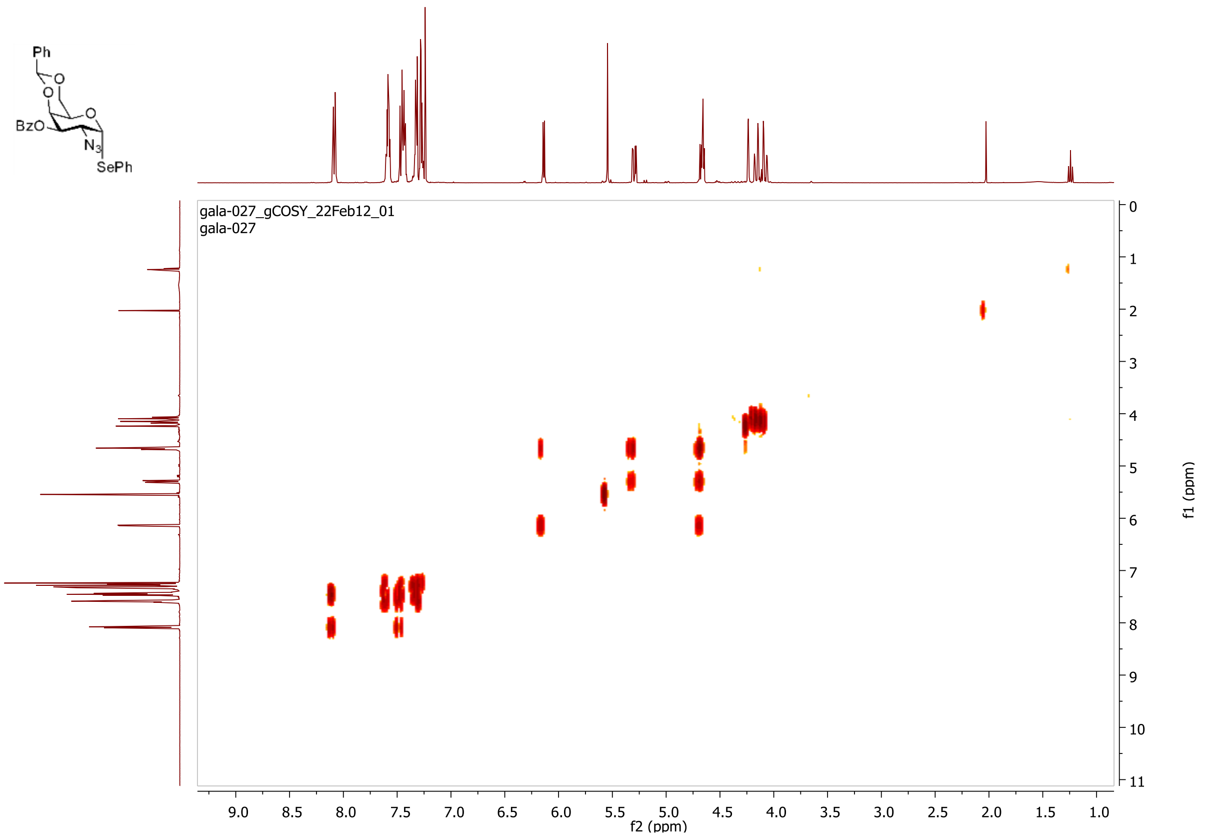

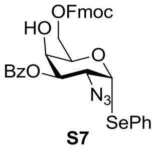

S7

Phenyl 2-deoxy-2-azido-3-*O*-benzoyl-6-*O*-fluorenylmethoxycarbonyl-1-seleno-α-D-galactopyranoside (S7)

To a soultion of **S6** (1.0 g, 1.86 mmol) in CH_2Cl_2 (16 mL) and H_2O (1 mL) at 0 ^oC for 15 min, TFA (2 mL) was added into reaction mixture for 2 h. After complete conversion of the starting material, the reaction mixture was diluted with CH_2Cl_2 and then saturated aqueous $NaHCO_3$ was added to quench the reaction at 0 ^oC. The organic phase was wash with brine, dried over $MgSO_4$ and then concentrated. The crude product was used without further purification for the next step. R_f = 0.31 (Hex/EtOAc = 2/1).

To a solution of above compound in anhydrous CH_2Cl_2 (19 mL) was added Fmoc-Cl (0.482 g, 1.86 mmol) and pyridine (0.45 ml, 5.59 mmol) at 0 °C, and then was stirred overnight at room temperature under Ar. The reaction mixture was diluted with CH_2Cl_2 and washed with brine, dried over $MgSO_4$ and then concentrated. The residue was purified by silica gel column chromatography (Hex/ EtOAc, form 100/1 to 4/1) to give **S7** (950 mg, 76% for two steps). R_f = 0.66 (Hex/EtOAc = 2/1); $[\alpha]_D^{20}$ = + 217.7 (c = 1.0, $CHCl_3$); ^1H NMR (400 MHz, $CDCl_3$)

δ 8.15 – 8.04 (m, 2H), 7.78 (dd, *J* = 7.4, 3.5 Hz, 2H), 7.70 – 7.62 (m, 2H), 7.58 (dd, *J* = 9.9, 4.2 Hz, 3H), 7.50 – 7.38 (m, 4H), 7.35 – 7.29 (m, 2H), 7.28 – 7.21 (m, 3H), 6.01 (d, *J* = 5.5 Hz, 1H), 5.25 (dd, *J* = 10.8, 2.9 Hz, 1H), 4.68 (t, *J* = 6.3 Hz, 1H), 4.56 (dd, *J* = 10.8, 5.5 Hz, 1H), 4.43 – 4.34 (m, 4H), 4.30 (dd, *J* = 11.5, 6.3 Hz, 1H), 4.22 (t, *J* = 7.1 Hz, 1H), 2.62 (s, 1H). ^{13}C NMR (101 MHz, $CDCl_3$) δ 165.4, 155.1, 143.1, 143.1, 141.3, 141.2, 135.1, 133.8, 129.8, 129.2, 128.8, 128.6, 128.2, 127.9, 127.9, 127.5, 127.2, 127.2, 125.0, 120.1, 120.1, 84.6 (C-1), 77.3, 77.0, 76.7, 74.0, 70.0, 66.7, 65.9, 58.9, 46.6. IR (thin film) 3484, 3063, 2952, 2113, 1725, 1601, 1580, 1478, 1451, 1268, 1085, 1071 cm^{-1}; HRMS (ESI) calcd. for $C_{34}H_{29}N_3O_7SeNa$ $(M+Na)^+$ 649.1068, found 649.1079.

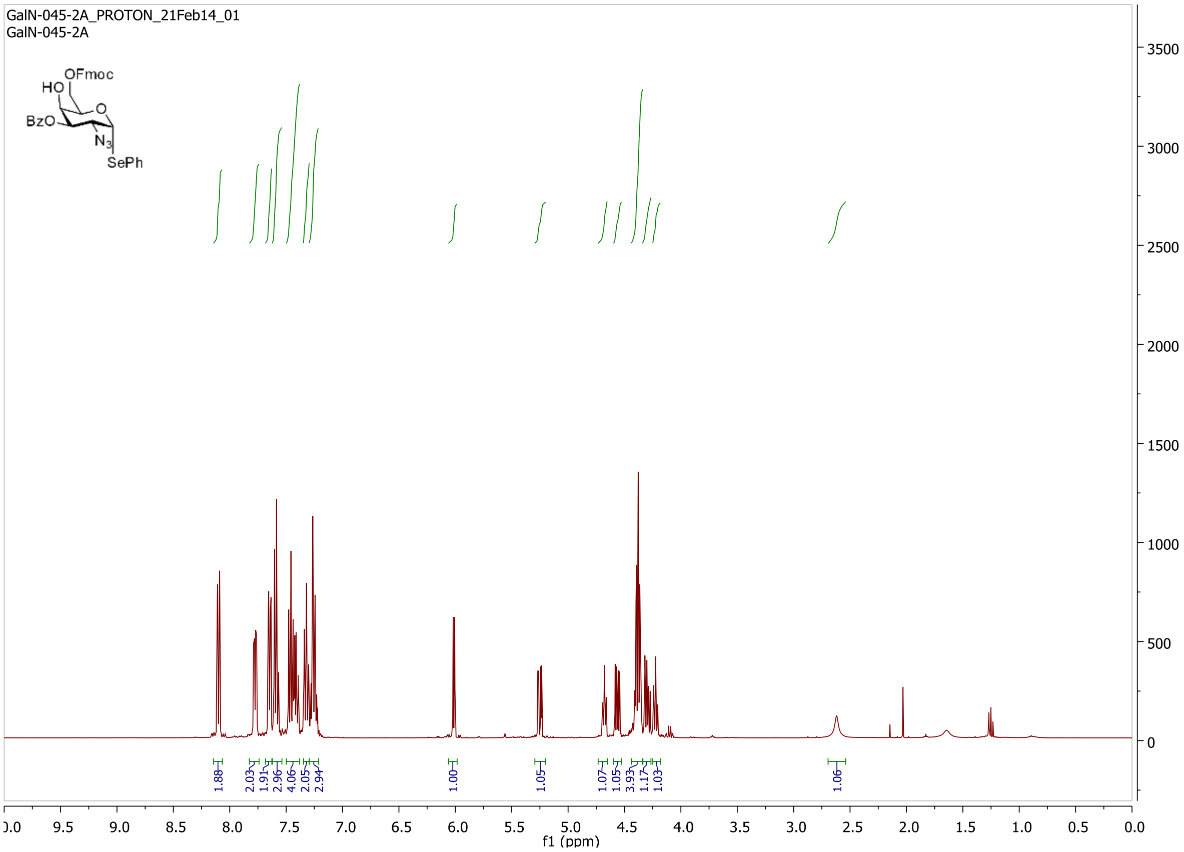

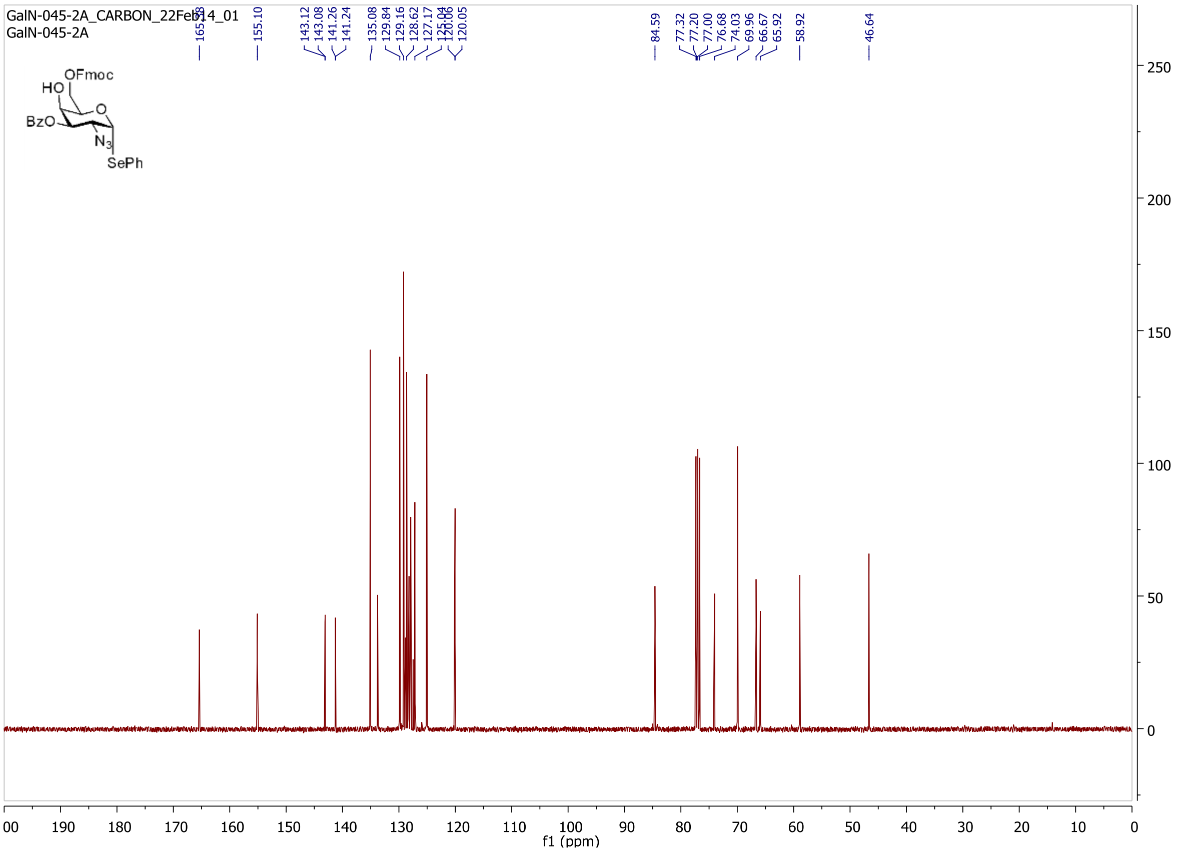
GalN-045-2A_CARBON_22Feb14_01
GalN-045-2A
155.10
143.12
143.08
141.26
141.24
135.08
129.84
129.16
128.62
127.17
125.04
120.06
120.05
84.59
77.32
77.20
77.00
76.68
74.03
69.96
66.67
65.92
58.92
46.64
OFmoc
HO
BzO
O
N3
SePh
f1 (ppm)

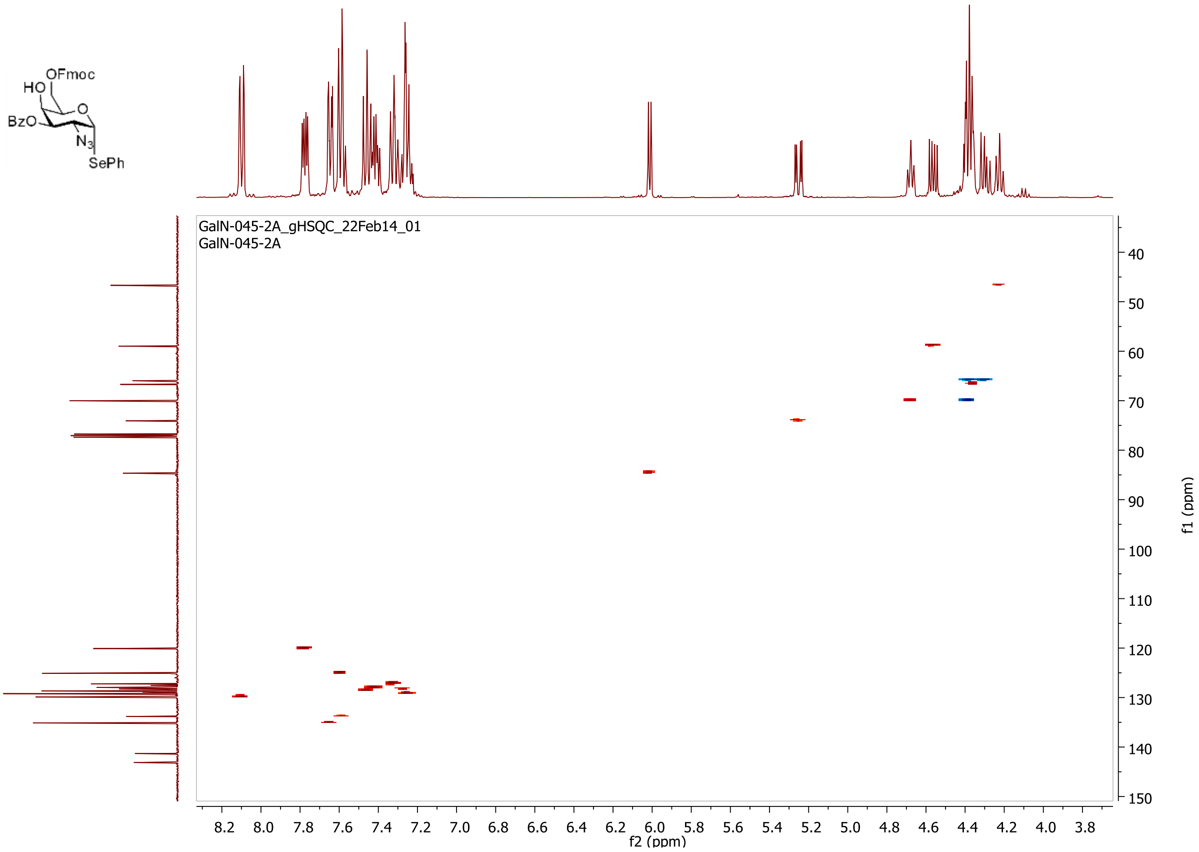
OFmoc
HO
BzO
O
N3
SePh
GalN-045-2A_gHSQC_22Feb14_01
GalN-045-2A
f2 (ppm)
f1 (ppm)

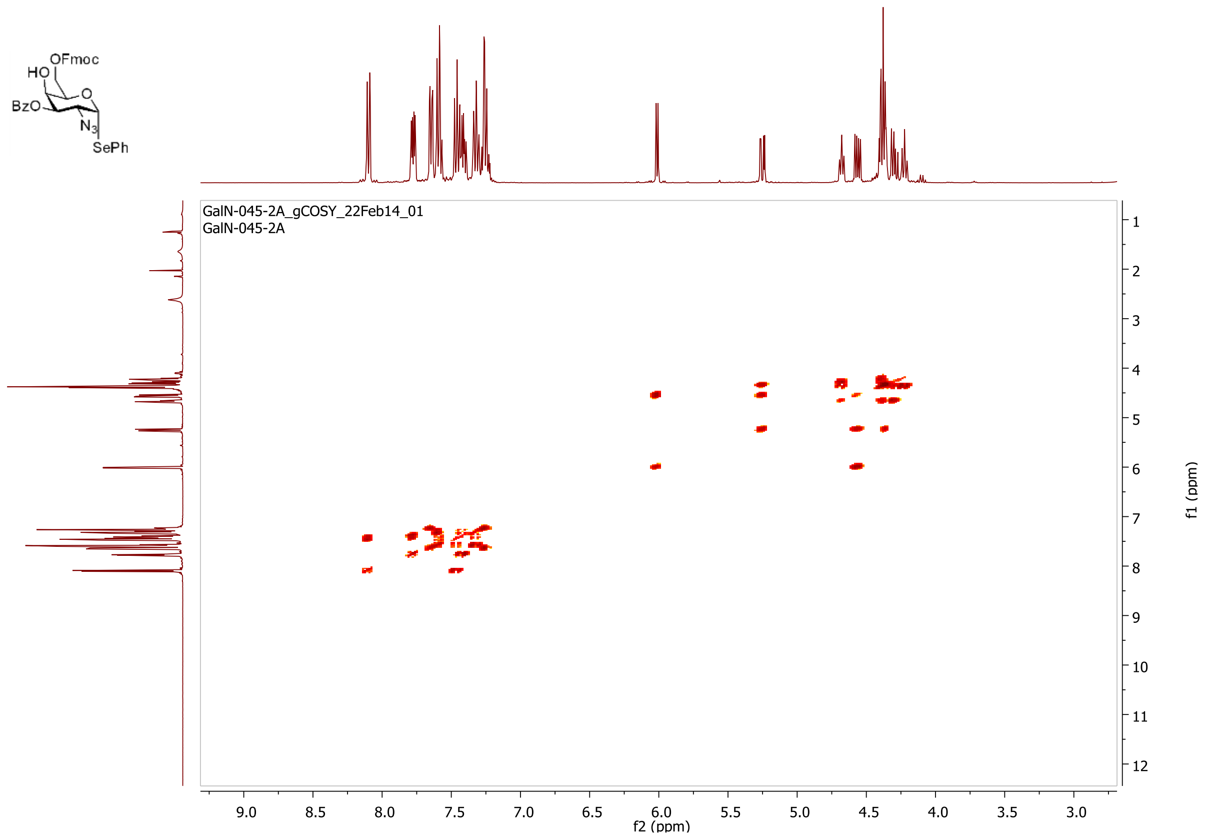

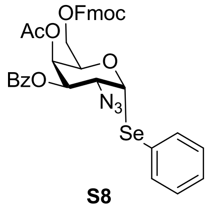

S8

Phenyl 2-deoxy-2-azido-3-*O*-benzoyl-4-*O*-acetyl-6-*O*-fluorenylmethoxycarbonyl-1-seleno-α-D-galactopyranoside (S8)

To a solution of **S7** (0.50 g, 0.75 mmol) in CH_2Cl_2 (7.46 mL) at 0 ^{o}C for 10 min, acetyl chloride (0.064 mL, 0.90 mmol) and pyridine (0.30 mL, 3.73 mmol) were added for 2 h under Ar. After complete conversion of the starting material, the reaction mixture was diluted with CH_2Cl_2 and extracted with water, 1 N HCl solution. The combined extracts were washed with brine, dried over $MgSO_4$ and then concentrated. The residue was purified by silica gel column chromatography (Hex/ EtOAc, form 50 /1 to 4 /1) to give **S8** (490 mg, 92%). R_f = 0.30 (Hex/EtOAc, 5/1); $[\alpha]_D^{20}$ = + 207.2 (c = 1.0, $CHCl_3$); ^{1}H NMR (400 MHz, $CDCl_3$) δ 8.04 – 7.95 (m, 2H), 7.78 (d, J = 7.6 Hz, 2H), 7.70 – 7.53 (m, 5H), 7.44 (m, 4H), 7.36 – 7.26 (m, 3H), 7.25 – 7.18 (m, 2H), 6.05 (d, J = 5.5 Hz, 1H), 5.73 (d, J = 3.1 Hz, 1H), 5.36 (dd, J = 11.0, 3.1 Hz, 1H), 4.86 (t, J = 6.1 Hz, 1H), 4.54 – 4.31 (m, 3H), 4.33 – 4.11 (m, 3H), 2.10 (s, 3H). ^{13}C

NMR (101 MHz, $CDCl_3$)δ 169.6, 165.1, 154.7, 143.2, 143.1, 141.3, 141.3, 135.2, 133.7, 129.7, 129.3, 128.9, 128.6, 128.4, 127.9, 127.3, 127.2, 127.2, 125.2, 125.1, 120.1, 84.4, 71.7, 70.1, 69.1, 67.3, 65.4, 59.3, 46.7, 20.5. IR (thin film) 3062. 2956, 2111, 1751, 1602, 1477, 1451, 1375, 1263, 1218, 1105 cm^{-1}; HRMS (ESI) calcd. for $C_{36}H_{31}N_3O_8SeNa$ $(M+Na)^+$ 736.1174, found 736.1196.

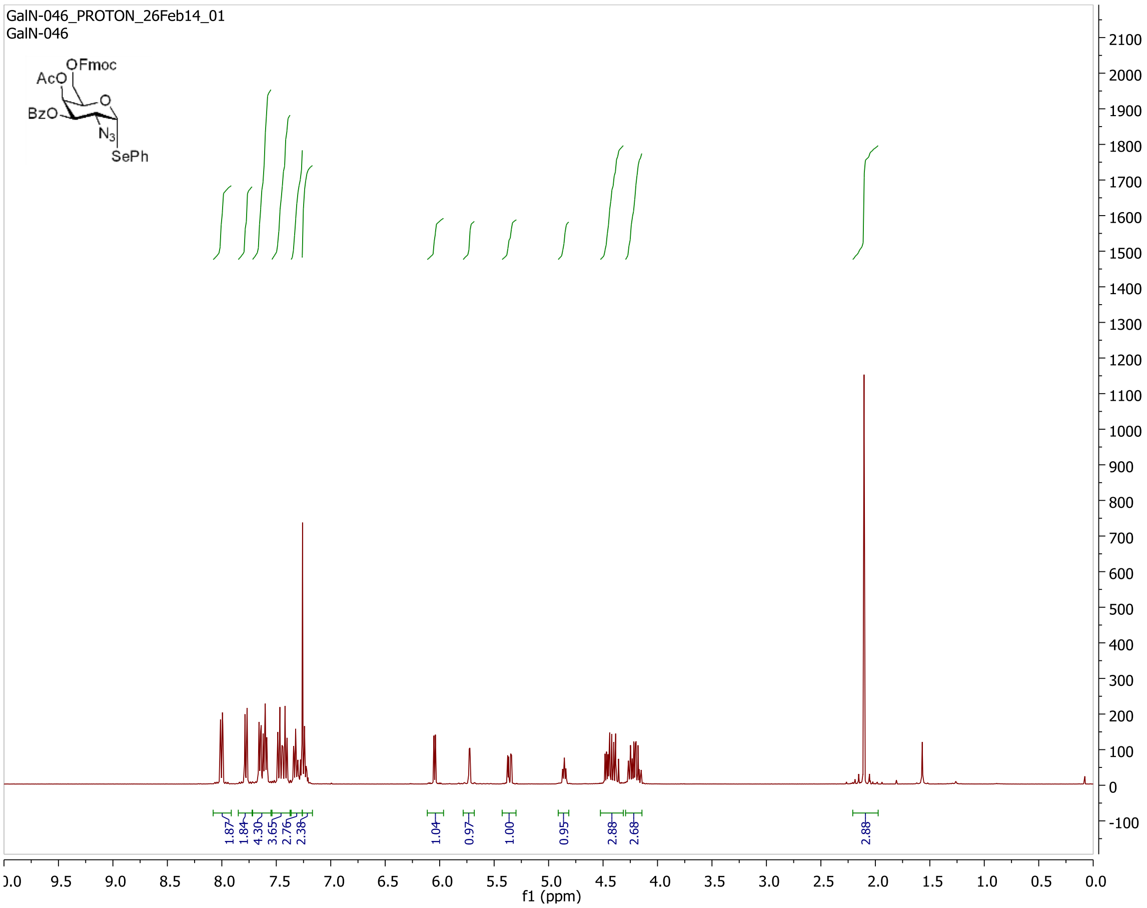

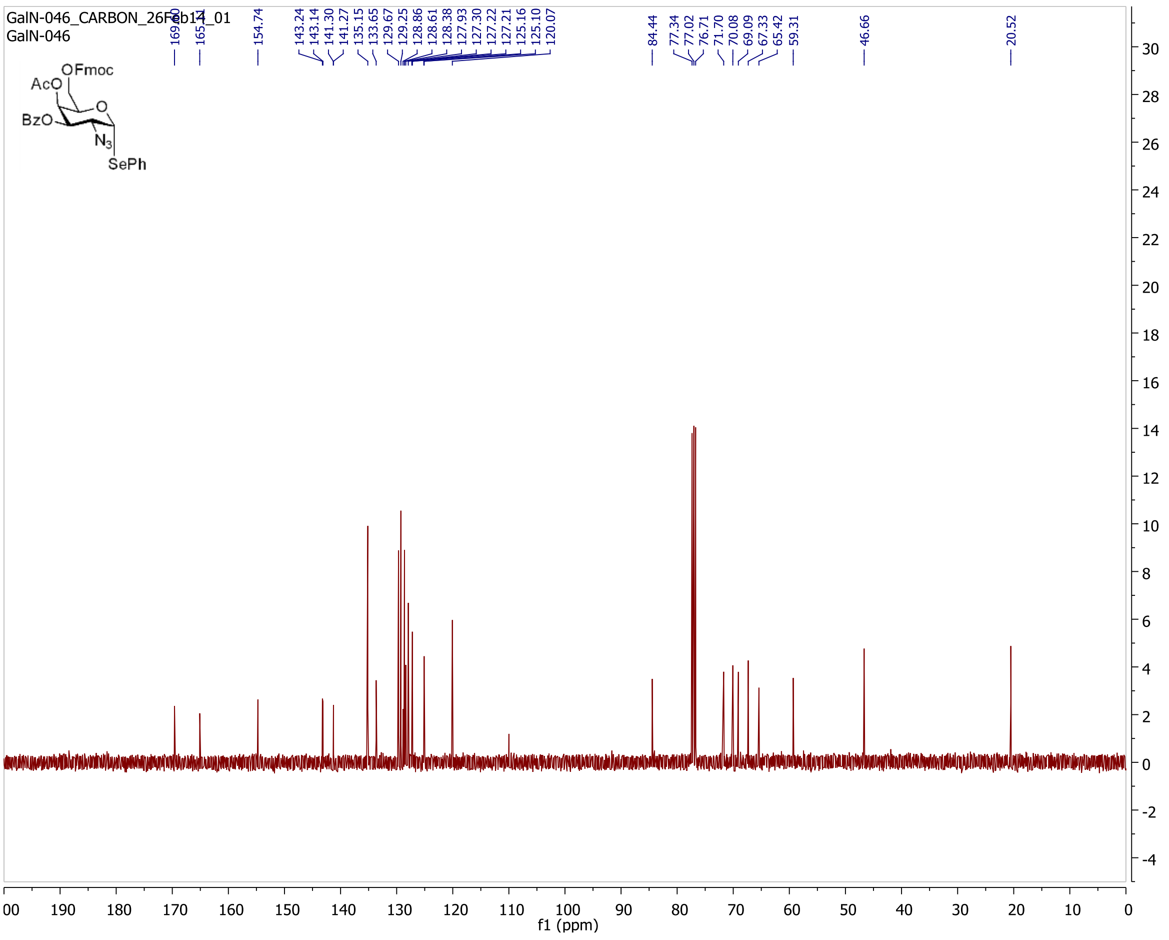
GalN-046_CARBON_26Feb14_01
GalN-046
f1 (ppm)

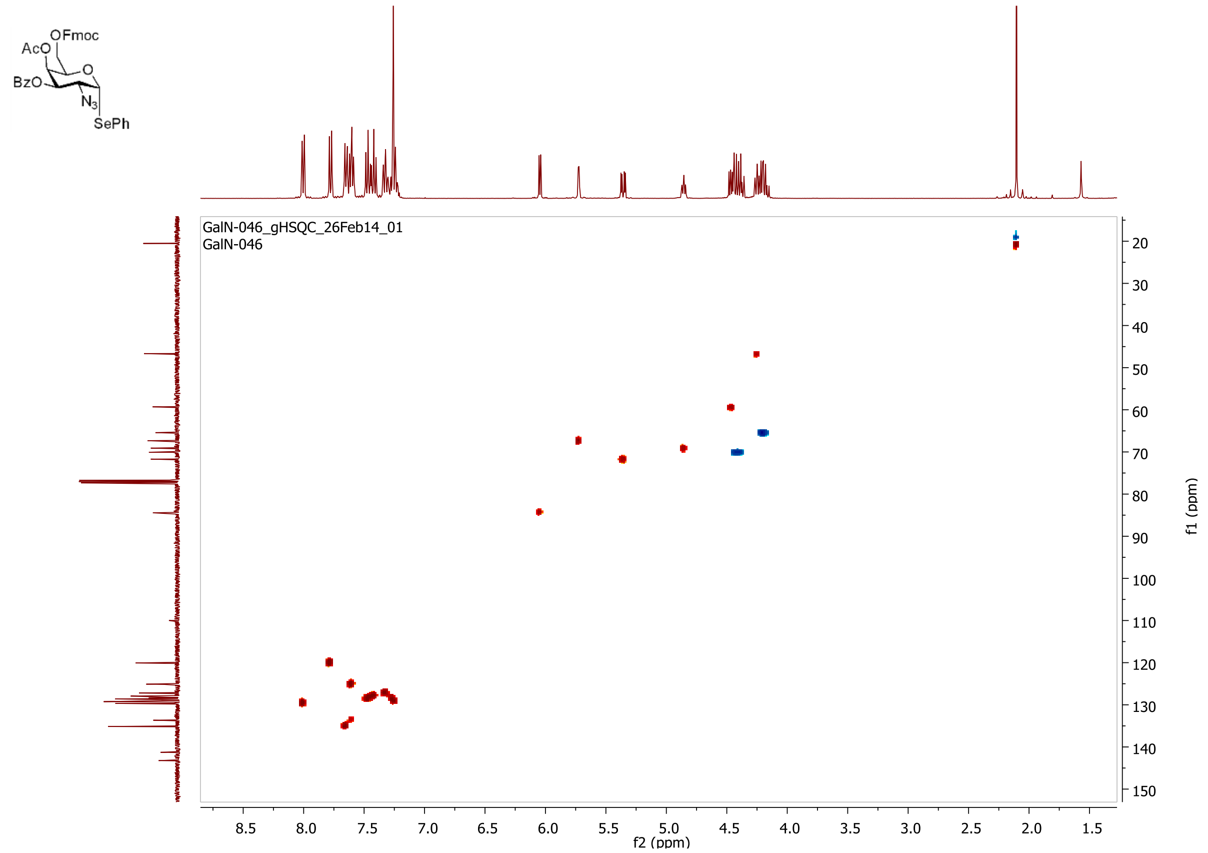
GalN-046_gHSQC_26Feb14_01
GalN-046
f2 (ppm)
f1 (ppm)

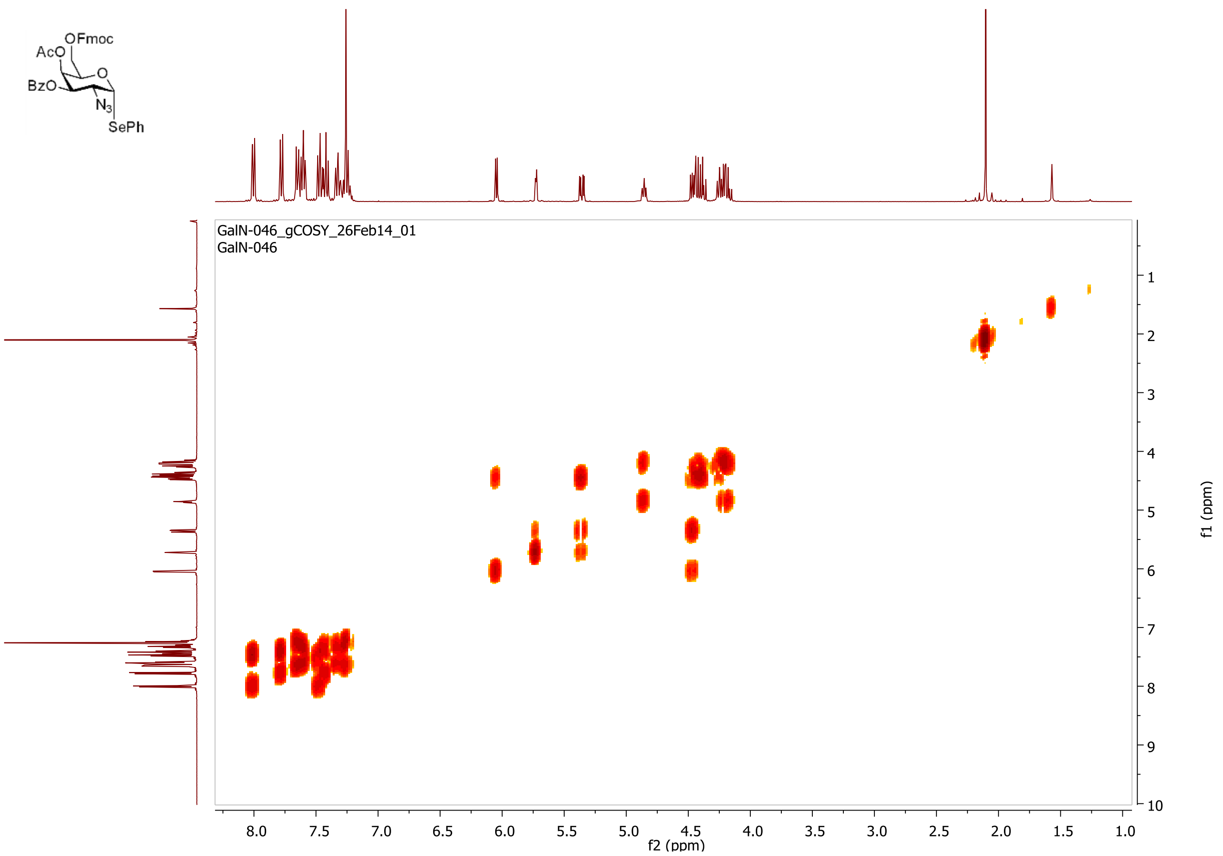

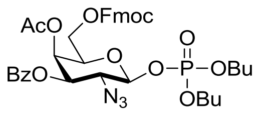

8

Di-*O*-butyl 2-deoxy-2-azido-3-*O*-benzoyl-4-*O*-acetyl-6-*O*-fluorenylmethoxycarbonyl-α-D-galactopyranosylphosphate (8)

To a solution of **8** (0.40 g, 0.56 mmol), dibutyl hydrogen phosphate (0.17 mL, 0.84 mmol) and 4 Å molecular scive in CH_2Cl_2 (5.61 mL) at -15 °C for 20 min, NIS (189 mg, 0.84 mmol) was added for 2 h. After checking by TLC, the reaction mixture was diluted with CH_2Cl_2 and quenched and extracted with saturated aqueous $Na_2S_2O_3$ and $NaHCO_3$ solution. The combined extracts were washed with brine, dried over $MgSO_4$ and then concentrated. The residue was purified by silica gel column chromatography (Hex/ EtOAc, form 50 /1 to 1/1) to give **8** (365 mg, 85%). Partial staring material **S7** (40 mg, 10%) was recovered. R_f = 0.39 (Hex/ EtOAc = 2/1); $[\alpha]_D^{20}$ = + 32.3 (c = 1.0, $CHCl_3$); ^{1}H NMR (400 MHz, $CDCl_3$) δ 7.99 (d, J = 6.5 Hz, 2H), 7.77 (d, J = 7.6 Hz, 2H), 7.65 – 7.54 (m, 3H), 7.51 – 7.37 (m, 4H), 7.36 – 7.29 (m, 2H), 5.61 (s, 1H), 5.20 (td, J = 8.0, 2.6 Hz, 1H), 5.13 (dt, J = 10.8, 2.7 Hz, 1H), 4.48 – 4.06 (m, 10H), 4.04 – 3.93 (m, 1H), 2.13 (s, 3H), 1.69 (dt, J = 14.7, 6.9 Hz, 4H), 1.43 (dt, J = 14.7, 4.5 Hz, 4H), 0.93 (qd, J = 8.2, 2.7 Hz, 6H). ^{13}C NMR (101 MHz, $CDCl_3$) δ 169.5,

165.0, 154.6, 143.2, 143.0, 141.2, 141.2, 133.7, 129.7, 128.7, 128.6, 127.9, 127.2, 125.2, 125.1, 120.0, 97.4 (d, J = 5.3 Hz), 71.7, 71.6, 70.3, 68.2 (dd, J = 12.7, 5.9 Hz), 66.2, 64.8, 61.6 (d, J = 9.3 Hz), 46.6, 32.1 (dd, J = 7.2, 4.7 Hz), 20.5, 18.6, 13.6. IR (thin film) 2962, 2114, 1752, 1602, 1451, 1378, 1260, 1220, 1067, 1025 cm^{-1}; HRMS (ESI) calcd. for $C_{38}H_{44}N_3O_{12}PNa$ $(M+Na)^+$ 788.2560, found 788.2551.

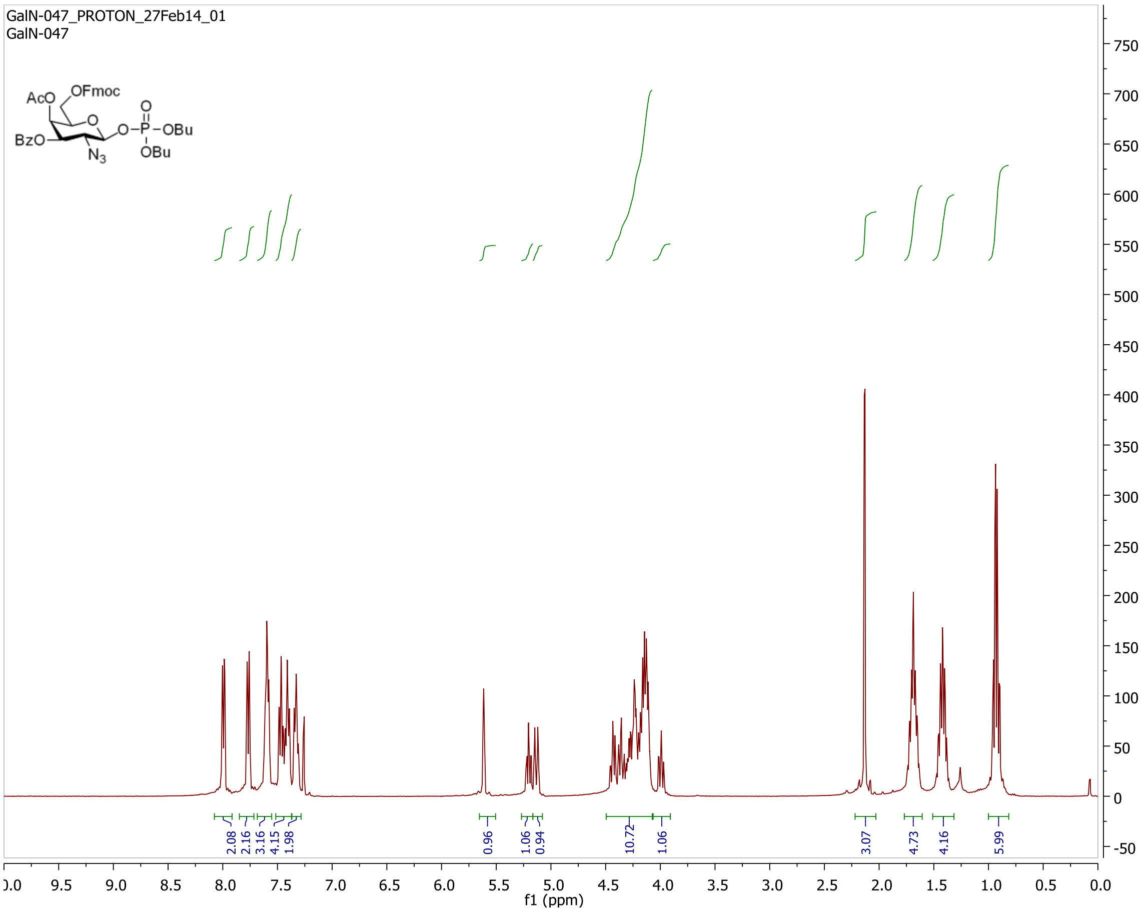

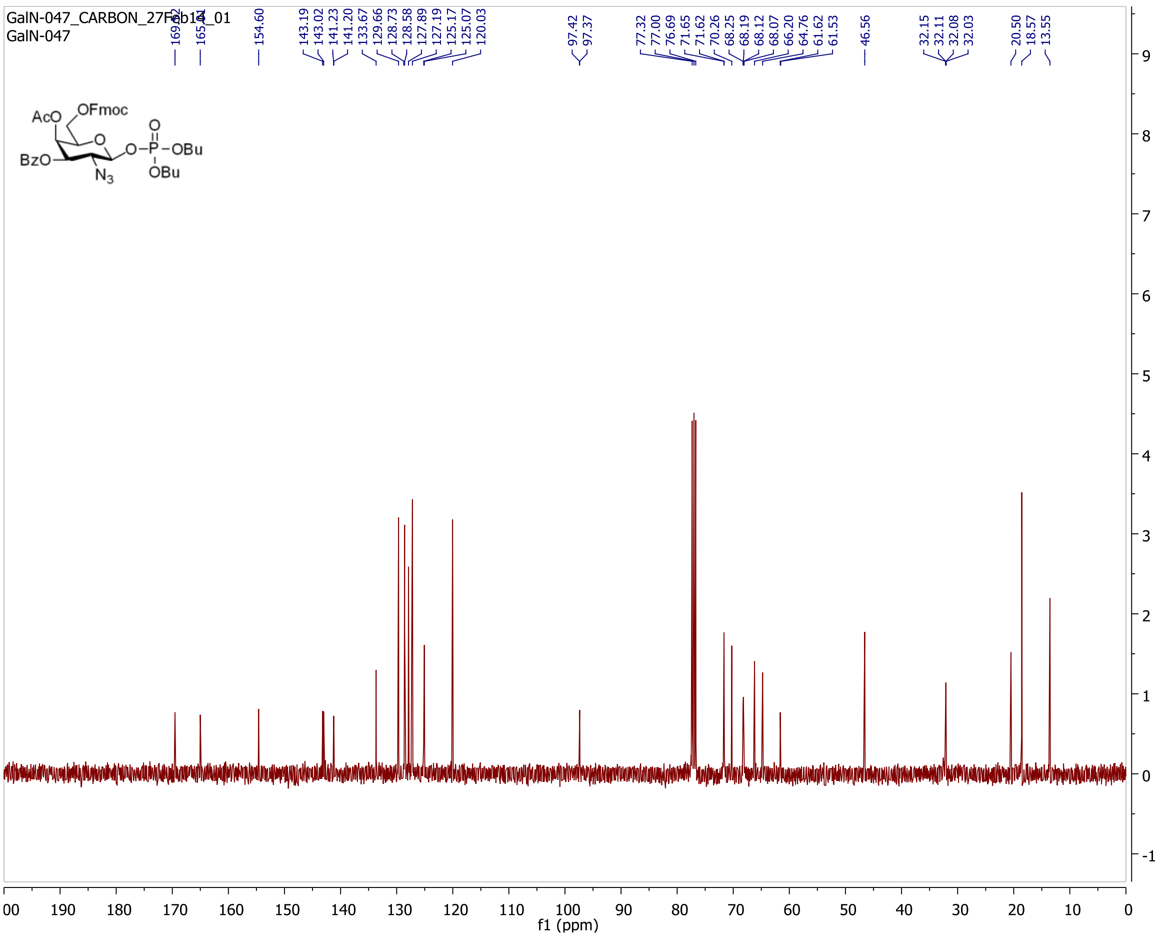
GalN-047_CARBON_27Feb14_01
GalN-047
AcO
OFmoc
BzO
N3
O
O–P–OBu
OBu
f1 (ppm)

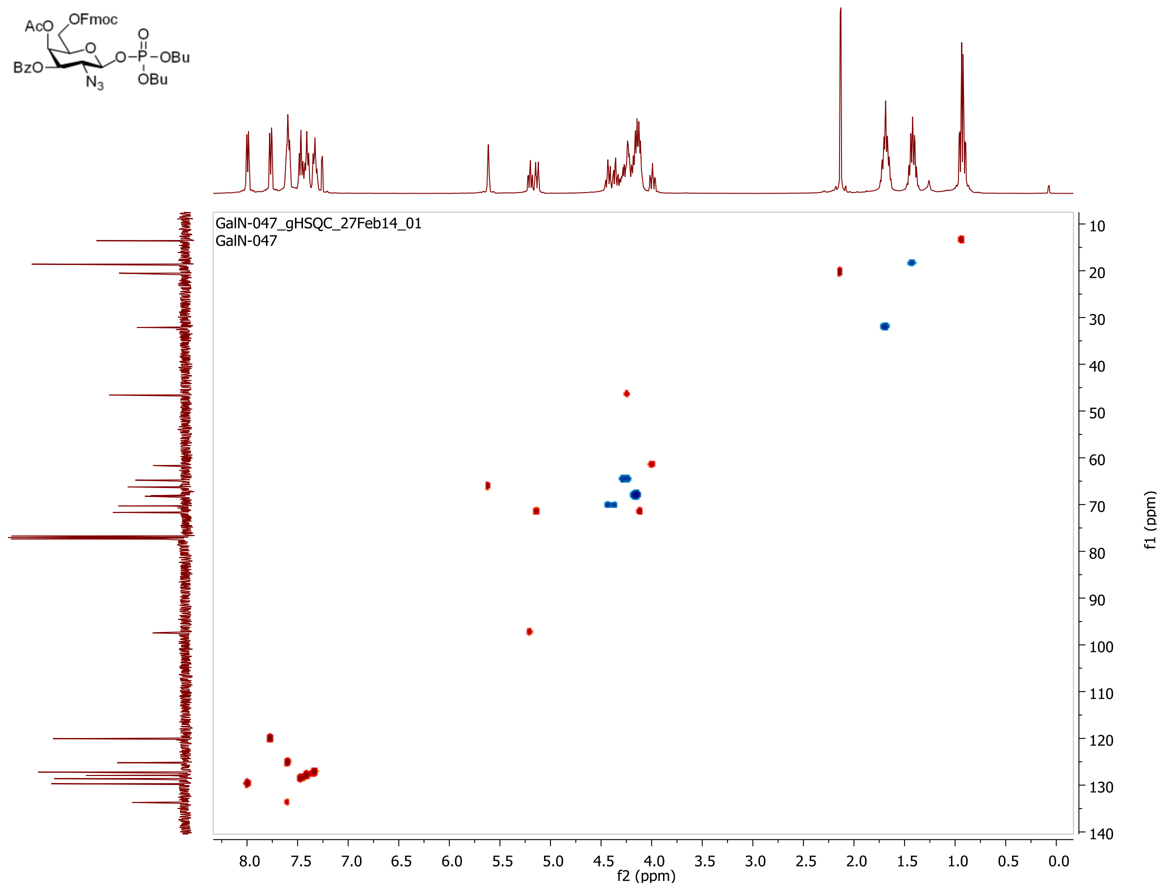
AcO
OFmoc
BzO
N3
O–P–OBu
OBu
GalN-047_gHSQC_27Feb14_01
GalN-047
f2 (ppm)
f1 (ppm)

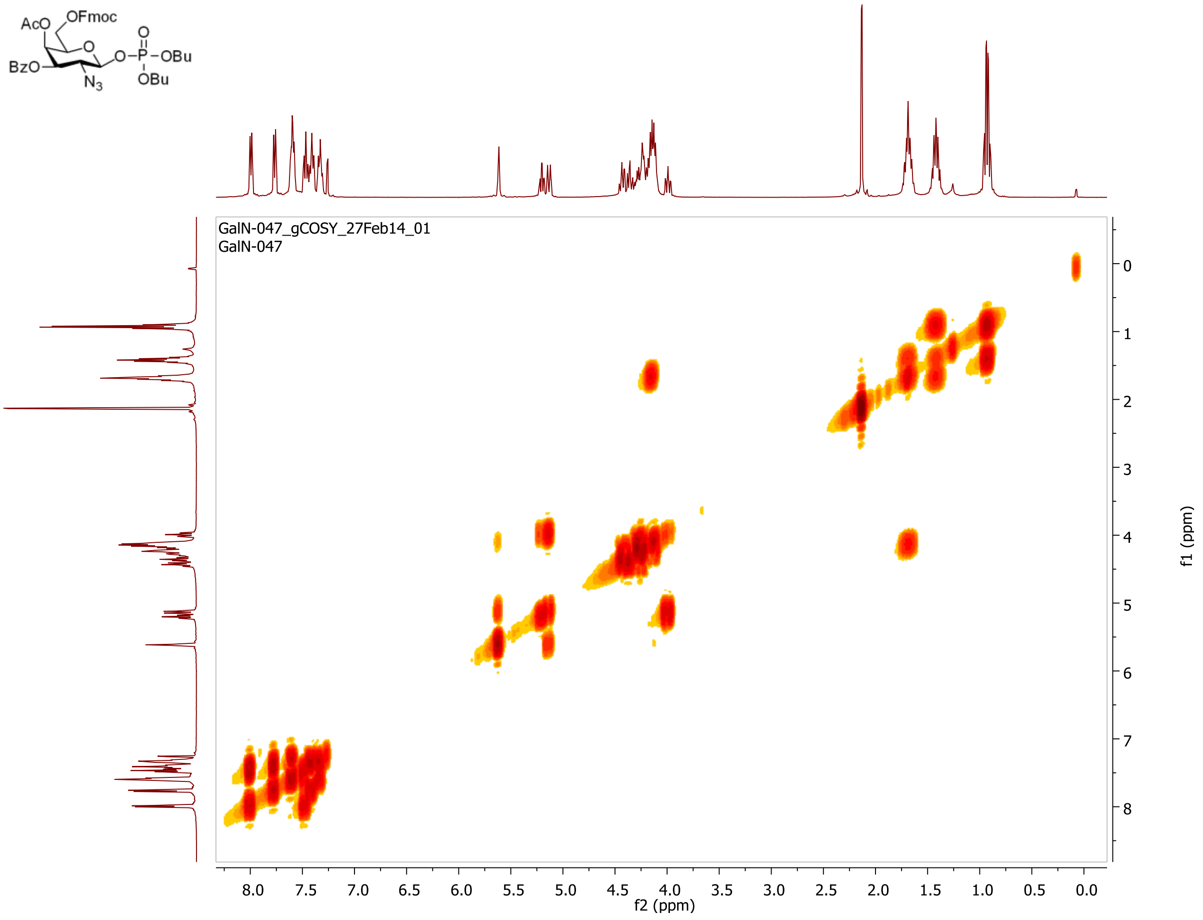

AcO
OFmoc
BzO
O
N3
O
P
OBu
OBu
GalN-047_gCOSY_27Feb14_01
GalN-047
f2 (ppm)
f1 (ppm)

Optimization of sialic acid building block activation temperature

Table S1. The optimization of sialic acid activation temperature was determined by mass-spectrometry.

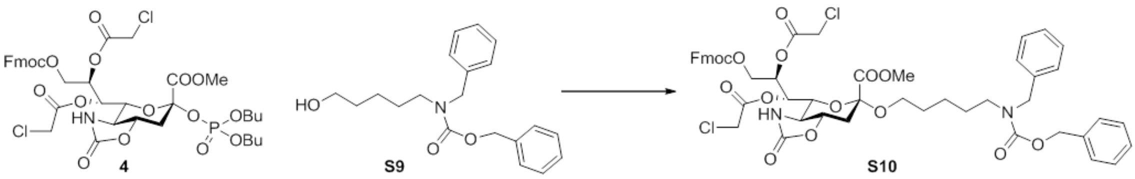

Entry	Temperture (°C)	Time (min)	Result
1	-78	10	No reaction
2	-60	10	No reaction
3	-50	10	No reaction
4	-40	10	No reaction
5	-30	10	No reaction
6	-20	10	No product. But BB started to eliminate
7	-20	60	Product formed, Reaction not finished, Elimination (minor), SM BB (major)
8	-10	30	Product formed, Reaction not finished, Elimination (major), SM BB (minor)
9	-5	20	Product formed, Reaction not finished, Elimination (major), SM BB (minor)
10	0	10	Product formed, Reaction not finished, Elimination (major), SM BB (minor)
11	5	10	Product formed, Reaction not finished, Elimination (major), SM BB (minor)

Experimental details:

Sialyl phosphate BB donor **4** were co-evaporated three times with toluene, and dried over in high vacuum for 1 h. Sialyl phosphate BB donor **4** (5 mg, 5.72 μmol) and linker **S9** (2.25 mg, 6.86 μmol) were dissolved in CH_2Cl_2/CH_3CN (1/1, 1.0 mL) in a heart-shaped flask under Ar and were then cooling down at different temperatures shown on Table S1 for 30 min. A TMSOTf (40 μL) activation solution in CH_2Cl_2/CH_3CN (1/1, 10 mL) was prepared under Ar. For each of reaction entry test, 250 μL of the above TMSOTf activation solution (corresponding to 5.72 μmol) was added dropwise to the reaction flask. A pyridine (20 μL) quenching solution in CH_2Cl_2 (10 mL) was prepared. After reaction for the times shown in

Table S1, the reaction mixture was quenched by adding pyridine quenching solution (0.5 mL, corresponding to 11.0 μmol). Each crude reaction mixture was analyzed by mass-spectrometry and TLC (Table S1).

Automated synthesis of sialylated oligosaccharides

General materials and methods

All solvents used were taken from a dry solvent system (jcmeyer-solvent systems). The building blocks are co-evaporated three times with toluene, and dried over in high vacuum for 1 h before use, and then dissolved in corresponding solvent under an Ar atmosphere and transferred into the vials that are placed on the corresponding ports in the synthesizer. Reagents are dissolved in the corresponding solvents under an Ar atmosphere in bottles that are placed on the ports of the synthesizer. Modules were modified based on previously described procedure.[3]

Preparation of reagent solutions

- ✧ **Building block solution:** 0.25 mmol of building block was dissolved in 2 mL of corresponding solvent.
  *For building blocks **4**, **5***: 1 mL CH_2Cl_2 and 1 mL CH_3CN.
  *For building blocks **6**, **7**, **9** and **10***: 2 mL CH_2Cl_2
  *For building block **8**:* 1 mL CH_2Cl_2 and 1 mL Dioxane.

- ✧ **Activator solution:**
  *For building blocks **4**, **5***: 480.0 μL TMSOTf was dissolved in 10 mL CH_2Cl_2 and 10 mL CH_3CN and purged the resulting solution with Ar for 1-2 min.
  *For building blocks **6**, **7**, **9** and **10***: *N*-Iodosuccinimide (1.35 g) was dissolved in a 3:1 mixture of anhydrous CH_2Cl_2 and dioxane (40.0 mL) and then TfOH (60 μL) was added and purged the resulting solution with Ar for 1-2 min.
  *For building block **8**:* 480.0 μL TMSOTf was dissolved in 20 mL CH_2Cl_2 and purged the resulting solution with Ar for 1-2 min.

- ✧ **Fmoc deprotection solution:** A solution of 20% NEt_3 in DMF (v/v) was prepared.
- ✧ **Acetylation capping solution:** Ac_2O was directly used.

- ✧ **Acidic wash solution:** TMSOTf solution form activation solution was directly used.

Modules for automated synthesis

Module A: Preparation of the resin ready for synthesis

For all compounds, automated syntheses were carried out on a 0.025 mmol scale using Merrifield supported photo-cleavable linker **11**[4] (70 mg, resin loading: 0.356 mmol/g). The Resin **11** was loaded into the reaction vessel of the synthesizer and swollen in 2 mL CH_2Cl_2 for at least 30 min. To start the synthesis sequence, the resin is washed consecutively with DMF, THF, and CH_2Cl_2 (three times each with 2 mL for 25 s). In all reactions, the resin was gently stirred by Ar bubble form the bottom of the reaction vessel.[5]

Module B: Acidic wash by TMSOTf solution

The resin was swollen in 2 mL CH_2Cl_2 and the temperature of the reaction vessel was adjusted to -20 °C. Upon the low temperature was reached, 350 μL of TMSOTf solution form phosphate activation solution was added dropwisely to the reaction vessel. After bubbling for one minute, the acidic solution was drained and the resin was washed with 2 mL CH_2Cl_2 for 25 s.

Module C: Glycosylation

Glycosylation reaction was performed after acidic wash. The CH_2Cl_2 was drained and the solution of thioglycoside or phosphate building block (5 eq. in 1.0 mL) was delivered to the reaction vessel. After the set temperature was reached, the reaction was started to add activator solution dropwisely (1.0 mL, 5 eq.). The glycosylations were performed at different temperatures for different build blocks. After completion of the reaction, the solution is drained and the resin was washed with CH_2Cl_2 (three times each with 2 mL for 25 s). This procedure was repeated twice.

Module D: Fmoc deprotection

The resin was washed with DMF (six times with 2 mL for 25 s), swollen in 2 mL DMF and the temperature of the reaction vessel was adjusted to 25 °C. For Fmoc deprotection, DMF was drained and then 2 mL of 20% NEt_3 in DMF was delivered into the reaction vessel. After 5 minutes, the reaction solution was collected in the fraction collector of the oligosaccharide synthesizer. This procedure was repeated three times. For the next glycosylation the resin is washed with DMF (three times with 3 mL for 25 s), THF, CH_2Cl_2 (three times each with 2 mL for 25 s)

Module E: Acetylation

The resin was washed pyridine (six times each with 2 mL for 25 s), swollen in 2 mL pyridine. The temperature of the reaction vessel was adjusted to 25 °C. The reaction was started by

addition of 1 mL of acetic anhydride to the reaction vessel. After 60 min, the reaction solution was drained and the resin was washed with CH_2Cl_2 and pyridine (six times with 2 mL for 25 s). This acetylation procedure is performed three times.

Post-synthesizer manipulations

Cleavage from the solid support[3, 4, 6]

The resin was swollen in 2 mL CH_2Cl_2 and taken up in a 10 mL glass syringe. Photo-reactor FEP tubing was washed with 20 mL CH_2Cl_2. The UV light source was a medium pressure Hg lamp with arc lengths of 27.9 cm and power of 450 W, surrounded by a Pyrex UV filter with 50% transmittance at 305 nm. For the cleavage reaction, the resin was slowly injected from the 10 mL glass syringe into the reactor and pushed through the tubing with 6 mL CH_2Cl_2 (flow rate: 500 µL per minute). To slowly react and wash out remaining resin in the tube, the resin was pushed with 20 mL CH_2Cl_2 (flow rate: 500 µL per min). The suspension leaving the reactor is directed into a filter (resin is filtered off). The entire procedure was performed twice to ensure the complete of cleavage, and finally the tube was washed with 20 mL CH_2Cl_2.

Purification

Solvent is evaporated in *vacuo* and the crude products were analyzed/ purified using analytical/preparative HPLC (Agilent 1200 Series spectrometer).

Automated synthesis of 12

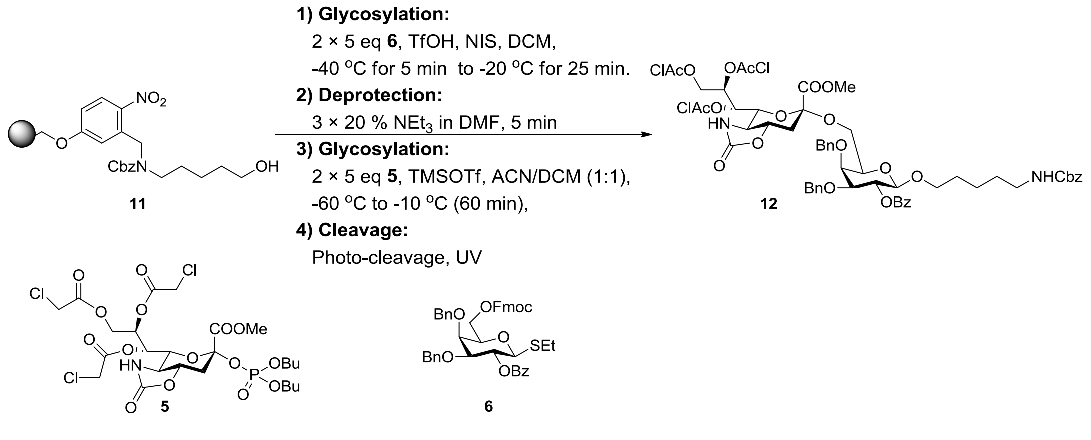

Table S2. Automated synthesis program for disaccharide **12**.

Steps	Automation process	Module
1	Preparation of the Resin Ready for Synthesis	A
2	Acidic Wash	B
3	Glycosylation: Donor **6**	C with NIS/ TfOH

		activation solution
4	Deprotection of Fmoc	D
5	Acidic Wash	B
6	Glycosylation: Donor **5**	C with TMSOTf activation solution

Cleavage, Analysis and Purification: Disaccharide **12** was cleaved from the solid support as described for Post-Synthesis Manipulations. The crude product was analyzed using normal phase analytical HPLC (YMC-Pack-Sil-NP; 5 µm, 150 mm, 4.6 mm; Linear gradient: EtOAc /Hexane; 10% EtOAc for 5 min, to 90% EtOAc in 30 min) and purified using preparative H PLC (YMC-Pack-Sil-NP; 5 µm, 150 mm, 20.0 mm, gradient: Hexane/EtOAc; 10-90% at 30 min) to obtain compound **12** (9.0 mg, 30% overall yield based on the resin loading).

*Analytical data for disaccharide **12**:* R_f, 0.41, EA/Hex (1/1); **$[\alpha]_D^{20}$** = + 5.29 (c = 1.00, $CHCl_3$); **IR** (thin film) ν_{max} = 3368, 2925, 2855, 1767, 1747, 1721, 1523, 1454, 1409, 1270, 1151, 1072, 1015, 738, 699 cm^{-1}; **^{1}H NMR** (400 MHz, $CDCl_3$) δ 8.00 (d, *J* = 7.6 Hz, 2H), 7.57 – 7.26 (m, 15H), 7.20 – 7.11 (m, 3H), 5.66 – 5.52 (m, 2H, H-2$_{Gal}$, H-8$_{sia}$), 5.27 (s, 1H, N*H*$_{sia}$), 5.15 (d, *J* = 9.8 Hz, 1H, H-7$_{sia}$), 5.04 (s, 2H, C*H$_2$*-Cbz), 5.00 (d, *J* = 11.5 Hz, 1H, C*H$_2$*-OBn×1), 4.68 (d, *J* = 11.5 Hz, 1H, C*H$_2$*-OBn×1), 4.63 (d, *J* = 12.3 Hz, 1H, C*H$_2$*-OBn×1), 4.60 – 4.53 (m, 1H, N*H*Cbz), 4.53 – 4.48 (m, 2H, H-9$_{sia}$, C*H$_2$*-OBn×1), 4.46 (d, *J* = 7.8 Hz, 1H, H-1$_{Gal}$), 4.37 – 4.25 (m, 2H, H-9$_{sia}$, C*H$_2$*Cl×1), 4.22 (d, *J* = 9.6 Hz, 1H, H-6$_{sia}$), 4.16 (d, *J* = 15.3 Hz, 1H, C*H$_2$*Cl×1), 4.12 (s, 2H, C*H$_2$*Cl×2), 4.03 (s, 2H, C*H$_2$*Cl×2), 3.96 – 3.78 (m, 4H, H-4$_{sia}$, H-4$_{Gal}$, H-6$_{Gal}$×1, O*C*H$_2$-linker×1), 3.68 – 3.63 (m, 1H, H-3$_{Gal}$), 3.62 (s, 3H, OC*H$_3$*), 3.57 (t, *J* = 6.4 Hz, 1H, H-5$_{Gal}$), 3.51 – 3.44 (m, 1H, H-6$_{Gal}$), 3.45 – 3.36 (m, 1H, O*C*H$_2$-linker×1), 3.08 (t, *J* = 10.2 Hz, 1H, H-5$_{sia}$), 2.96 – 2.81 (m, 3H, H-3$_{eq,sia}$, C*H$_2$*NHCbz-linker×2), 2.08 (t, *J* = 12.8 Hz, 1H, H-3$_{ax,sia}$), 1.46 – 1.35 (m, 2H), 1.33 – 1.21 (m, 2H), 1.21 – 1.11 (m, 2H); **^{13}C NMR** (101 MHz, $CDCl_3$) ^{13}C NMR (101 MHz, $cdcl_3$) δ 168.2 (C=O), 168.0 (C=O), 167.0 (C=O), 166.1 (C=O), 165.2 (C=O), 159.0 (C=O), 156.3 (C=O), 138.4, 137.6, 133.1, 130.1, 129.7, 128.5, 128.4, 128.3, 128.2, 128.1, 128.0, 127.8, 127.7, 127.5, 127.4, 101.5 (C-1$_{Gal}$), 100.1 (C-1$_{sia}$), 79.8 (C-3$_{Gal}$), 76.9 (C-4$_{sia}$), 74.1 (C-OBn), 73.4 (C-6$_{sia}$), 73.0 (C-5$_{Gal}$), 72.2 (C-4$_{Gal}$), 71.9 (C-2$_{Gal}$, C-OBn), 70.2 (C-7$_{sia}$), 69.4 (O*C*H$_2$-linker), 68.1 (C-8$_{sia}$), 66.5 (*C*-Cbz), 63.8 (C-6$_{Gal}$), 62.9 (C-9$_{sia}$), 57.6 (C-5$_{sia}$), 53.3 (O*C*H$_3$), 40.9 (*C*H$_2$NHCbz-linker), 40.8 (*C*H$_2$Cl), 40.4 (*C*H$_2$Cl), 40.3 (*C*H$_2$Cl), 37.2 (C-3$_{sia}$), 29.7, 29.4, 28.9, 23.1; **1D couple HMQC** (700 MHz, $CDCl_3$) $^3J_{C\text{-}1sia,\ H\text{-}3ax,sia}$ = 6.8 Hz; **ESI HR-MS**: m/z $[M+Na]^+$ calcd. for $C_{57}H_{63}Cl_3N_2O_{20}Na$ 1223.2937; Found 1223.2924.

Analytical NP-HPLC YMC-Pack-Sil of Crude Disaccharide **12** (280 nm trace)

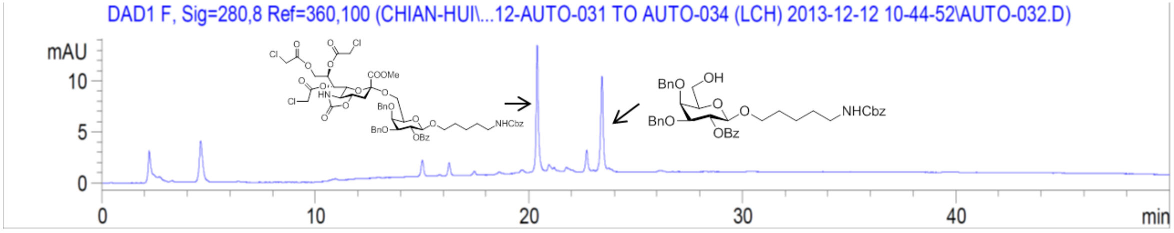

^{1}H NMR of 12

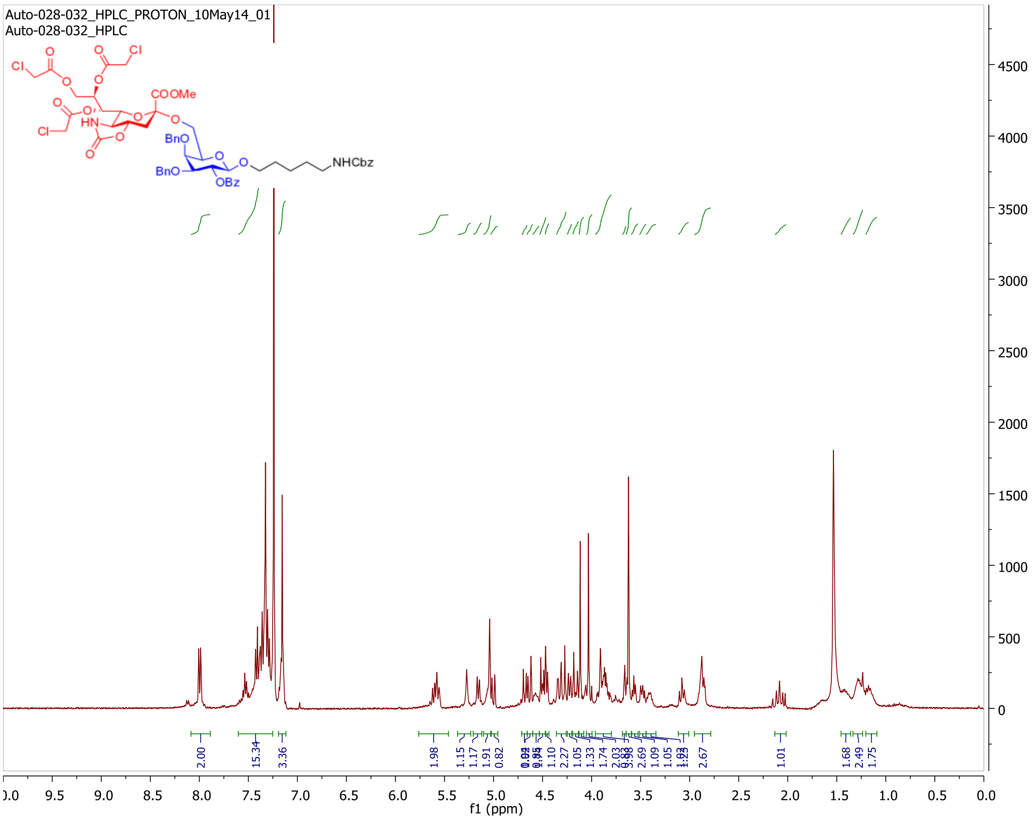

^{13}C NMR of 12

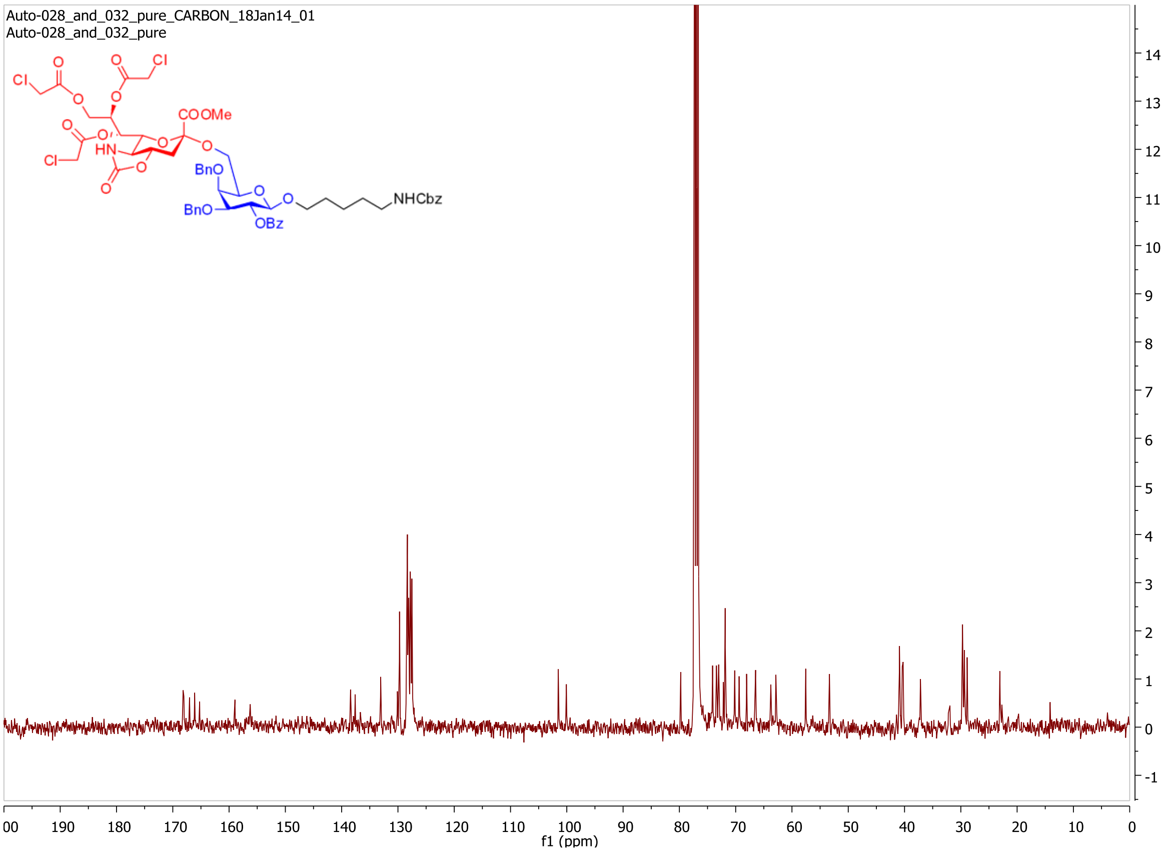

HSQC of 12

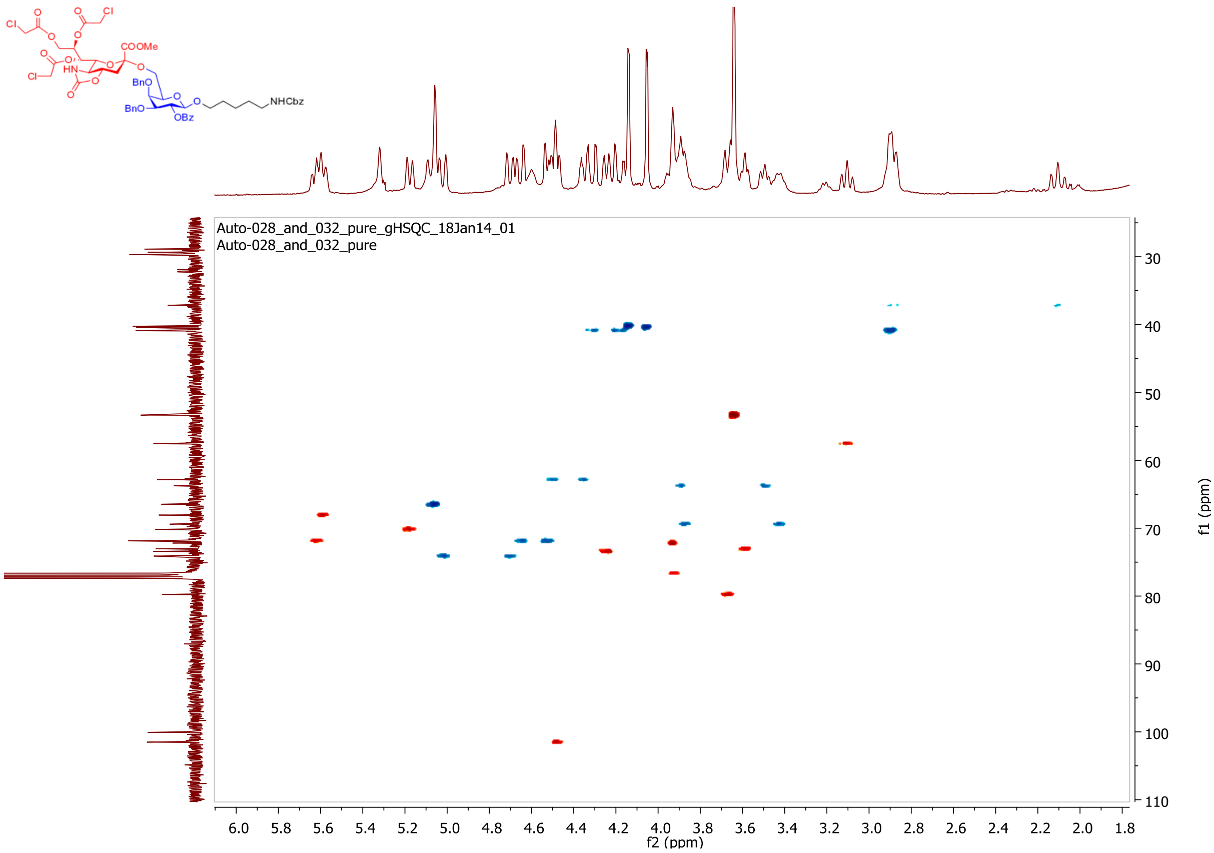

COSY of 12

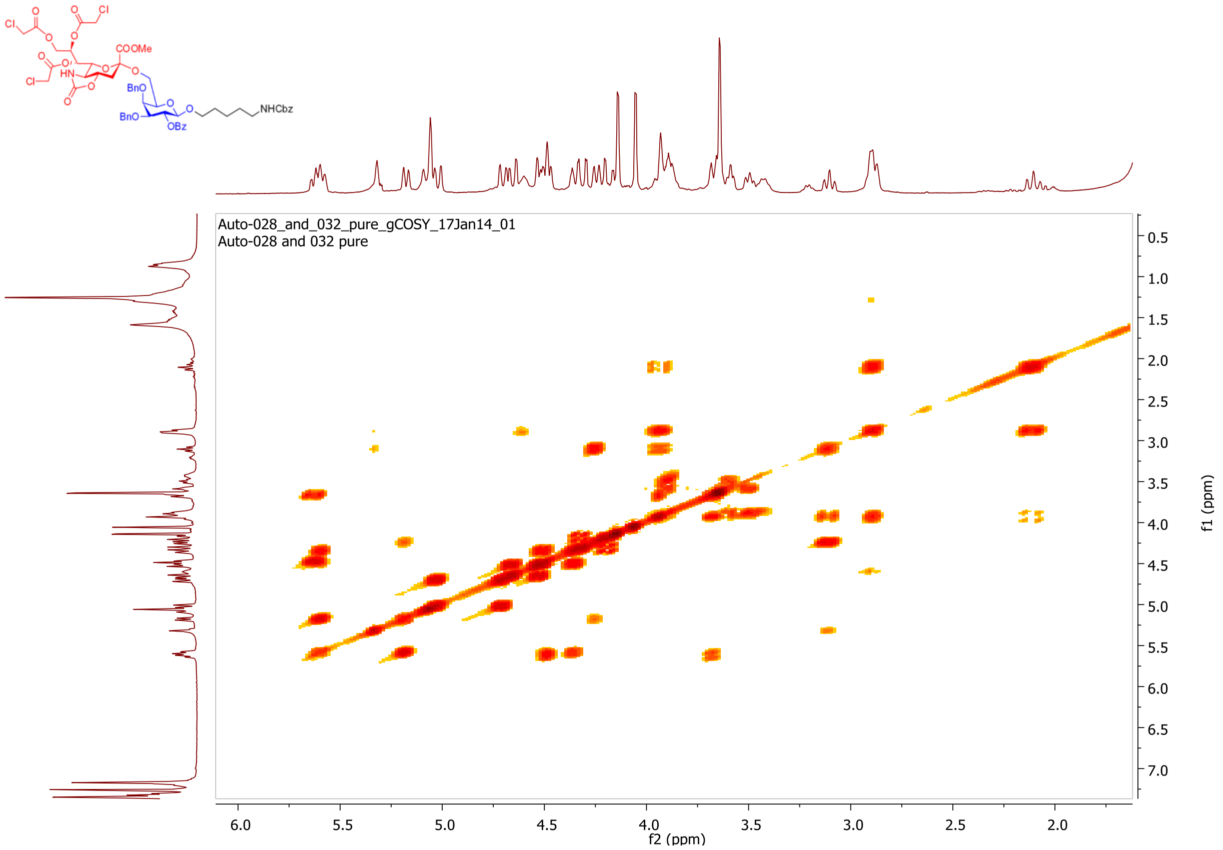

1D couple HMQC of 12

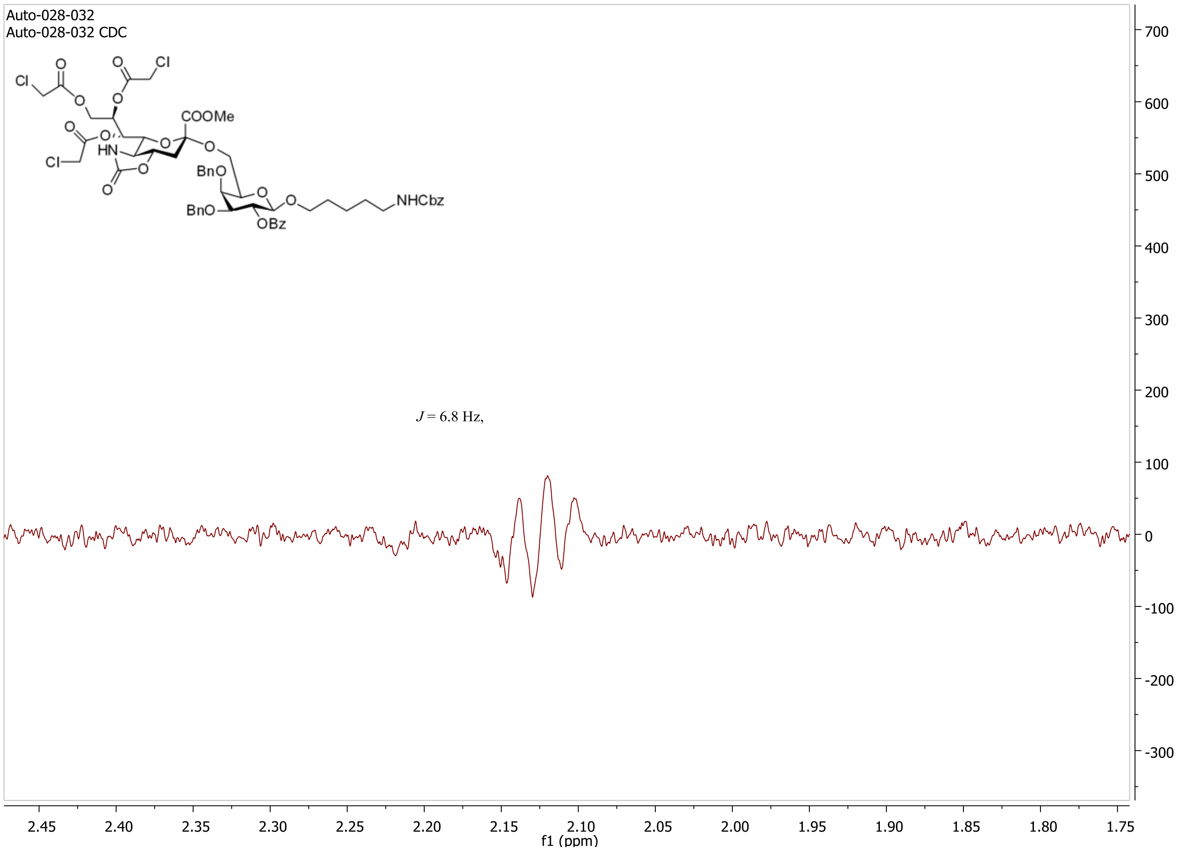

Automated Synthesis of 13

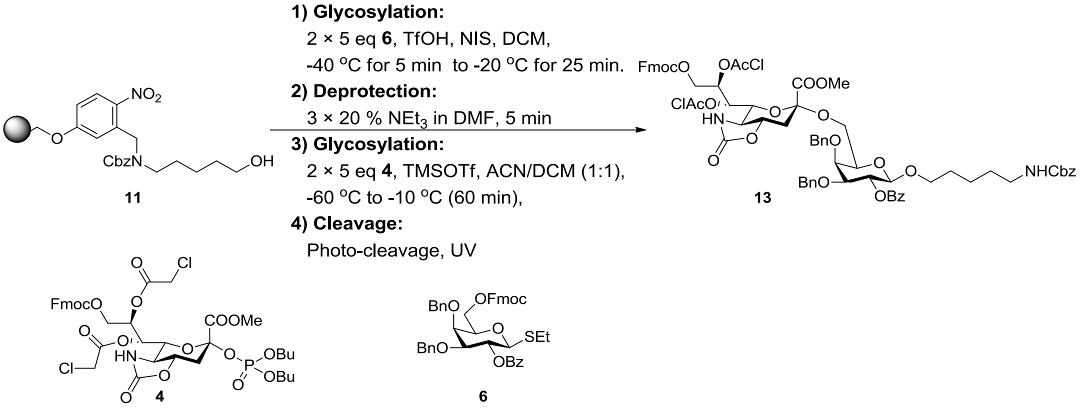

All the synthesis and purification details were same as **12**, exception of changing the sailyl phosphate build block **4** to obtain **13** in 40% (13.5 mg) overall yield based on the resin loading.

*Analytical data for disaccharide **13**:* R_f, 0.68, EA/Hex (1/1); $[\alpha]_D^{20}$ = + 3.3 (c = 0.66, $CHCl_3$); **IR** (thin film) ν_{max} = 3404, 3030, 2926, 2857, 1747, 1726, 1520, 1452, 1402, 1366, 1267, 1149, 1072, 1014, 758, 743, 711, 699 cm^{-1}; **^{1}H NMR** (400 MHz, $CDCl_3$) δ 8.01 (d, *J* = 7.2 Hz, 2H), 7.78 (d, *J* = 7.4 Hz, 2H), 7.57 (dd, *J* = 17.1, 7.3 Hz, 3H), 7.41 (dd, *J* = 15.9, 8.1 Hz, 6H), 7.37 – 7.29 (m, 10H), 7.21 – 7.10 (m, 5H), 5.62 (dd, *J* = 10.0, 8.0 Hz, 1H, H-2$_{Gal}$), 5.57 (d, *J* = 10.0 Hz, 1H, H-8$_{sia}$), 5.35 (s, 1H, N*H*$_{sia}$), 5.24 (dd, *J* = 10.0, 1.3 Hz, 1H, H-7$_{sia}$), 5.05 (s, 2H, C*H$_2$*-Cbz), 5.02 (d, *J* = 11.7 Hz, 1H, C*H$_2$*-OBn×1), 4.71 (d, *J* = 11.7 Hz, 1H, C*H$_2$*-OBn×1), 4.65 (d, *J* = 12.5 Hz, 1H, C*H$_2$*-OBn×1), 4.61 – 4.56 (m, 1H, N*H*Cbz), 4.51 (d, *J* = 12.5 Hz, 1H, C*H$_2$*-OBn×1), 4.47 (d, *J* = 8.0 Hz, 1H, H-1$_{Gal}$), 4.44 – 4.36 (m, 4H, H-9$_{sia}$×2, C*H$_2$*-Fmoc×2), 4.34 (d, J = 9.6 Hz, 1H, C*H$_2$*Cl×1), 4.30 – 4.19 (m, 3H, C*H$_2$*Cl×1, H-6$_{sia}$, C*H*-Fmoc), 4.20 – 4.08 (m, 2H, C*H$_2$*Cl), 4.02 – 3.77 (m, 4H, H-4$_{sia}$, H-4$_{Gal}$, H-6$_{Gal}$×1, O*C*H$_2$-linker×1), 3.72 – 3.65 (m, 1H, H-3$_{Gal}$), 3.64 (s, 3H, OC*H$_3$*), 3.58 (t, *J* = 6.3 Hz, 1H, H-5$_{Gal}$), 3.48 (m, 1H, H-6$_{Gal}$), 3.41 (dd, *J* = 14.8, 7.3 Hz, 1H, O*C*H$_2$-linker×1), 3.11 (t, *J* = 10.5 Hz, 1H, H-5$_{sia}$), 2.89 (m, 3H, H-3$_{eq,sia}$, C*H$_2$*NHCbz-linker ×2), 2.11 (t, *J* = 12.8 Hz, 1H, H-3$_{ax,sia}$), 1.54 – 1.37 (m, 2H), 1.35 – 1.19 (m, 2H), 1.23 – 1.09 (m, 2H); **^{13}C NMR** (101 MHz, $CDCl_3$) δ 168.2 (C=O), 168.1 (C=O), 166.2 (C=O), 165.2 (C=O), 159.0 (C=O), 156.3 (C=O), 154.9 (C=O), 143.1, 143.0, 141.3, 138.4, 137.6, 136.7, 133.1, 130.1, 129.7, 128.5, 128.4, 128.3, 128.2, 128.1, 128.0, 127.8, 127.7, 127.5, 127.4, 127.2, 125.1, 125.1, 120.1, 101.5 (C-1$_{Gal}$), 100.0 (C-2$_{sia}$), 79.7 (C-3$_{Gal}$), 77.2 (C-4$_{sia}$), 74.1 (C-OBn), 73.4 (C-6$_{sia}$), 73.0 (C-5$_{Gal}$), 72.1 (C-4$_{Gal}$), 71.9(C-2$_{Gal}$), 71.8(C-OBn), 70.5 (*C*H$_2$-OFmoc), 70.0 (C-7$_{sia}$), 69.4 (O*C*H$_2$-linker), 68.2 (C-8$_{sia}$), 66.5 (*C*-Cbz), 64.7 (C-9$_{sia}$), 63.7 (C-6$_{Gal}$), 57.6 (C-5$_{sia}$), 53.3 (O*C*H$_3$), 46.5 (*C*H-OFmoc), 41.0 (*C*H$_2$NHCbz-linker), 40.8(*C*H$_2$Cl), 40.4(*C*H$_2$Cl), 37.2(C-3$_{sia}$), 29.4, 28.9, 23.1; **1D couple HMQC** (700 MHz, $CDCl_3$) $^3J_{C\text{-}1sia,\ H\text{-}3ax,sia}$ = 6.3 Hz; **ESI HR-MS**: m/z $[M+Na]^+$ calcd. for $C_{70}H_{72}Cl_2N_2O_{21}Na$ 1369.3902; Found 1369.3872.

HPLC

Analytical NP-HPLC YMC-Pack-Sil of Crude Disaccharide **13** (280 nm trace)

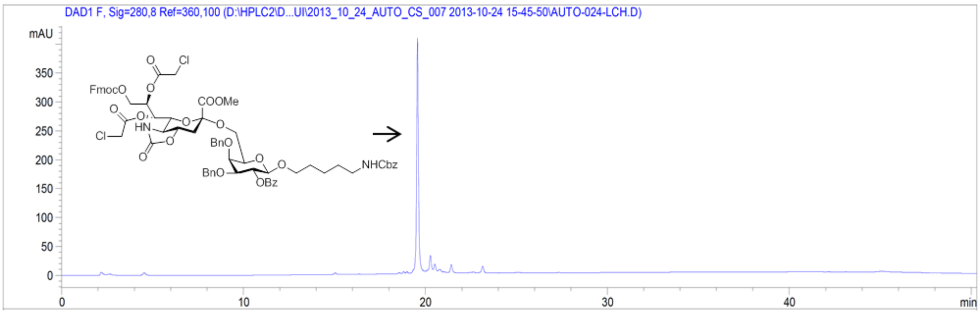
DAD1 F, Sig=280,8 Ref=360,100 (D:\HPLC2\D...UI\2013_10_24_AUTO_CS_007 2013-10-24 15-45-50\AUTO-024-LCH.D)
mAU
FmocO
COOMe
HN
Cl
BnO
BnO
OBz
NHCbz
min

^{1}H NMR of 13

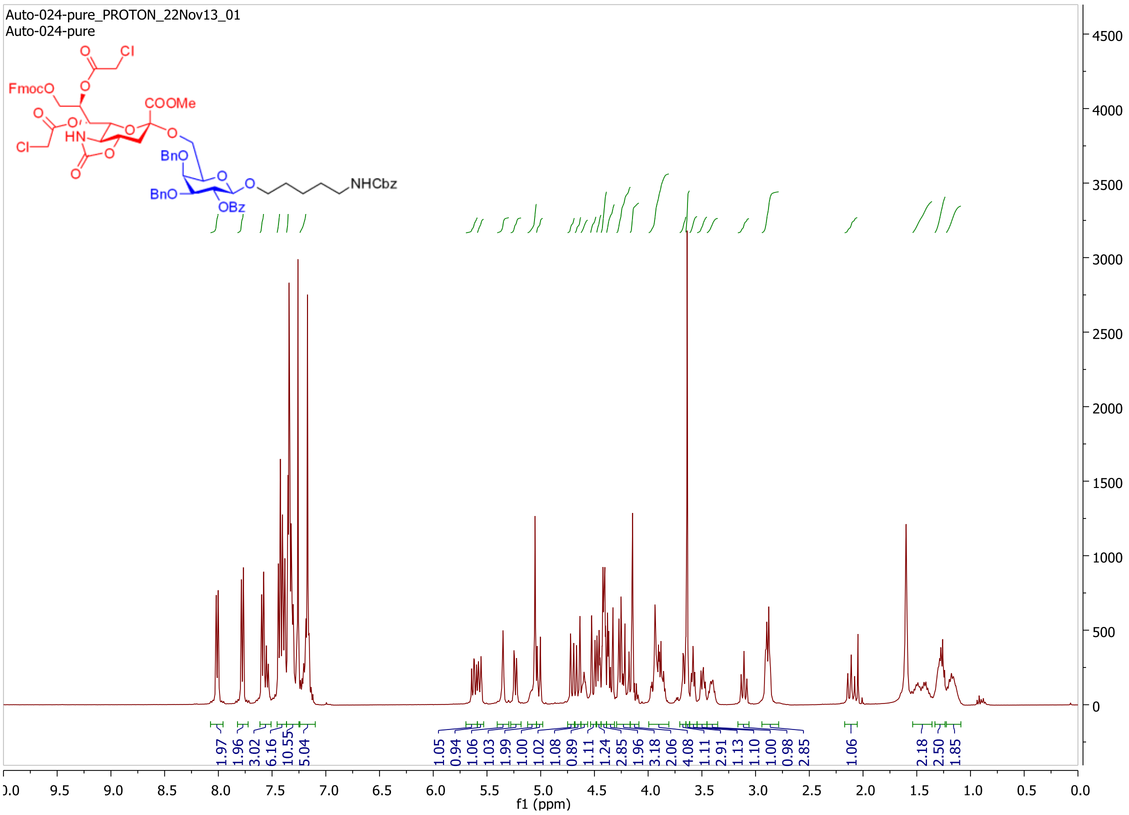
Auto-024-pure_PROTON_22Nov13_01
Auto-024-pure
FmocO
COOMe
HN
Cl
BnO
BnO
OBz
NHCbz
f1 (ppm)

^{13}C NMR of 13

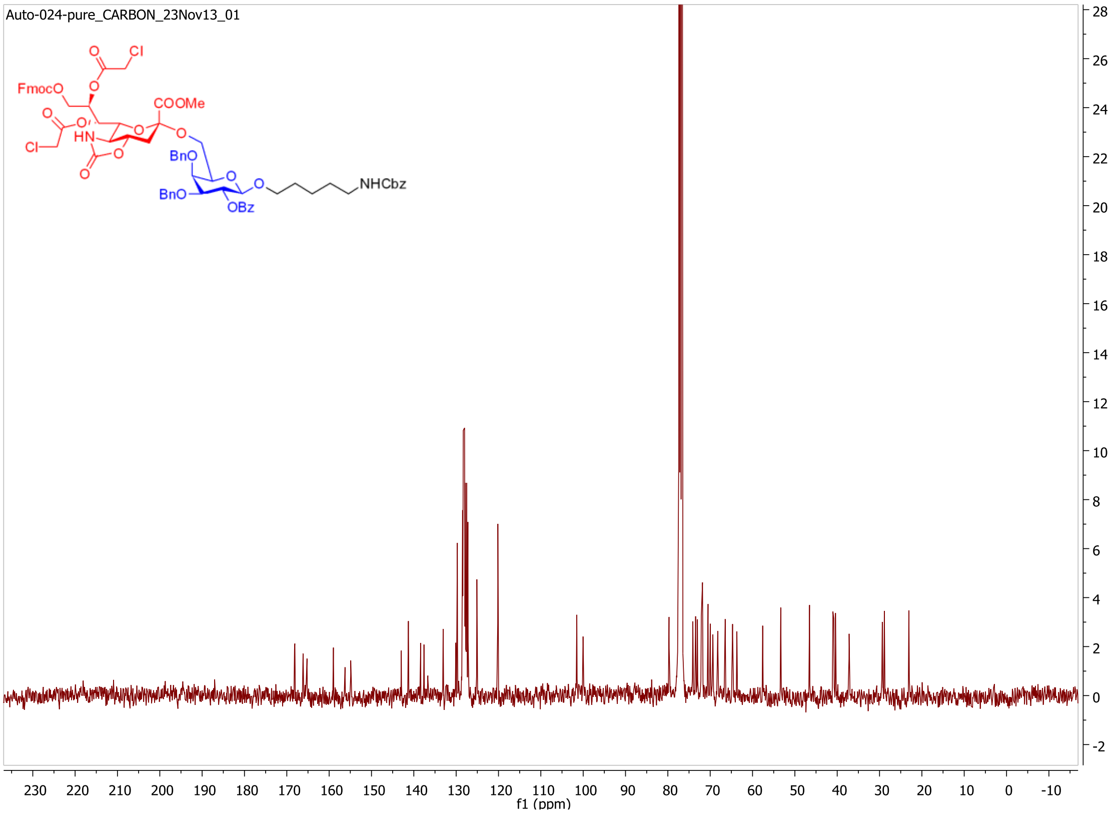

HSQC of 13

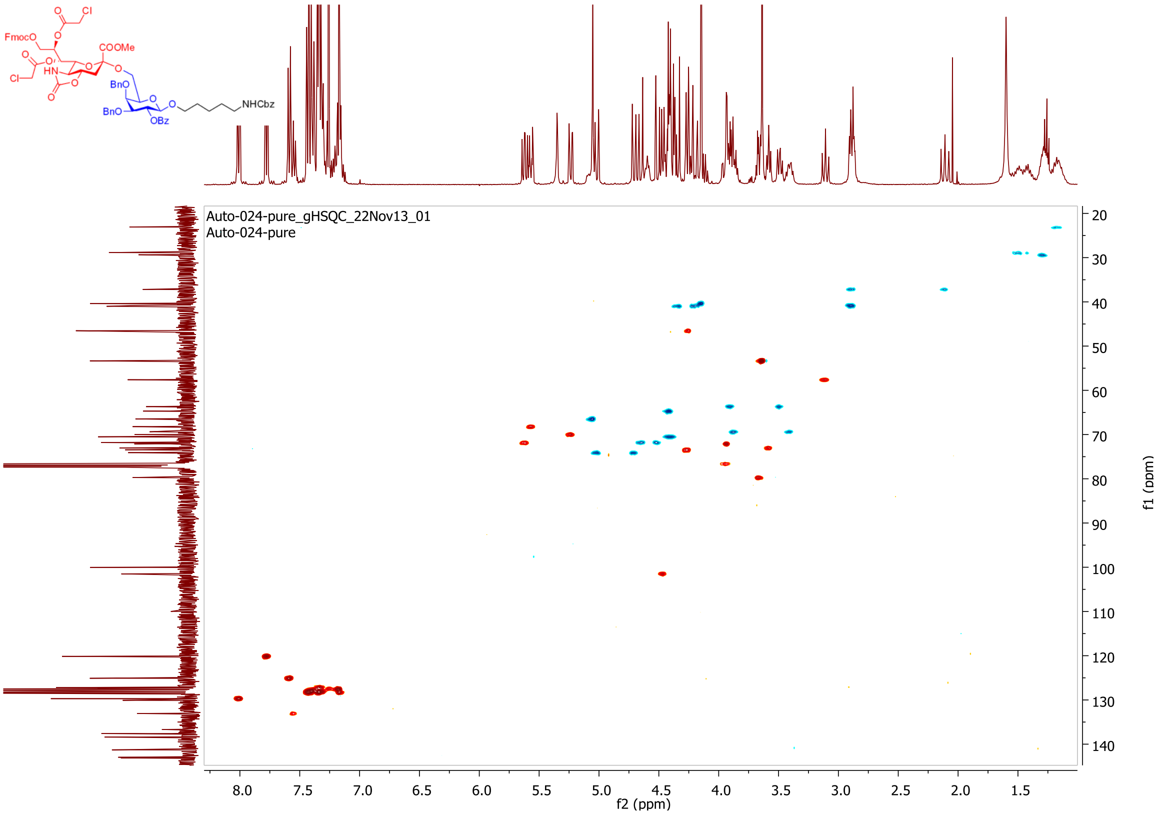

COSY of 13

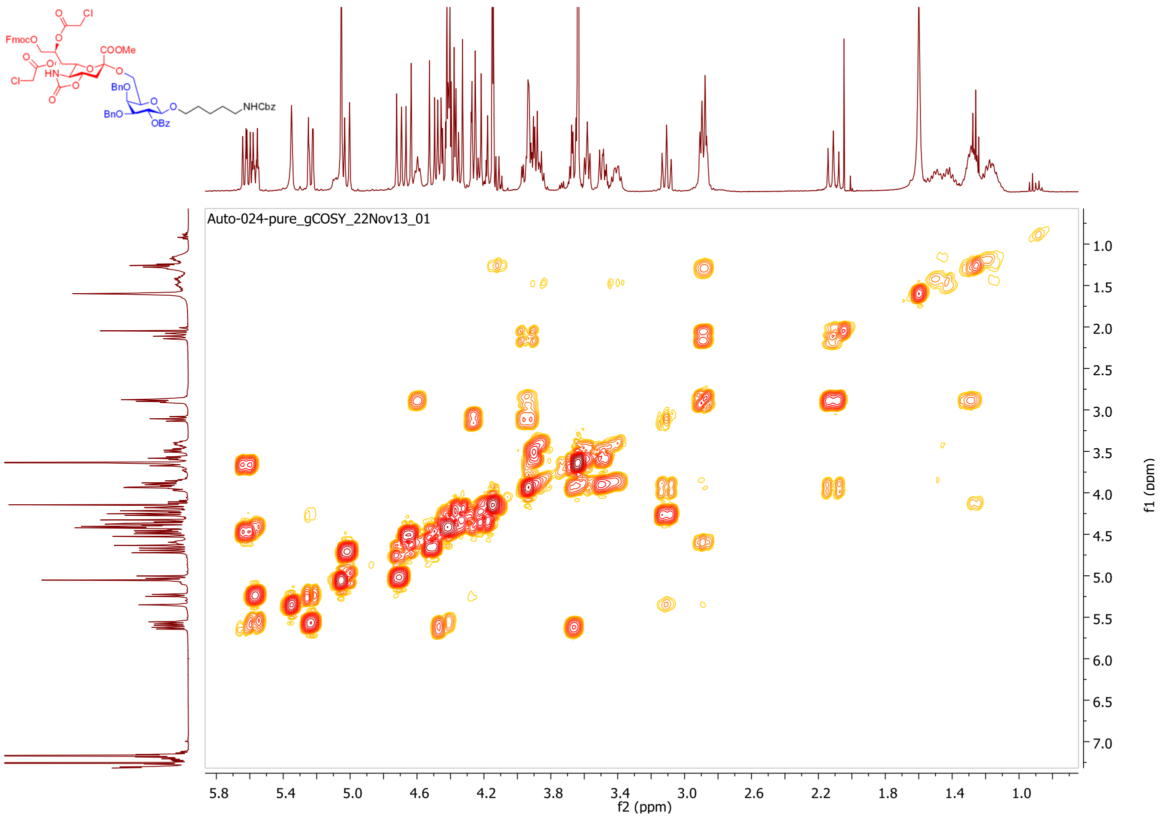

1D couple HMQC of 13

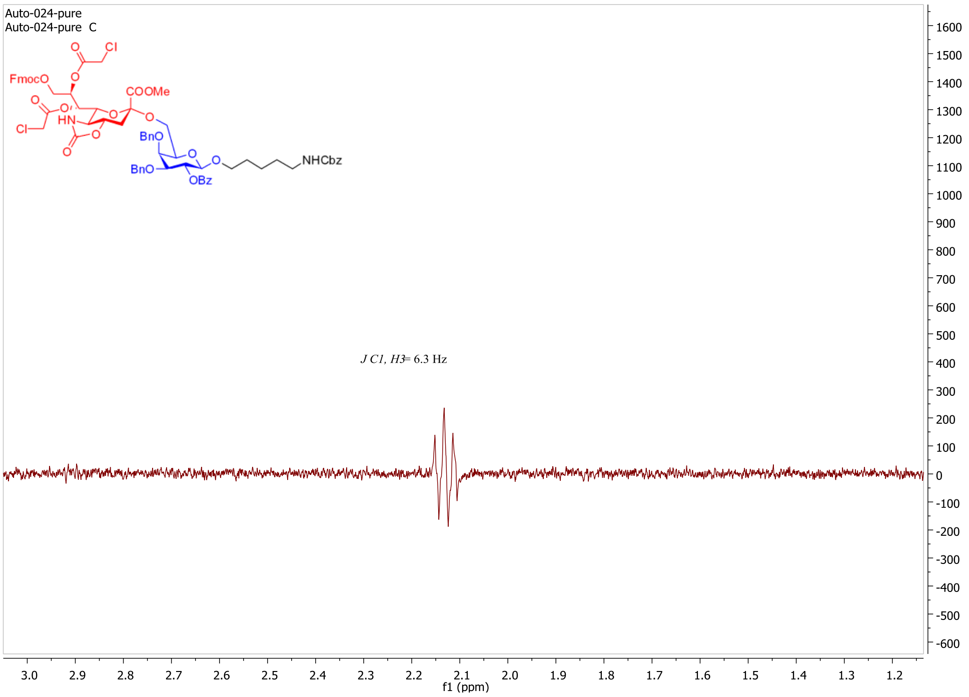

Automated Synthesis of 14

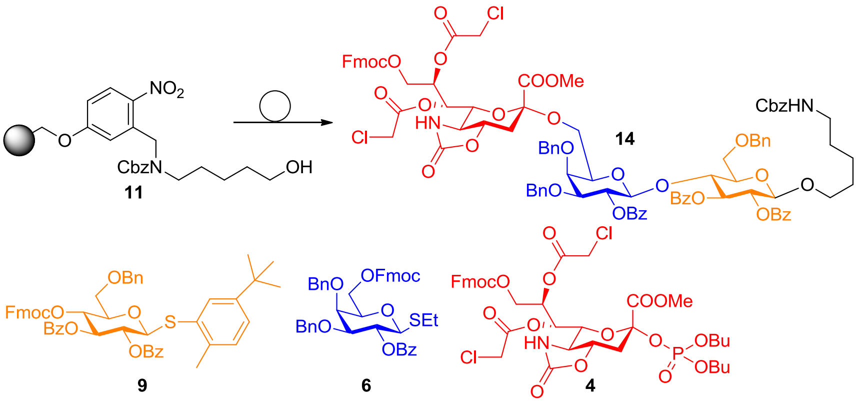

Reaction Condtions:

1) Glycosylation:
2 × 5 eq **9**, TfOH, NIS, DCM,
-30 °C for 5 min to -10 °C for 25 min.

2) Deprotection:
3 × 20 % NEt_3 in DMF, 5 min.

3) Glycosylation:
2 × 5 eq **6**, TfOH, NIS, DCM,
-40 °C for 5 min to -20 °C for 30 min.

4) Deprotection:
3 × 20 % NEt_3 in DMF, 5 min.

5) Glycosylation:
2 × 5 eq **4**, TMSOTf, ACN/DCM (1:1),
temperature in Table S4

6) Cleavage:
Photo-cleavage, UV

Table S3. Automated synthesis program for tri-saccharide **14**.

Steps	Automation process	Module
1	Preparation of the Resin Ready for Synthesis	A
2	Acidic Wash	B
3	Glycosylation: Donor **9**	C with NIS/ TfOH activation solution
4	Deprotection of Fmoc	D
5	Acidic Wash	B
6	Glycosylation: Donor **6**	C with NIS/ TfOH activation solution
7	Deprotection of Fmoc	D
8	Acidic Wash	B
9	Glycosylation: Donor **4**	C with TMSOTf activation solution

Table S4. Different sialylation temperatures for synthesis of **14.**

Entry	Sialylation condition	Purified yield
1	-60 °C (10 min), -50 °C (10 min), -40 °C (10 min), -30 °C (10 min), -20 °C (10 min), -10 °C (10 min),	6 mg
2	-60 °C (10 min), -50 °C (30 min), -40 °C (30 min), -30 °C (30 min), -20 °C (10 min), -10 °C (10 min),	10 mg
3	-50 °C (5 min), -30 °C (10 min), -20 °C (80 min), -10 °C (30 min), 0 °C (10 min)	10 mg

Analysis and Purification: tri-saccharide **14** was cleaved from the solid support as described in Post-Synthesizer Manipulations. The crude product was analyzed using normal phase analytical HPLC (YMC-Pack-Sil-NP; 5 µm, 150 mm, 4.6 mm; Linear gradient: EtOAc

/Hexane; 10% EtOAc for 5 min, to 90% EtOAc in 30 min) and purified using preparative HPLC (YMC-Pack-Sil-NP; 5 µm, 150 mm, 20.0 mm, gradient: Hexane/EtOAc; 10-90% in 30 mins) to obtain compound **14** (10.0 mg, 22% overall yield based on the resin loading).

Analytical data for tri-saccharide ***14****:* R_f, 0.58, EA/Hex (1/1); **$[\alpha]_D^{20}$** = +19.16 (c = 1.00, $CHCl_3$); **IR** (thin film) ν_{max} = 3403, 2926, 2866, 1732, 1602, 1519, 1452, 1267, 1096, 1071, 1027, 742, 711 cm^{-1}; **^{1}H NMR** (400 MHz, $CDCl_3$) δ 8.00 – 7.83 (m, 6H), 7.76 (d, J = 7.6 Hz, 2H), 7.61 – 7.26 (m, 28H), 7.20 – 7.05 (m, 7H), 5.66 (t, J = 9.1 Hz, 1H, H-3_{Glc}), 5.57 (d, J = 9.8 Hz, 1H, H-8_{sia}), 5.47 (dd, J = 9.5, 8.0 Hz, 1H, H-2_{Gal}), 5.35 (s, 1H, NH_{sia}), 5.26 (t, J = 8.4 Hz, 1H, H-2_{Glc}), 5.18 (d, J = 9.8 Hz, 1H, H-7_{sia}), 5.02 (s, 2H, CH_2-Cbz), 4.88 (d, J = 11.7 Hz, 1H, CH_2-OBn×1), 4.64 (d, J = 8.0 Hz, 1H, H-1_{Gal}), 4.59 – 4.08 (m, 16H, CH_2-OBn×5, CH, CH_2-Fmoc×3, H-6_{sia}, H-9_{sia}×2, CH_2Cl×2, H-1_{Glc}, H-4_{Glc}, NHCbz), 3.99 – 3.85 (m, 3H, CH_2Cl×2, H-4_{sia}), 3.84 – 3.75 (m, 2H, OCH_2-linker×1, H-4_{Gal}), 3.68 – 3.59 (m, 3H, H-5_{Glc}, H-6_{Glc}×2), 3.57 (s, 3H, OCH_3), 3.52 – 3.43 (m, 2H, H-3_{Gal}, H-6_{Gal}×1), 3.37 (dd, J = 15.4, 6.6 Hz, 1H, OCH_2-linker×1), 3.28 (t, J = 8.5 Hz, 1H, H-6_{Gal}×1), 3.24 – 3.15 (m, 1H, H-5_{Ga}), 3.02 (t, J = 10.4 Hz, 1H, H-5_{sia}), 2.93 – 2.81 (m, 2H, CH_2NHCbz-linker×2), 2.71 (dd, J = 12.8, 3.1 Hz, 1H, H-$3_{eq,sia}$), 1.86 (t, J = 12.8 Hz, 1H, H-$3_{ax,sia}$), 1.42 (s, 2H), 1.32 – 1.24 (m, 2H), 1.19 – 1.06 (m, 2H); **^{13}C NMR** (150 MHz, $CDCl_3$) 168.3, 168.1, 166.1, 165.4, 165.2, 165.0, 159.0, 156.3, 155.0, 143.3, 143.1, 141.4, 138.7, 138.3, 137.7, 136.8, 133.2, 133.1, 132.9, 130.2, 129.9, 129.8, 129.8, 129.7, 128.6, 128.4, 128.3, 128.2, 128.1, 127.9, 127.7, 127.4, 127.2, 125.2, 125.2, 120.2, 100.9, 100.4, 100.0, 80.0, 76.7, 75.7, 75.2, 74.6, 74.0, 73.6, 73.3, 73.1, 72.6, 72.2, 71.9, 71.7, 70.6, 70.1, 69.6, 68.6, 68.3, 66.5, 64.7, 62.2, 57.8, 53.3, 46.7, 41.1, 40.9, 40.4, 36.5, 29.8, 29.4, 23.1; **1D couple HMQC** (700 MHz, $CDCl_3$) $^3J_{C\text{-}1sia,\ H\text{-}3ax,sia}$ = 7.0 Hz; **ESI HR-MS**: m/z $[M+Na]^+$ calcd. for $C_{97}H_{96}Cl_2N_2O_{28}Na$ 1829.5424; Found 1829.5389.

Analytical NP-HPLC YMC-Pack-Sil of Crude triaccharide **14** (ELSD trace)

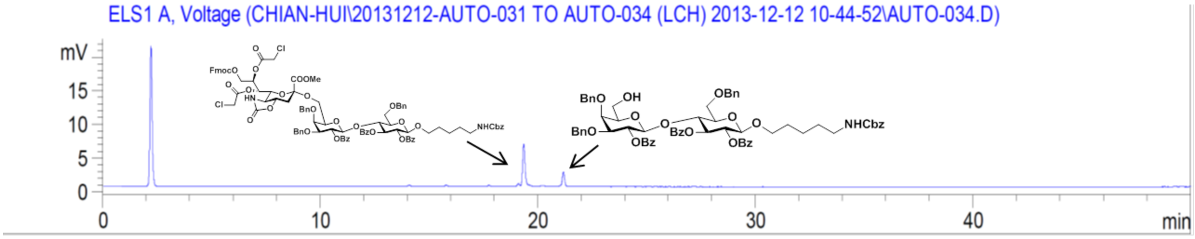

^{1}H NMR of 14

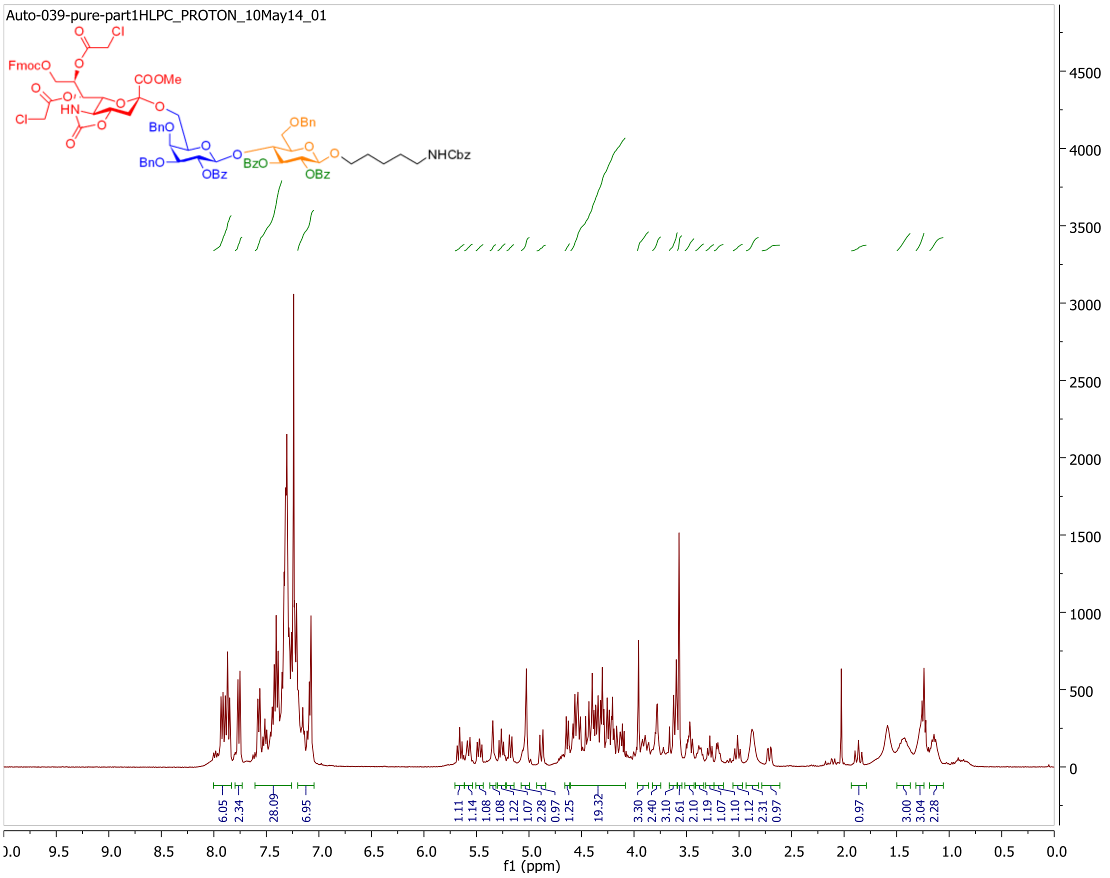

^{13}C NMR of 14

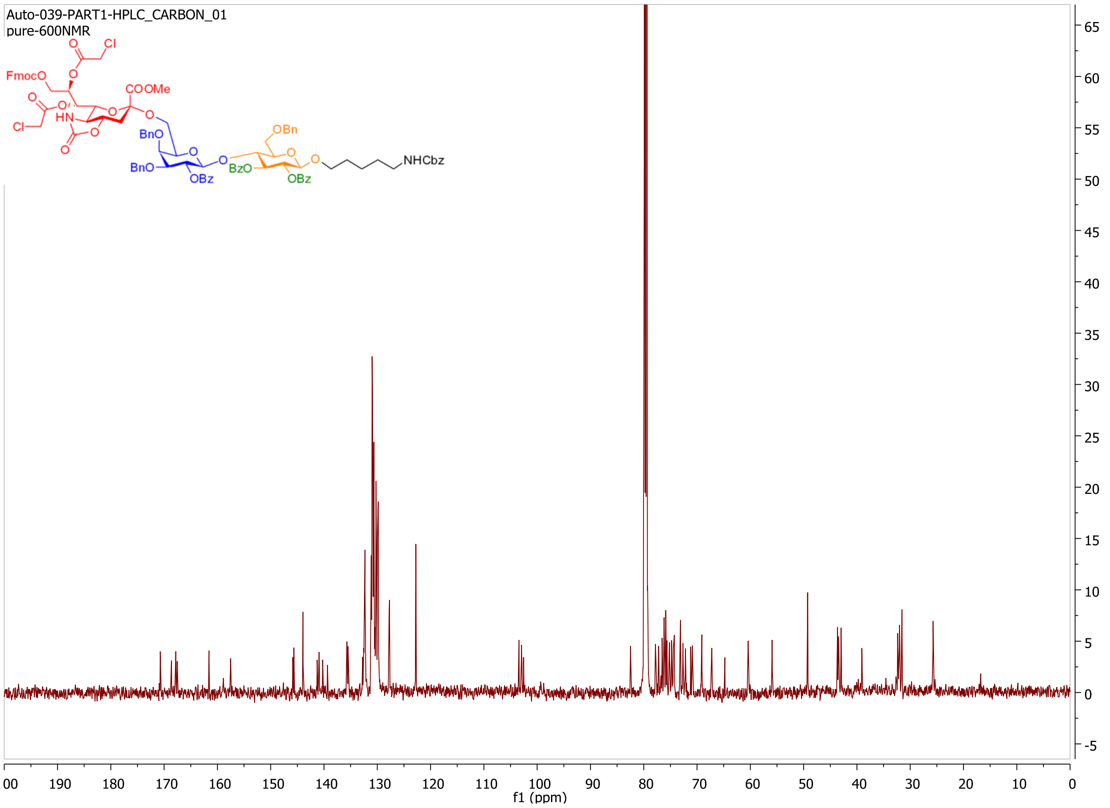

HSQC of 14

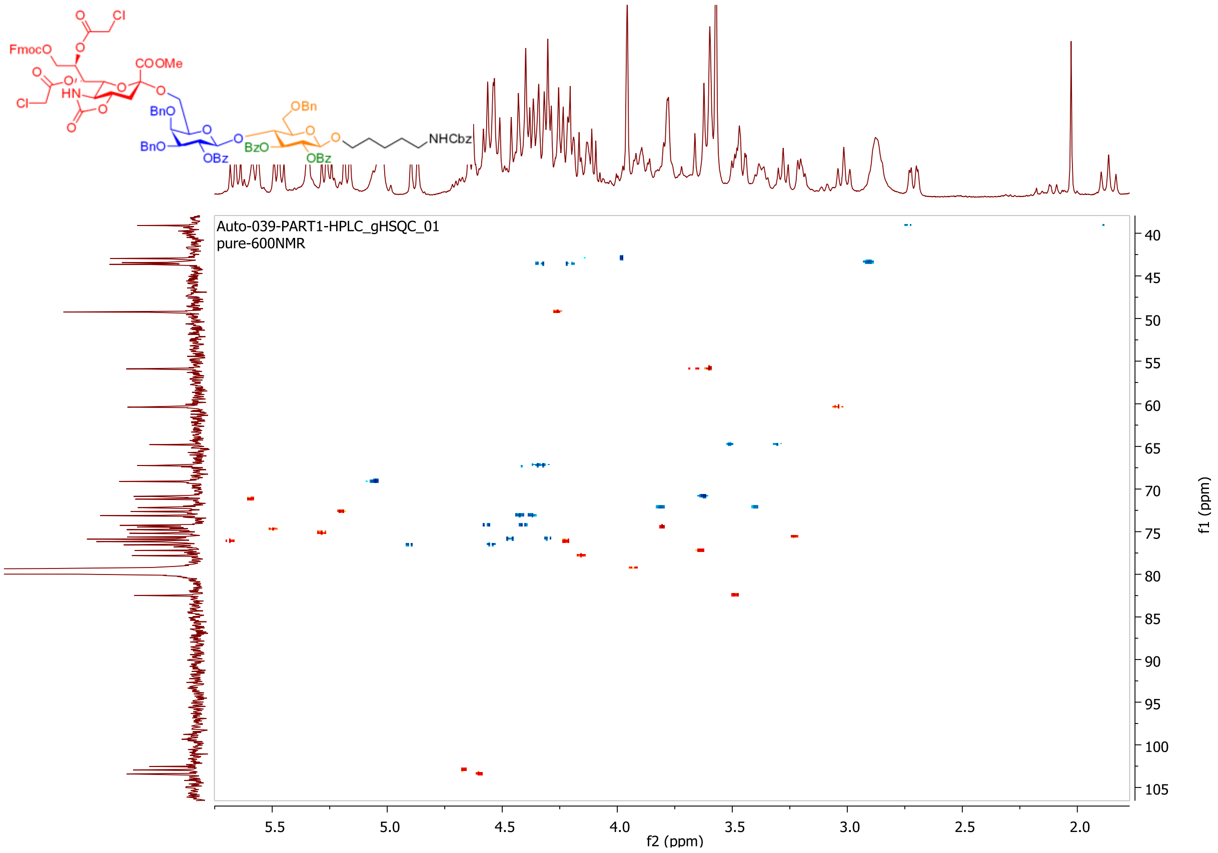

COSY of 14

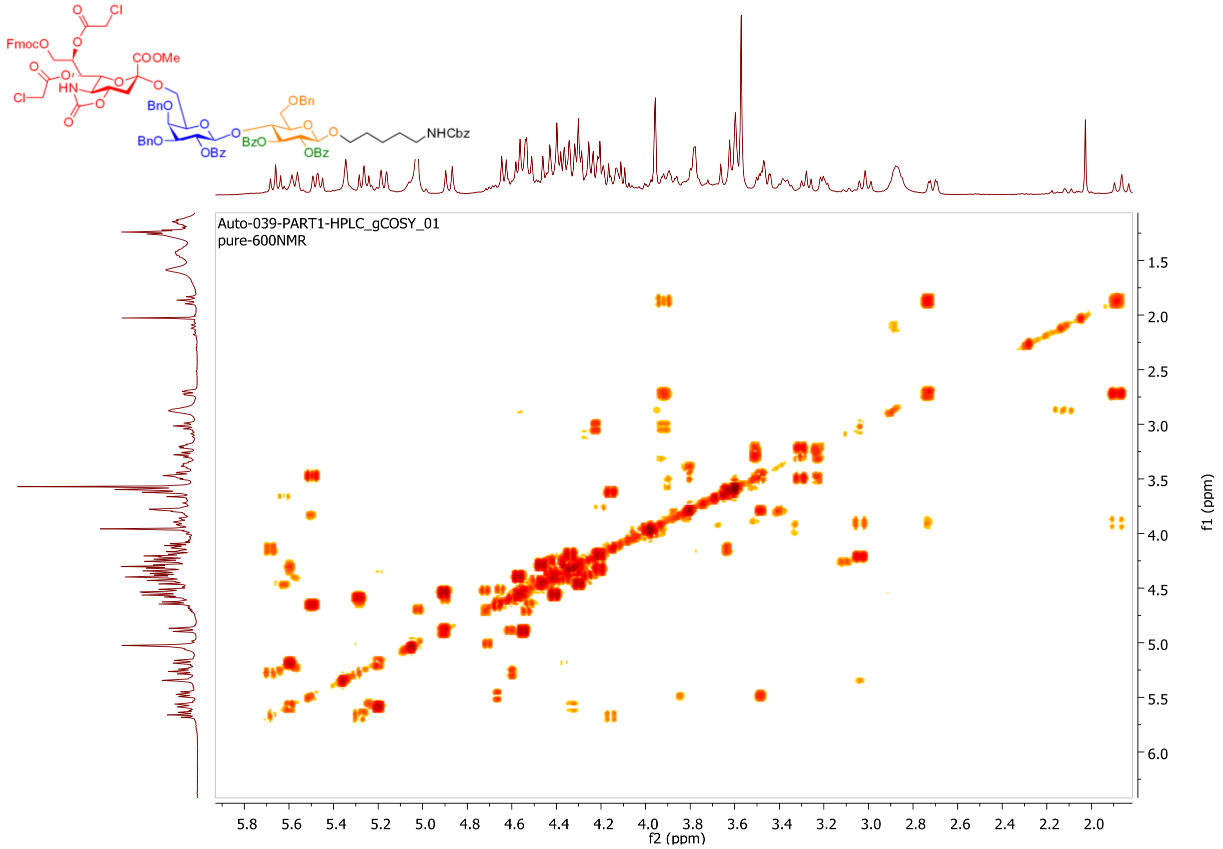

1D couple HMQC of 14

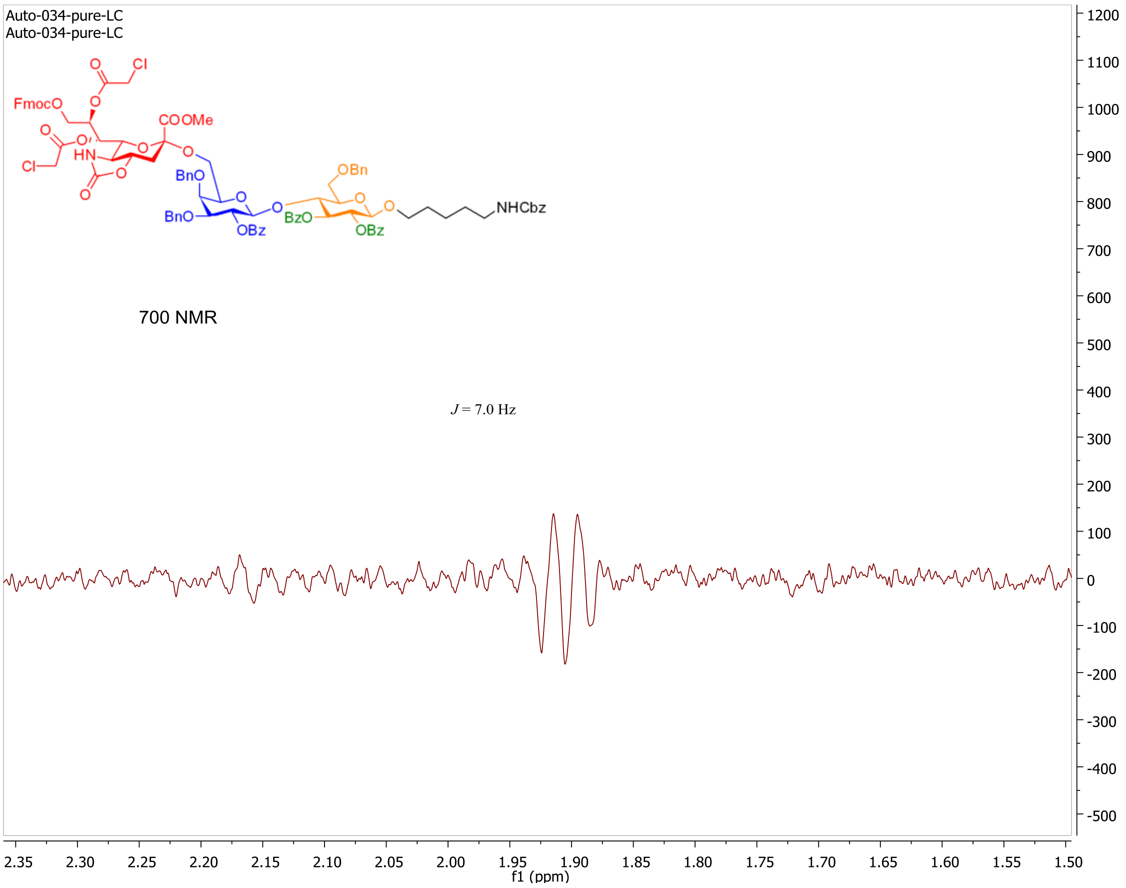

Automated Synthesis of 15

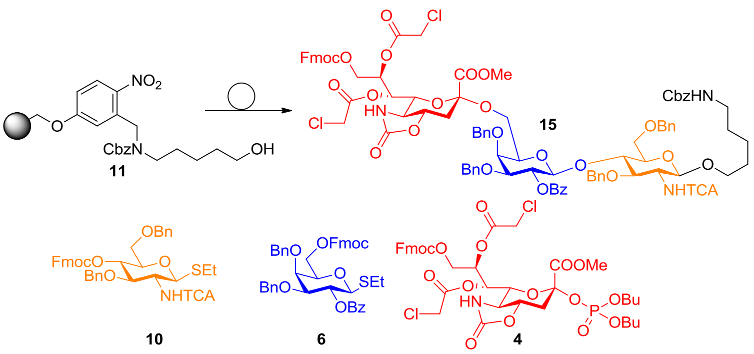

Reaction Condtions:

1) Glycosylation:
2 × 5 eq **10**, TfOH, NIS, DCM,
-30 °C for 5 min to -10 °C for 25 min.

2) Deprotection:
3 × 20 % NEt_3 in DMF, 5 min.

3) Glycosylation:
2 × 5 eq **6**, TfOH, NIS, DCM,
-40 °C for 5 min to -20 °C for 30 min.

4) Deprotection:
3 × 20 % NEt_3 in DMF, 5 min.

5) Glycosylation:
2 × 5 eq **4**, TMSOTf, ACN/DCM (1:1),
-50 °C (5 min),-30 °C (10 min), -20 °C (80 min), -10 °C (10 min), 0 °C (10 min)

6) Cleavage:
Photo-cleavage, UV

All the synthesis and purification details were identical as for **14**, except for changing the first glycoside build block **10** to obtain **15** in 7% (3.2 mg) overall yield based on the resin loading. *Analytical data for tri-saccharide **15**:* R_f, 0.78, EA/Hex (1/1); $[\alpha]_D^{20}$ = +10.68 (c = 0.33, $CHCl_3$); **IR** (thin film) ν_{max} = 2951, 1750, 1526, 1450, 1260, 1068 cm^{-1}; **^{1}H NMR** (400 MHz, $CDCl_3$) δ 7.93 (d, *J* = 7.8 Hz, 2H), 7.76 (d, *J* = 7.4 Hz, 2H), 7.57 (d, *J* = 7.2 Hz, 3H), 7.41 (m, 6H), 7.37 – 7.26 (m, 18H), 7.19 – 7.13 (m, 7H), 5.59 (t, *J* = 8.9 Hz, 1H, H-2 $_{Gal}$), 5.46 (d, *J* =

9.4 Hz, 1H, H-8_{sia}), 5.26 (s, 1H, NH_{sia}), 5.15 (d, J = 9.4 Hz, 1H, H-7_{sia}), 5.10 – 4.98 (m, 3H, CH_2-Cbz, CH_2-OBn×1), 4.93 (d, J = 11.2 Hz, 1H, CH_2-OBn×1), 4.76 – 4.54 (m, 7H, CH_2-OBn×4, H-1_{Gal}, H-1_{GlcN}, NH), 4.48 (d, J = 12.3 Hz, 1H, CH_2-OBn×1), 4.45 – 4.19 (m, 8H, CH_2-Fmoc×3, CH_2-OBn×1, H-9_{sia}×2, CH_2Cl×1, NH), 4.19 – 4.09 (m, 2H, CH_2Cl×1, H-6_{sia}), 4.05 – 3.97 (m, 4H, CH_2Cl×2, H-3_{GlcN}, H-5_{GlcN}), 3.94 – 3.77 (m, 3H, H-4_{Gal}, H-4_{sia}, H-6_{GlcN}×1), 3.74 – 3.56 (m, 7H, OCH_3×3, OCH_2-linker×1, H-2_{GlcN}, H-6_{Gal}×2), 3.55 – 3.35 (m, 4H, H-3_{Gal}, H-5_{Gal}, H-6_{GlcN}×1, H-4_{GlcN}), 3.24 (d, J = 9.2 Hz, 1H, OCH_2-linker×1), 3.10 (d, J = 6.1 Hz, 2H, CH_2NHCbz-linker×2), 2.81 (t, J = 10.4 Hz, 1H, H-5_{sia}), 2.73 (d, J = 12.8 Hz, 1H, H-$3_{eq,sia}$), 2.11 (t, J = 12.8 Hz, 1H, H-$3_{ax,sia}$), 1.50 – 1.32 (m, 6H); **^{13}C NMR** (1151 MHz, $CDCl_3$) δ 170.7, 170.7, 168.8, 167.8, 164.5, 161.7, 159.0, 157.6, 145.8, 145.6, 143.9, 141.1, 141.0, 140. 8, 140.1, 139.3, 135.9, 132.4, 131.2, 131.1, 131.1, 131.0, 130.9, 130.8, 130.7, 130.4, 130.2, 130.0, 127.7, 122.8, 102.7, 102.6, 102.0, 95.1, 82.2, 80.4, 79.5, 76.6, 76.1, 76.0, 75.1, 75.0, 74.5, 73.2, 72.8, 72.2, 71.1, 69.2, 67.3, 66.6, 60.2, 60.0, 56.0, 49.2, 43.7, 43.6, 43.1, 39.1, 32.2, 31.6, 25.9; **1D couple HMQC** (700 MHz, $CDCl_3$) $^3J_{C\text{-}1sia,\ H\text{-}3ax,sia}$ = 7.3 Hz; **ESI HR-MS**: m/z $[M+Na]^+$ calcd. for $C_{92}H_{94}Cl_5N_3\ O_{26}Na$ 1855.4466; Found 1855.4554.

Analytical NP-HPLC YMC-Pack-Sil of Crude triaccharide **15** (280nm trace)

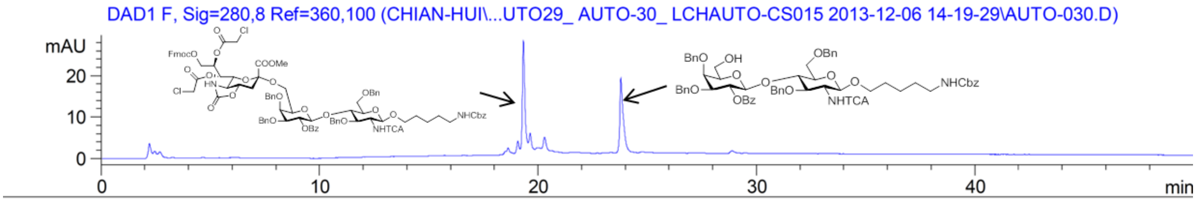

^{1}H NMR of 15

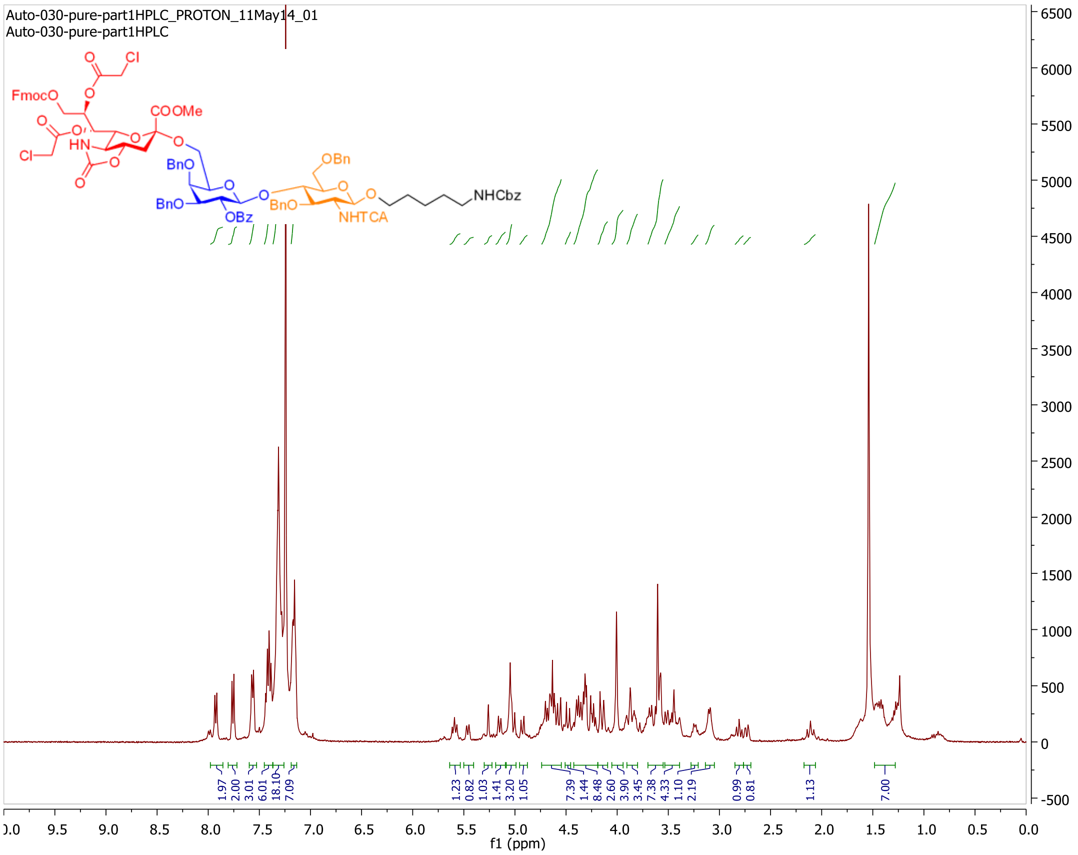

^{13}C NMR of 15

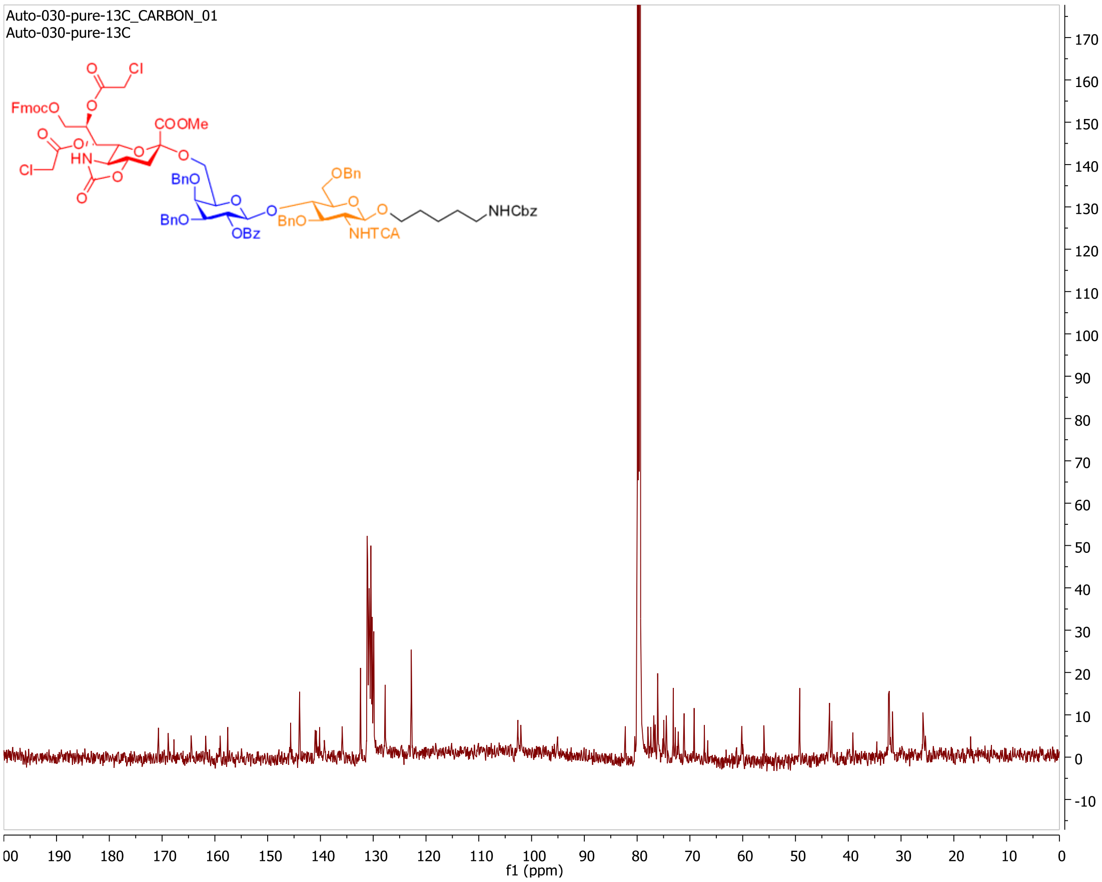

HSQC of 15

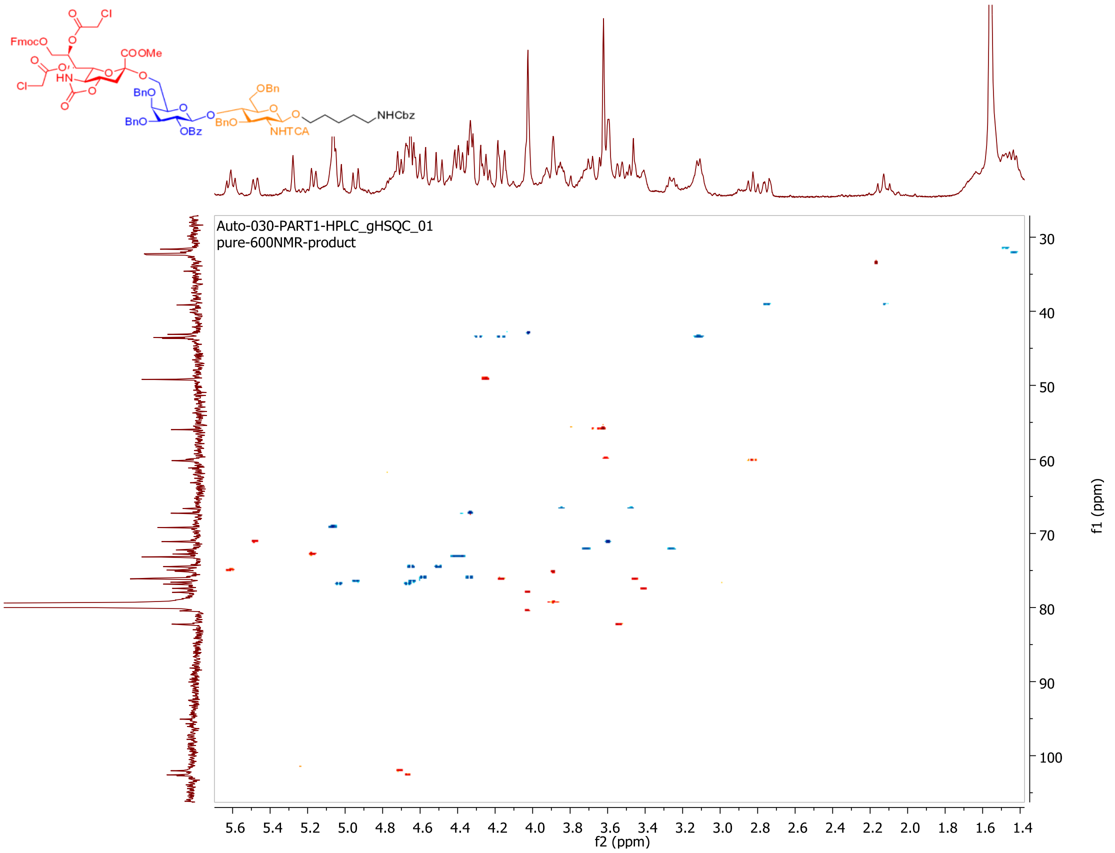

COSY of 15

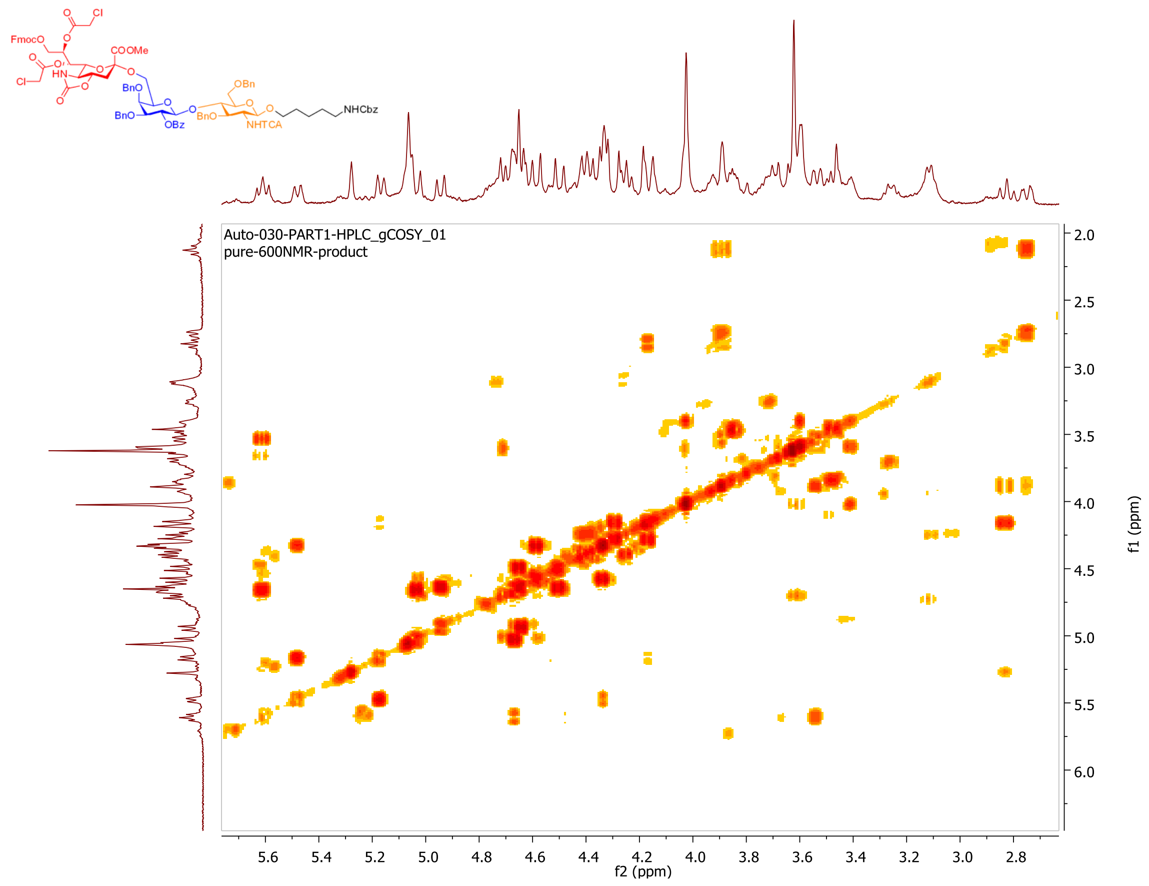

1D couple HMQC of 15

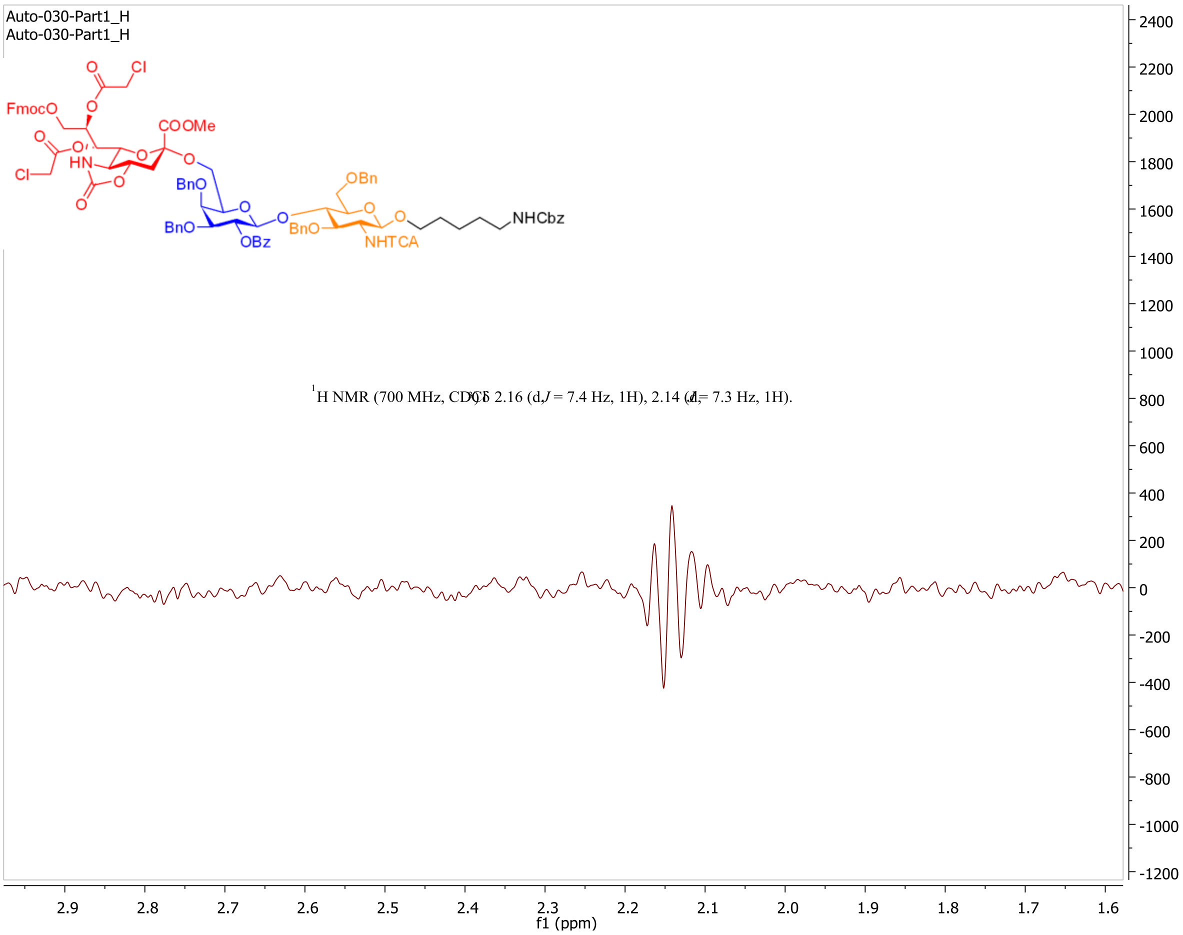

Automated Synthesis of 16

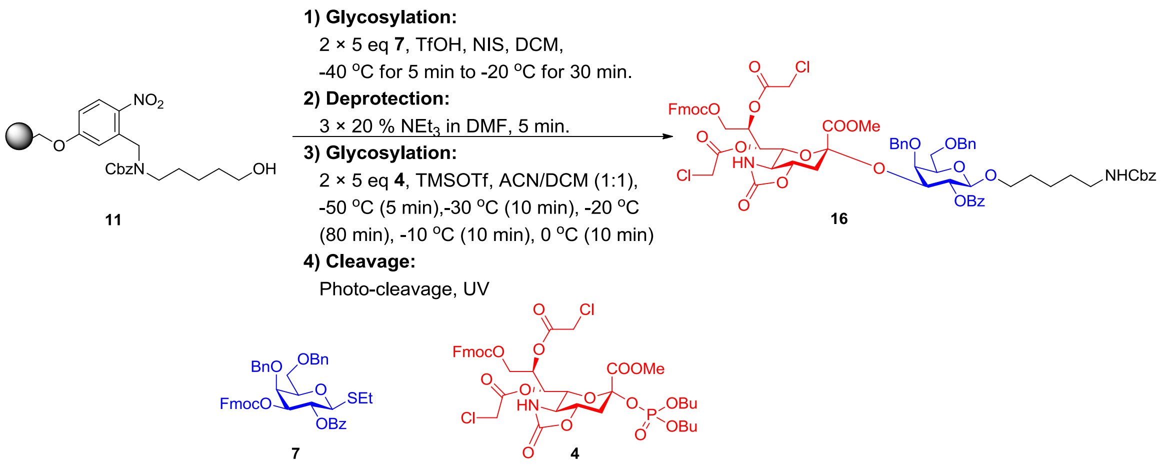

Table S5. Automated synthesis program for disaccharide **16**.

Steps	Automation process	Module
1	Preparation of the Resin Ready for Synthesis	A
2	Acidic Wash	B
3	Glycosylation: Donor **7**	C with NIS/ TfOH activation solution
4	Deprotection of Fmoc	D
5	Acidic Wash	B
6	Glycosylation: Donor **4**	C with TMSOTf activation solution

Cleavage, Analysis and Purification: Disaccharide **16** was cleaved from the solid support as described for Post-Synthesis Manipulations. The crude product was analyzed using normal phase analytical HPLC (YMC-Pack-Sil-NP; 5 μm, 150 mm, 4.6 mm; Linear gradient: EtOAc /Hexane; 10% EtOAc for 5 min, to 90% EtOAc in 30 min) and purified using preparative H PLC (YMC-Pack-Sil-NP; 5 μm, 150 mm, 20.0 mm, gradient: Hexane/EtOAc; 10-90% at 30 min) to obtain compound **16** (6.5 mg, 19% overall yield based on the resin loading).

*Analytical data for disaccharide **16**:* R_f, 0.62, EA/Hex (1/1); $[\alpha]_D^{20}$ = + 6.19 (c = 1.0 , $CHCl_3$); **IR** (thin film) ν_{max} = 2927, 2858, 1751, 1521,1498,1453,1408, 1378, 1267, 1154, 1097, 1073, 1027, 742, 712, 699, 677 cm^{-1}; **^{1}H NMR** (600 MHz, DMSO) δ 7.87 (dd, J = 15.0, 7.3 Hz, 3H), 7.58 (dd, J = 12.9, 7.6 Hz, 3H), 7.52 – 7.44 (m, 2H), 7.42 – 7.36 (m, 2H), 7.36 – 7.20 (m, 18H), 6.96 (s, 1H, N*H*), 5.44 (d, *J* = 7.9 Hz, 1H, H-8_{sia}), 5.28 – 5.14 (m, 2H, H-7_{sia}, H-2_{Gal}), 4.96 (s, 2H, CH_2-Fmoc), 4.84 (s, 2H, CH_2-Cbz), 4.74 (d, *J* = 11.4 Hz, 1H, CH_2-OBn×1), 4.69 (d, *J* = 10.3 Hz, 1H, H-9_{sia}), 4.60 (d, *J* = 11.4 Hz, 1H, CH_2-OBn×1), 4.57 (d, *J* = 7.6 Hz, 1H, H-1_{Gal}), 4.52 – 4.20 (m, 10H), 4.21 – 4.15 (m, 1H, H-6_{sia}), 4.00 (d, *J* = 13.2 Hz, 1H, H-4_{sia}), 3.91 (m, 2H, H-4_{Gal}, H-5_{Gal}), 3.68 – 3.52 (m, 3H, H-6_{Gal}, OCH_2-linker×1), 3.39 (t, *J* = 10.4 Hz, 1H, H-5_{sia}), 3.33 (s, 3H, OCH_3), 2.78 – 2.62 (m, 2H, CH_2NHCbz-linker ×2), 2.06 (t, *J* = 12.5 Hz, 1H, H-$3_{ax,sia}$), 1.18 – 0.79 (m, 6H); **^{13}C NMR** (151 MHz, DMSO) δ 166.8 (C=O), 166.6 (C=O), 166.3 (C=O), 165.2 (C=O), 158.9 (C=O), 155.9 (C=O), 154.2 (C=O), 143.2, 143.1, 140.7, 138.4, 138.0, 137.3, 133.3, 129.8, 129.3, 128.6, 128.3, 128.2, 128.2, 127.8, 127.7, 127.7, 127.6, 127.5, 127.2, 127.2, 127.1, 125.0, 124.9, 120.2, 100.4 (C-1_{Gal}), 99.4(C-1_{Sia}), 79.2, 75.5, 74.3, 73.0, 72.4, 72.3, 72.0, 71.8, 71.3, 69.2, 68.8, 68.5, 65.3, 65.1 (C-9_{sia}), 59.8, 56.2 (C-5_{sia}), 52.6 (OCH_3), 46.2 (*C*H-OFmoc), 41.0 X 2 (*C*H_2Cl) , 40.1 (C-3_{sia}), 28.9, 28.6, 22.5; **ESI HR-MS**: m/z $[M+Na]^+$ calcd. for $C_{70}H_{72}Cl_2N_2O_{21}Na$ 1369.3902; Found 1369.3875.

Analytical NP-HPLC YMC-Pack-Sil of Crude triaccharide **16**(280 nm trace)

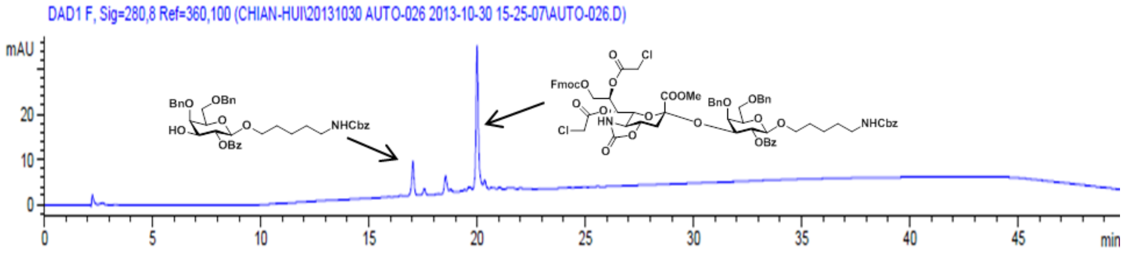

^{1}H NMR of 16

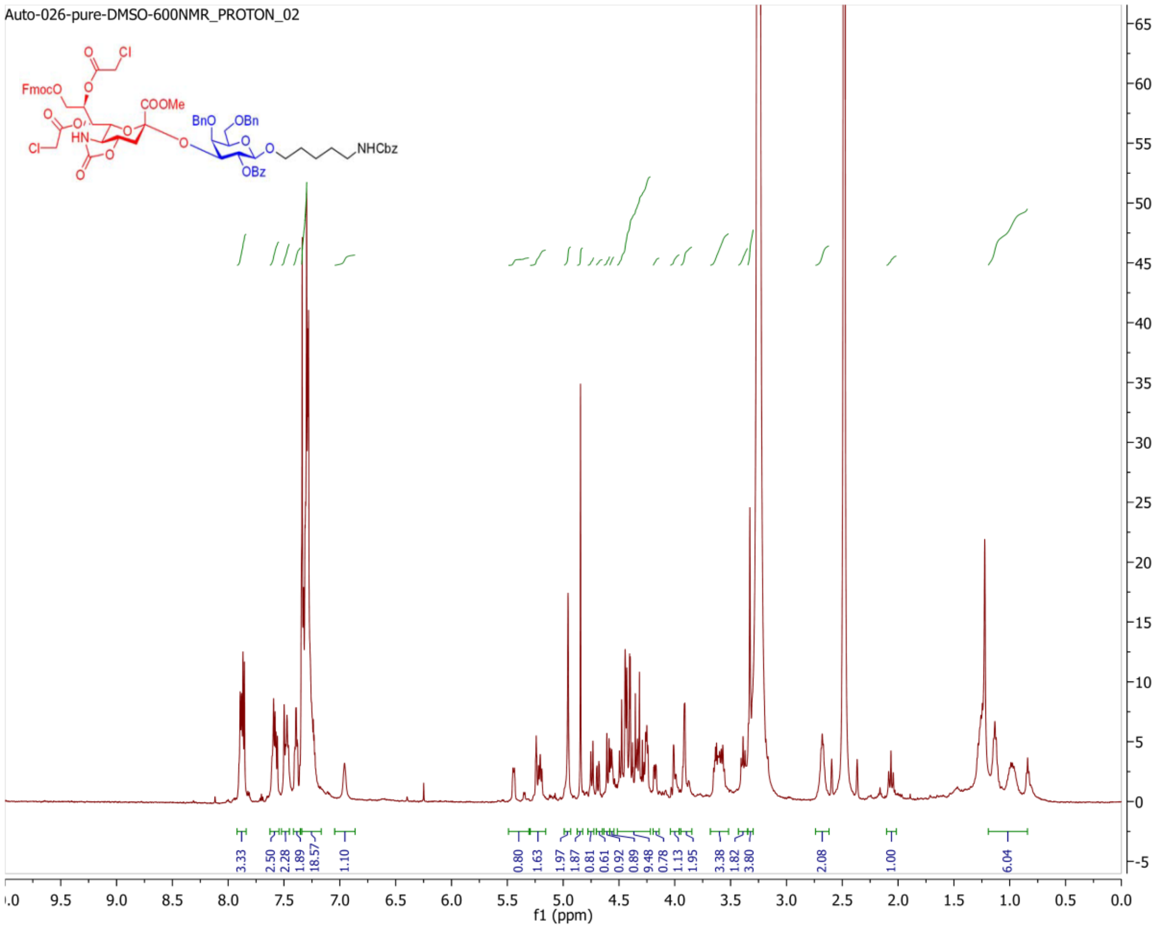

^{13}C NMR of 16

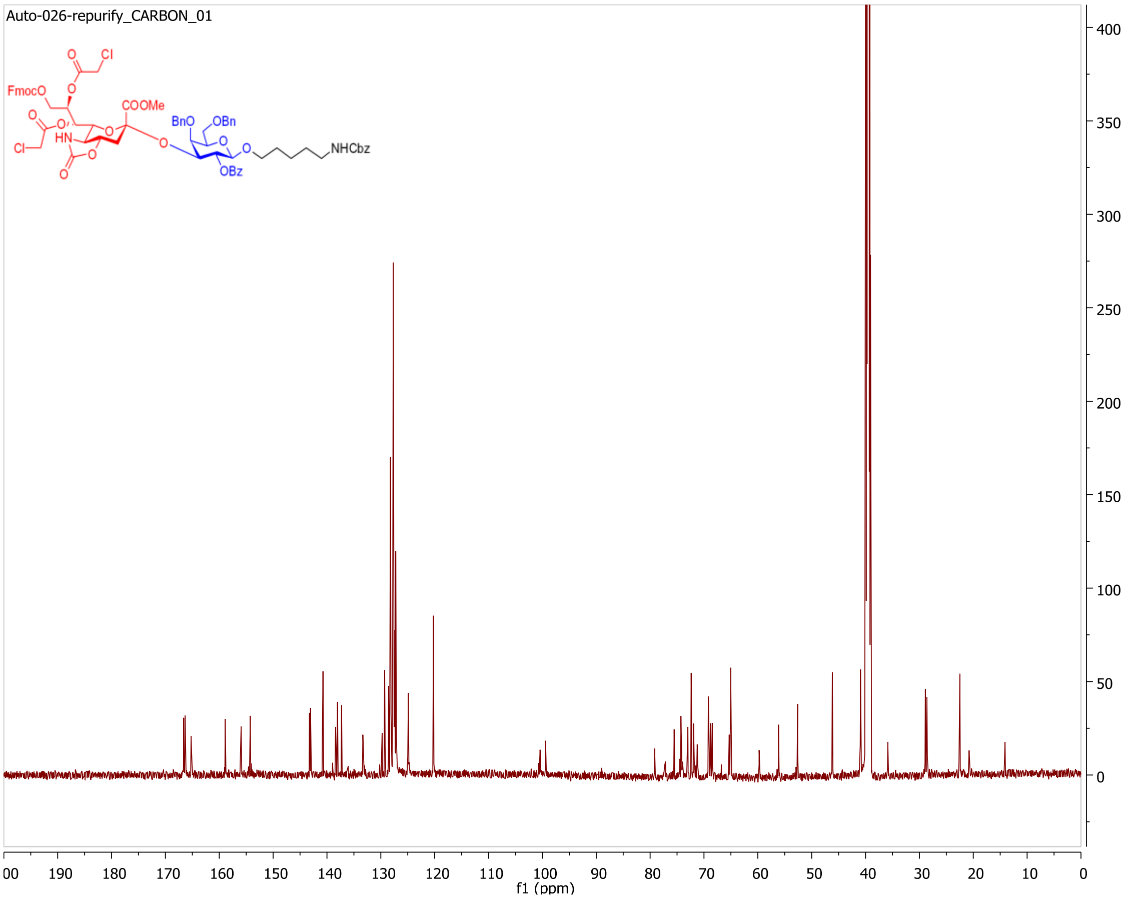

HSQC of 16

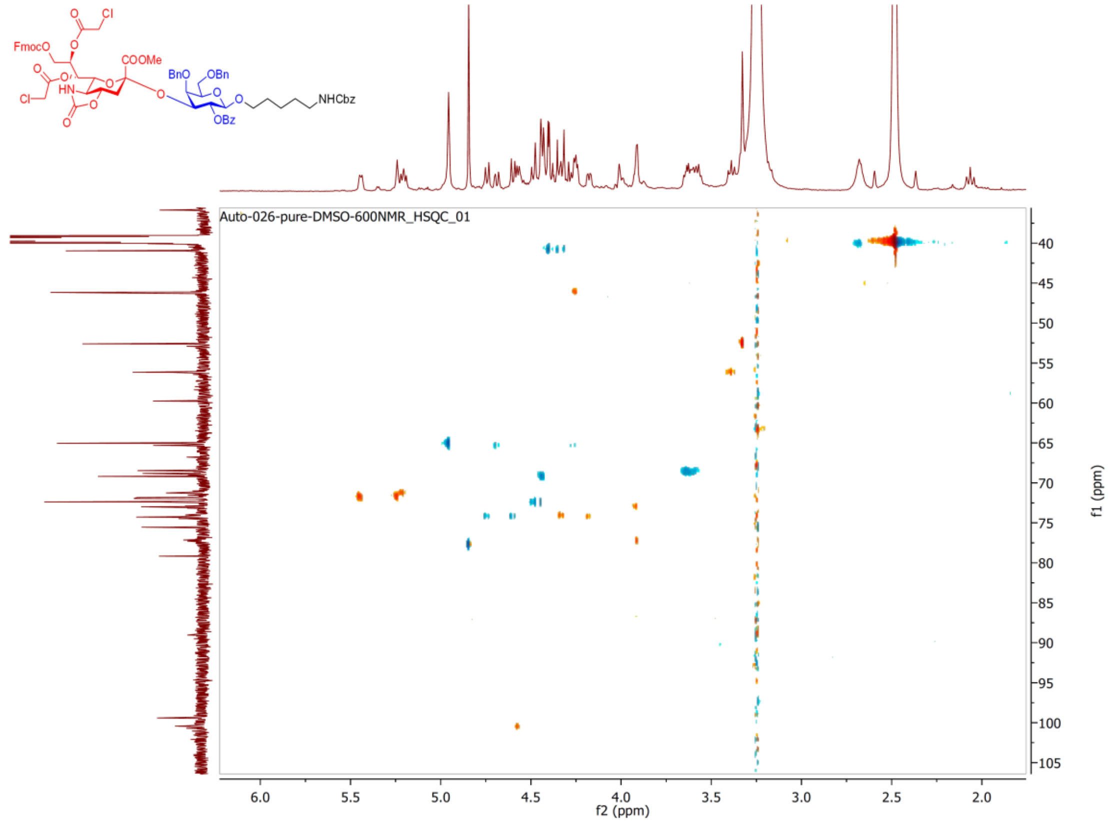

COSY of 16

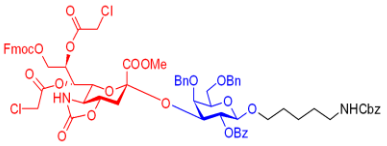

FmocO
Cl
COOMe
BnO
OBn
HN
NHCbz
OBz
Cl

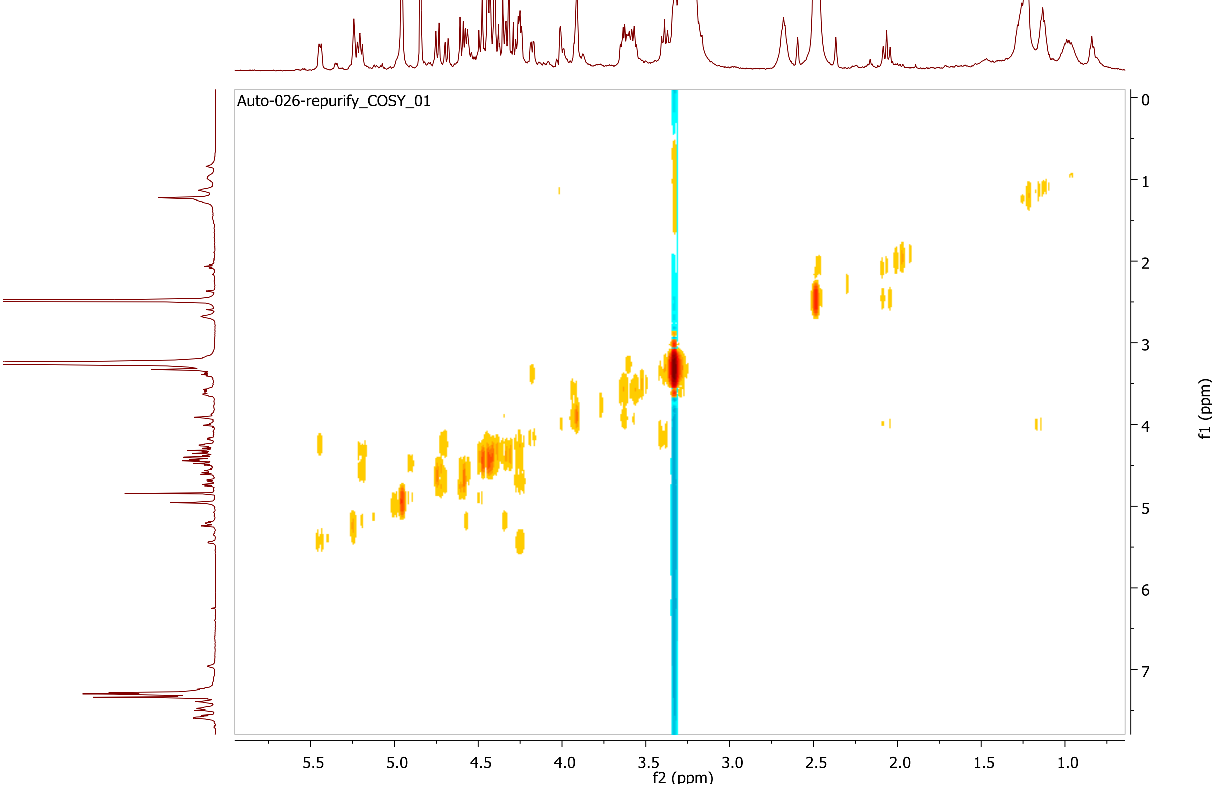

Auto-026-repurify_COSY_01
f2 (ppm)
f1 (ppm)

Discussion of first glycosylation: *cis*-glycosidic linkage for 17

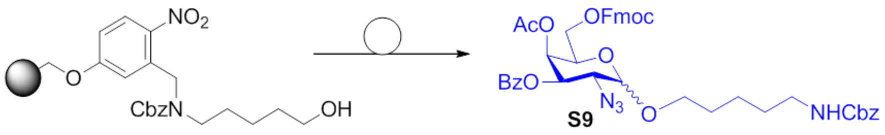

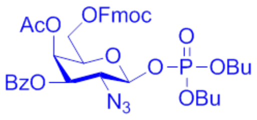

8

Glycosylation Condtion: :
2 × 5 eq, TMSOTf, DCM/Dioxane

Photo-Cleavage Condtion:
UV irradiation using continuous flow reactor, DCM, RT.

	Temp.	Solvent	α/ β
1	0 °C (60 min), 20 °C (30 min)	DCM	2: 3
2	0 °C (60 min), 20 °C (30 min)	DCM/Dioxane (4/1)	2: 2
3	20 °C (90 min)	DCM/Dioxane (4/1)	2: 1.5
4	20 °C (90 min)	DCM/Dioxane (3/2)	2: 1

Table S6. Different solvents and temperature conditions for the first α-GalN to linker glycosylation in synthesis of precursor of **17**.

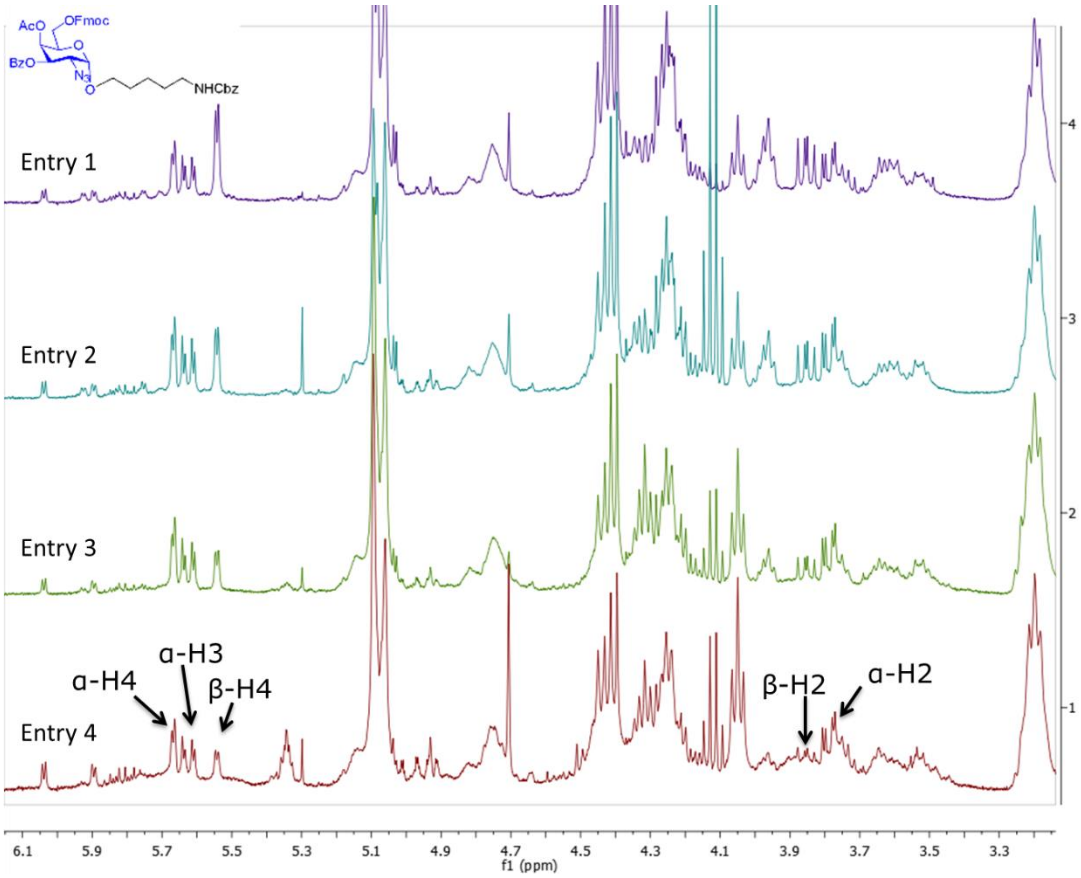

Figure S1. The NMR spetrum of crude **S9** in different conditions for first glycosylation.

Automated Synthesis of 17

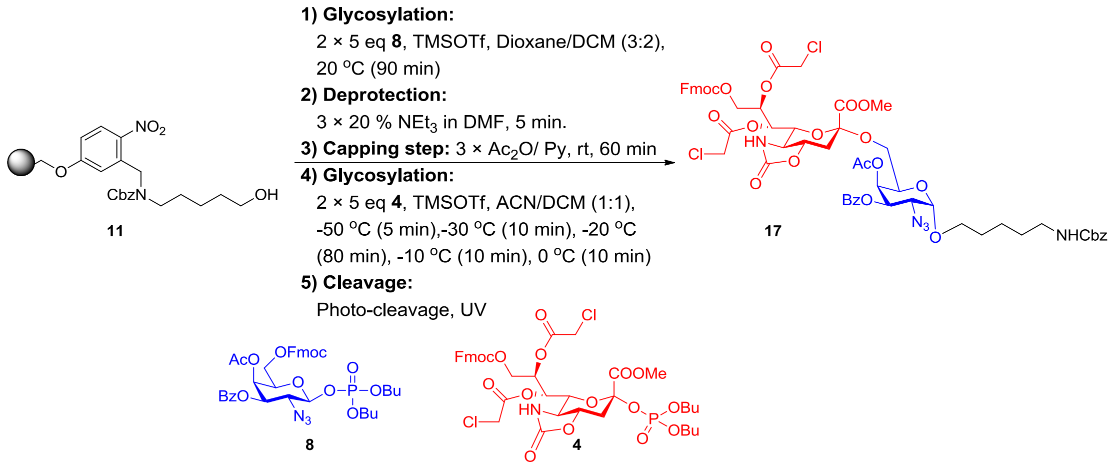

Table S7. Automated synthesis program protocol for disaccharide **17**.

Steps	Automation process	Module
1	Preparation of the Resin Ready for Synthesis	A
2	Acidic Wash	B
3	Glycosylation: Donor **8**	C with TMSOTf activation solution
4	Capping	E
5	Deprotection of Fmoc	D
6	Acidic Wash	B
7	Glycosylation: Donor **4**	C with TMSOTf activation solution

Note: Fmoc quantifiction 88.6% for first glycosylation.

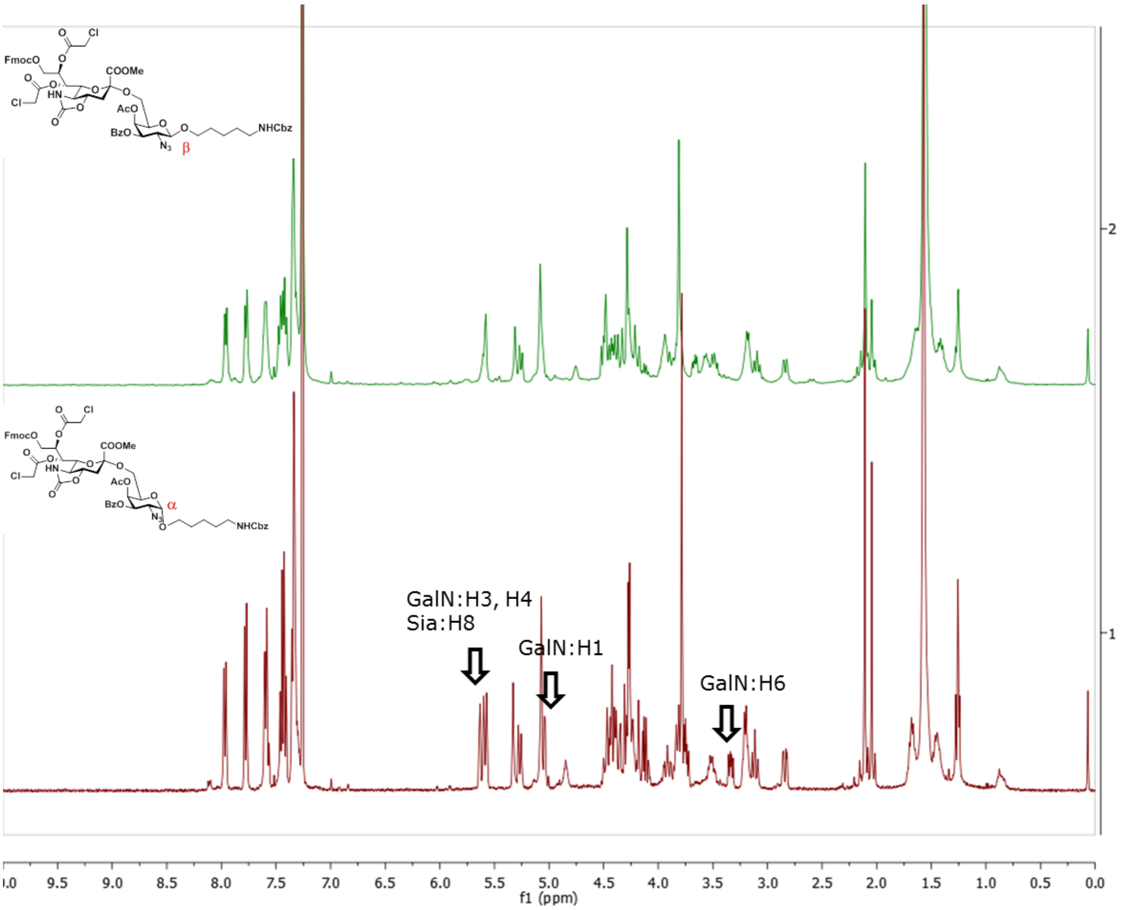

Figure S2. The comparison ^{1}H-NMR spectrum of **17** and side its side product (β-GalN to linker).

Cleavage, Analysis and Purification: di-saccharide **17** was cleaved from the solid support as described for Post-Synthesis Manipulations. The crude product was analyzed using normal phase analytical HPLC (Luna-silica-NP; 5 µm, 250 mm, 4.6 mm; Linear gradient: EtOAc /Hexane; 20% EtOAc for 5 min, to 70% EtOAc in 30 min) and purified using semi-preparative HPLC (Luna-silica-NP; 5 µm, 150 mm, 10.0 mm, gradient: Hexane/EtOAc; 20-70% in 30 min) to obtain compound **17** (3 mg, 10% overall yield based on the resin loading; undesired Beta form to *O*-linker, 0.9 mg, 3%).

Analytical data for disaccharide ***17****:* **$[\alpha]_D^{20}$** = + 9.79 (c = 0.38, $CHCl_3$); **IR** (thin film) ν_{max} = 2928, 2112, 1749, 1453, 1264, 1150, 1017, 760, 713 cm^{-1}; **^{1}H NMR** (600 MHz, $CDCl_3$) δ 7.95 (d, J = 7.9 Hz, 2H), 7.76 (d, J = 7.6 Hz, 2H), 7.62 – 7.53 (m, 3H), 7.48 – 7.36 (m, 4H), 7.35 – 7.26 (m, 7H), 5.62 (d, J = 2.3 Hz, 1H, H-4$_{GalN}$), 5.57 (d, J = 3.2 Hz, 1H, H-3$_{GalN}$), 5.56 (d, J = 3.2 Hz, 1H, H-8$_{sia}$), 5.30 (s, 1H, N*H*$_{sia}$), 5.25 (dd, J = 10.0, 1.8 Hz, 1H, H-7$_{sia}$), 5.05 (s, 2H, C*H*$_2$-Cbz), 5.02 (d, J = 3.2 Hz, 1H, H-1$_{GalN}$), 4.82 (b, 1H, N*H*Cbz), 4.48 – 4.34 (m, 4H, H-9$_{sia}$×2, C*H*$_2$-Fmoc), 4.33 – 4.11 (m, 7H, C*H*$_2$Cl×4, H-6$_{sia}$, C*H*-Fmoc, H-5$_{GalN}$), 3.93 – 3.87 (m, 1H, H-4$_{sia}$), 3.83 – 3.79 (m, 1H, OC*H*$_2$-linker×1), 3.77 (s, 3H, OC*H*$_3$), 3.76 – 3.70 (m, 2H, H-2$_{GalN}$, H-6$_{GalN}$ ×1), 3.53 – 3.44 (m, 1H, OC*H*$_2$-linker×1), 3.32 (dd, J = 10.6, 6.5 Hz, 1H, H-6$_{GalN}$ ×1), 3.22 – 3.14 (m, 2H, C*H*$_2$NHCbz-linker×2), 3.10 (t, J = 10.3 Hz, 1H, H-5$_{sia}$), 2.82

(dd, J = 12.0, 3.5 Hz, 1H, H-$3_{eq,sia}$), 2.09 (s, 3H, OCH_3), 2.03 (t, J = 12.0 Hz, 1H, H-$3_{ax,sia}$), 1.71 – 1.64 (m, 2H), 1.62 – 1.46 (m, 2H), 1.45 – 1.39 (m, 2H); **^{13}C NMR** (151 MHz, $CDCl_3$) δ 169.7 (C=O), 168.3 (C=O×2), 166.3 (C=O), 165.4 (C=O), 158.9 (C=O), 156.40 (C=O), 154.90 (C=O), 143.1, 143.0, 141.3, 136.6, 133.5, 129.7, 129.2, 128.5, 128.5, 128.1, 128.0, 127.2, 127.2, 125.1, 125.1, 120.1, 100.3 (C-2_{sia}), 98.3 (C-1_{GalN}), 76.5 (C-4_{sia}), 73.8 (C-6_{sia}), 70.5 (CH_2-OFmoc), 70.0 (C-7_{sia}), 69.0 (C-3_{GalN}), 68.7 (C-6_{GalN}), 68.0 (C-8_{sia}), 67.9 (×2, C-4_{GalN}, C-5_{GalN}), 66.6 (*C*-Cbz), 64.9 (C-9_{sia}), 64.3 (C-6_{GalN}), 57.9 (C-2_{GalN}), 57.5 (C-5_{sia}), 53.4 (OCH_3), 46.6 (*C*H-OFmoc), 41.0 (CH_2Cl), 40.9 (CH_2NHCbz-linker), 40.4 (CH_2Cl), 37.2 (C-3_{sia}), 29.6, 28.9, 23.3, 20.6 (*C*, OAc); **ESI HR-MS**: m/z $[M+Na]^+$ calcd. for $C_{58}H_{61}Cl_2N_5O_{21}Na$ 1256.3134; Found 1256.3107.

Analytical NP-HPLC Luna-silica of Crude triaccharide **17** (280 nm trace)

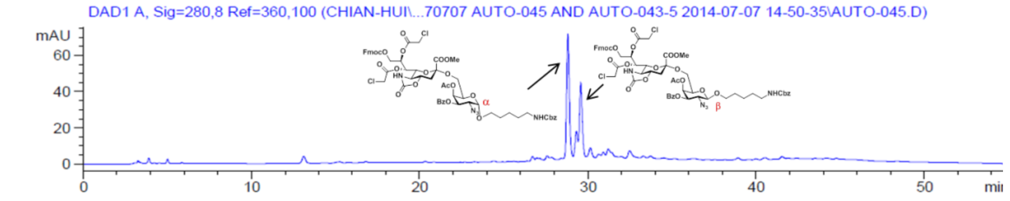

^{1}H NMR of 17

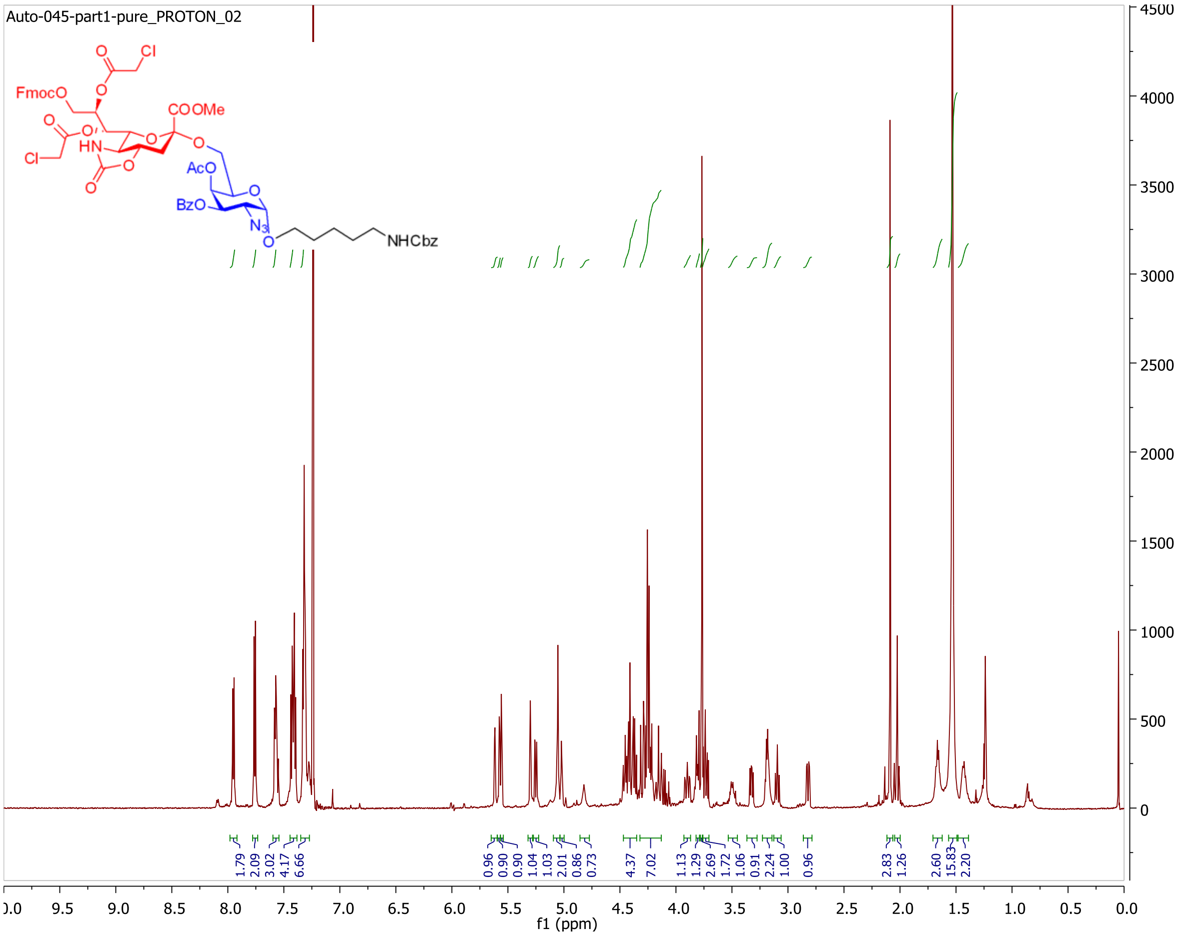

^{13}C NMR of 17

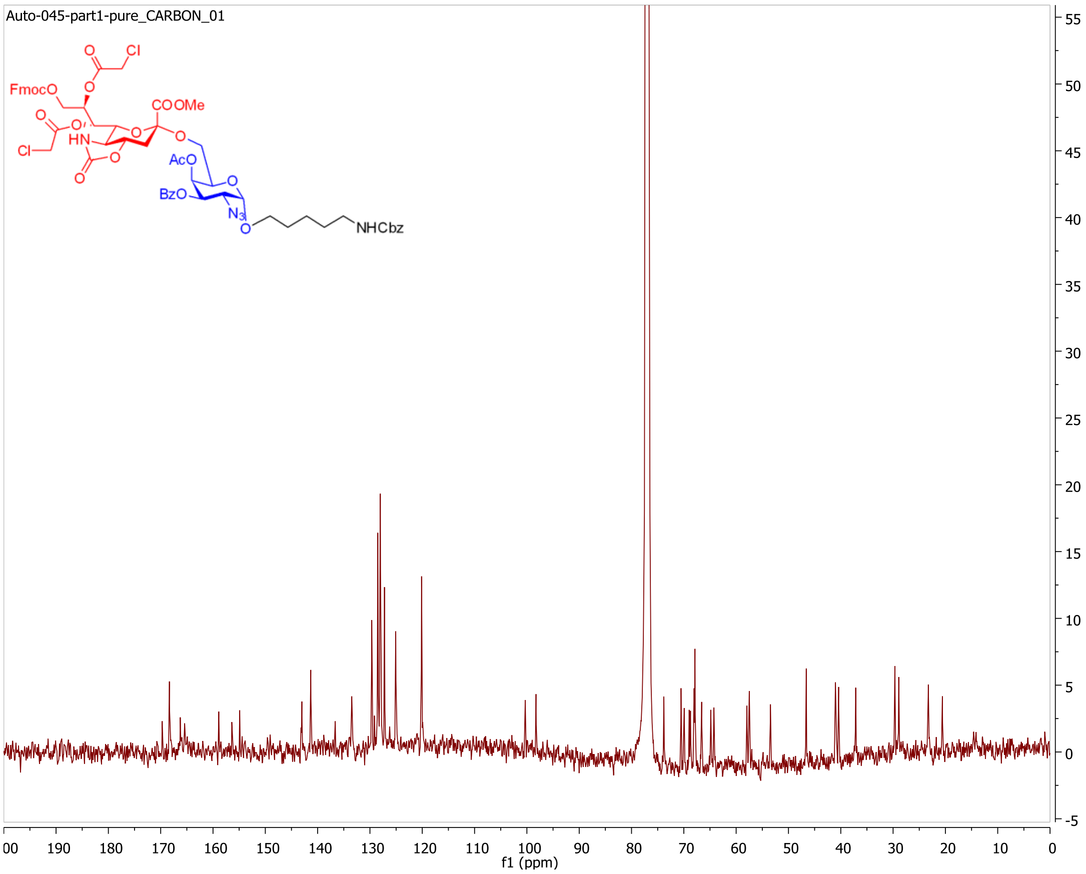

HSQC of 17

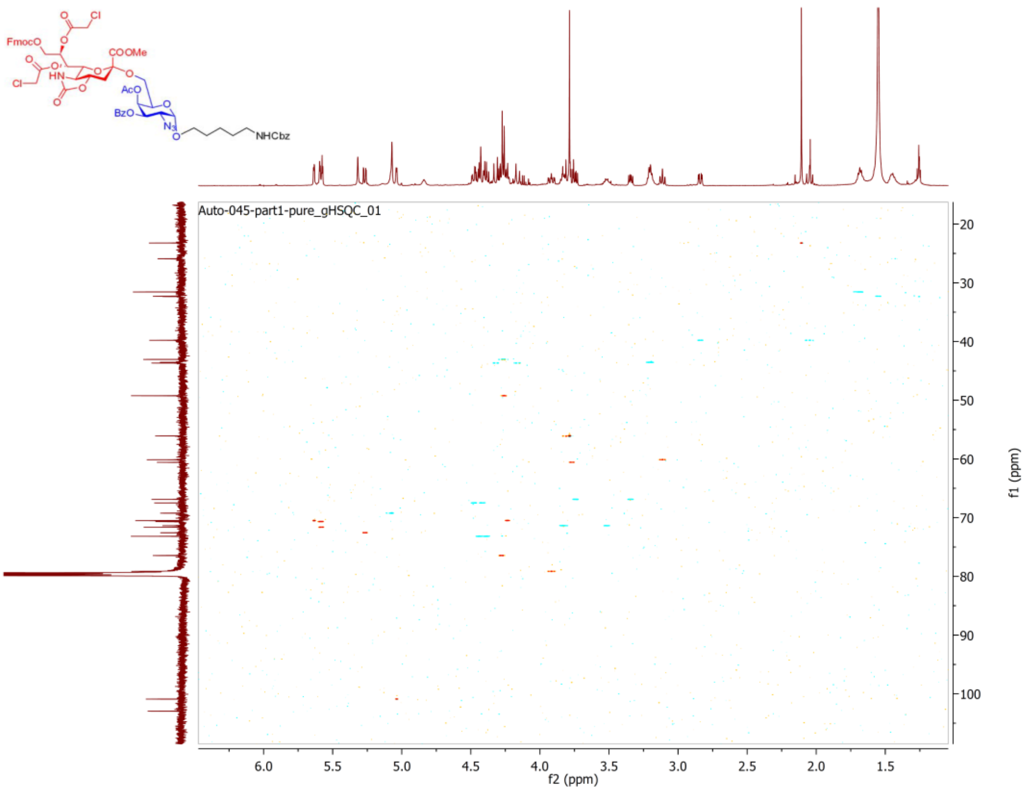

COSY of 17

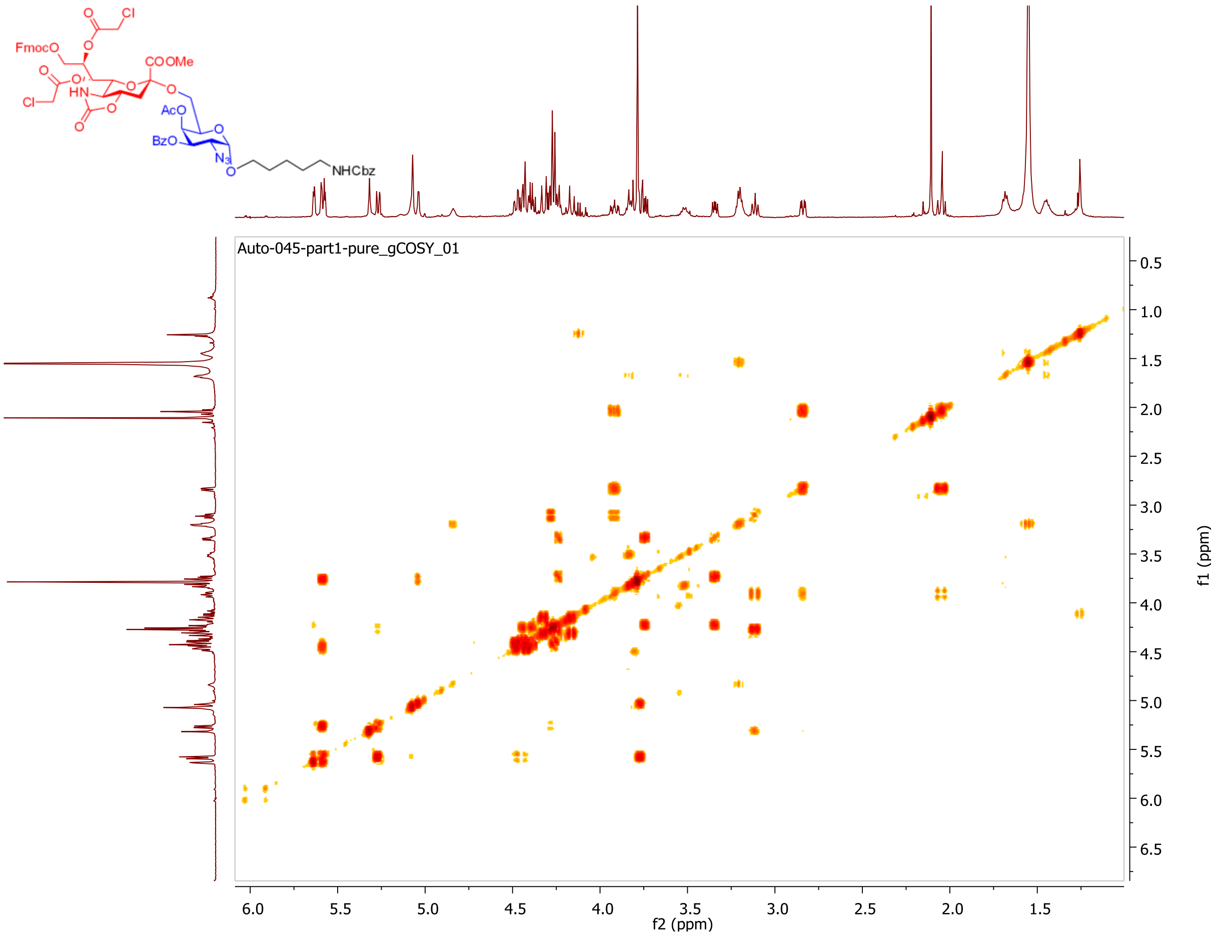

References

1. Wang, C.-H.; Li, S.-T.; Lin, T.-L.; Cheng, Y.-Y.; Sun, T.-H.; Wang, J.-T.; Cheng, T.-J. R.; Mong, K. K. T.; Wong, C.-H. and Wu, C.-Y. *Angew. Chem., Int. Ed.***2013**, 52, 9157-9161.
2. Chu, K.-C.; Ren, C.-T.; Lu, C.-P.; Hsu, C.-H.; Sun, T.-H.; Han, J.-L.; Pal, B.; Chao, T.-A.; Lin, Y.-F.; Wu, S.-H.; Wong, C.-H.; Wu, C.-Y. *Angew. Chem., Int. Ed.* **2011**, *50*, 9391-9395.
3. Eller, S.; Collot, M.; Yin, J.; Hahm, H. S.; Seeberger, P. H. *Angew. Chem., Int. Ed.* **2013,** *52*, 5858-5861
4. Kandasamy, J.; Schuhmacher, F.; Hahm, H. S.; Klein, J. C.; Seeberger, P. H. *Chem. Commun.* **2014,** 1875-1877.
5. Krock, L.; Esposito, D.; Castagner, B.; Wang, C.-C.; Bindschadler, P.; Seeberger, P. H. *Chem. Sci.* **2012,** *3*, 1617-1622.
6. Calin, O.; Eller, S.; Seeberger, P. H. *Angew. Chem., Int. Ed.***2013,** *52*, 5862-5865.
